# Methylation of Immune-Related Genes in Peripheral Blood Leukocytes and Breast Cancer

**DOI:** 10.3389/fonc.2022.817565

**Published:** 2022-02-10

**Authors:** Tian Tian, JinMing Fu, DaPeng Li, YuPeng Liu, HongRu Sun, Xuan Wang, XianYu Zhang, Ding Zhang, Ting Zheng, Yashuang Zhao, Da Pang

**Affiliations:** ^1^ Department of Epidemiology, Public Health College, Harbin Medical University, Harbin, China; ^2^ Department of Breast Surgery, Harbin Medical University Cancer Hospital, Harbin, China

**Keywords:** breast cancer, immune, DNA methylation, peripheral blood leukocytes, risk

## Abstract

Abnormal DNA methylation contributes to breast cancer (BC). Immune-related genes play crucial roles in BC development and progression. This study aims to investigate the effect of methylation of immune-related genes in peripheral blood leukocytes (PBLs) on BC risk. GSE51032 and GSE104942 datasets were used to identify significantly differentially methylated CpG sites (DMCs) of immune-related genes. A case-control study was conducted using MethylTarget sequencing to validate the relationship between the methylation levels of the screened genes and BC risk. We also evaluated the association between methylation haplotypes of screened genes and BC risk. Moreover, we sorted the blood leukocytes into T cells, B cells, and monocytes to detect the difference of DNA methylation in different cell subtypes. A total of five DMCs were screened from GEO datasets, including cg01760846 (*PSMC1*), cg07141527 (*SPPL3*), cg15658543 (*CARD11*), cg21568368 (*PSMB8*), and cg24045276 (*NCF2*). In the case-control study, there were significant associations between methylation of the CpG sites in the five genes and BC risk. Methylation haplotype burdens of *PSMC1*, *CARD11*, and *PSMB8* were associated with reduced BC risk. Moreover, there were heterogeneities in the methylation levels of the genes in different cell subtypes. In conclusion, methylation of *PSMC1*, *SPPL3*, *CARD11*, *PSMB8*, and *NCF2* in PBLs were associated with BC risk. The five-gene methylation could be the potential biomarkers for predicting BC risk.

## Introduction

Breast cancer (BC) is one of the most common malignant tumors in women worldwide, accounting for an estimated 2,261,419 new cases and 684,996 cancer deaths in 2020 (1). In China, BC ranks first in terms of incidence and fifth in terms of mortality in women, with an increasing trend in both incidence and mortality (2).

Accumulating evidence proves that the occurrence and progression of BC result from environmental factors and genetic and epigenetic alterations (3). DNA methylation, as one of the essential epigenetic modifications, has an important impact on normal cell physiology (4, 5). Changes in DNA methylation involve focal hypermethylation and global hypomethylation. Inappropriate DNA methylation can lead to aberrant transcriptional regulation that affects the expression patterns of the crucial genes involved in cellular proliferation and differentiation (6). Abnormalities in DNA methylation may cause various diseases, including BC. Moreover, it has been proposed that abnormal methylation often occurs in the early stage of cancer development and correlates with cancer predisposition (6, 7).

Immune system plays a vital role in cancer biology. The hypothesis of “cancer immunoediting” proposed by Schreiber indicates that the immune system can both eliminate tumor cells and promote tumor growth (8). The process of cancer immunoediting includes three phases: elimination, equilibrium, and escape, involving complex regulatory mechanisms and cooperation of various immune cells and molecules in the process of immune response. Dysregulated immune system can help tumor cells escape from immune surveillance and inextricably relate to tumor growth. The causes that can lead to immune system dysregulation include the loss of major histocompatibility complex (MHC) class I, defects in antigen processing and T-cell receptor (TCR) signaling and abnormal regulation of immune checkpoint, relating to disorders of various immune-related genes (9–11). Studies suggested that the functional status of immune system is implicated in BC risk and prognosis. Moreover, a growing numbers of evidence revealed that aberrant DNA methylation could influence the crucial immune processes and facilitate evasion of immunosurveillance through regulating the expression of immune genes (12). Therefore, it is necessary to explore the effects of DNA methylation in immune-related genes on BC risk and discover the potential biomarkers for BC.

To now, many studies focused on the relationship between the methylation of immune-related genes and BC in tumor tissues and cell lines. Compared with tissues, blood sampling is accessible and noninvasive, which makes it more readily to assess tumor risk and prognosis in population-based studies. The patterns of DNA methylation in peripheral blood leukocytes (PBLs) can be the potential epigenetic biomarkers for the early detection, risk assessment, and prognosis evaluation of BC.

We therefore carried out this study to explore the relationship between methylation of immune-related genes in PBLs and BC risk from the public data of the GEO database and the results of targeted sequencing in a case-control study. We also explored the association between DNA methylation haplotypes of the genes and BC risk. Moreover, considering the heterogeneity of DNA methylation in different cell types, we sorted the cells into T cells, B cells, and monocytes to detect the difference of DNA methylation in cell subtypes.

## Materials and Methods

### Data Source of Public Data

**Figure 1:**
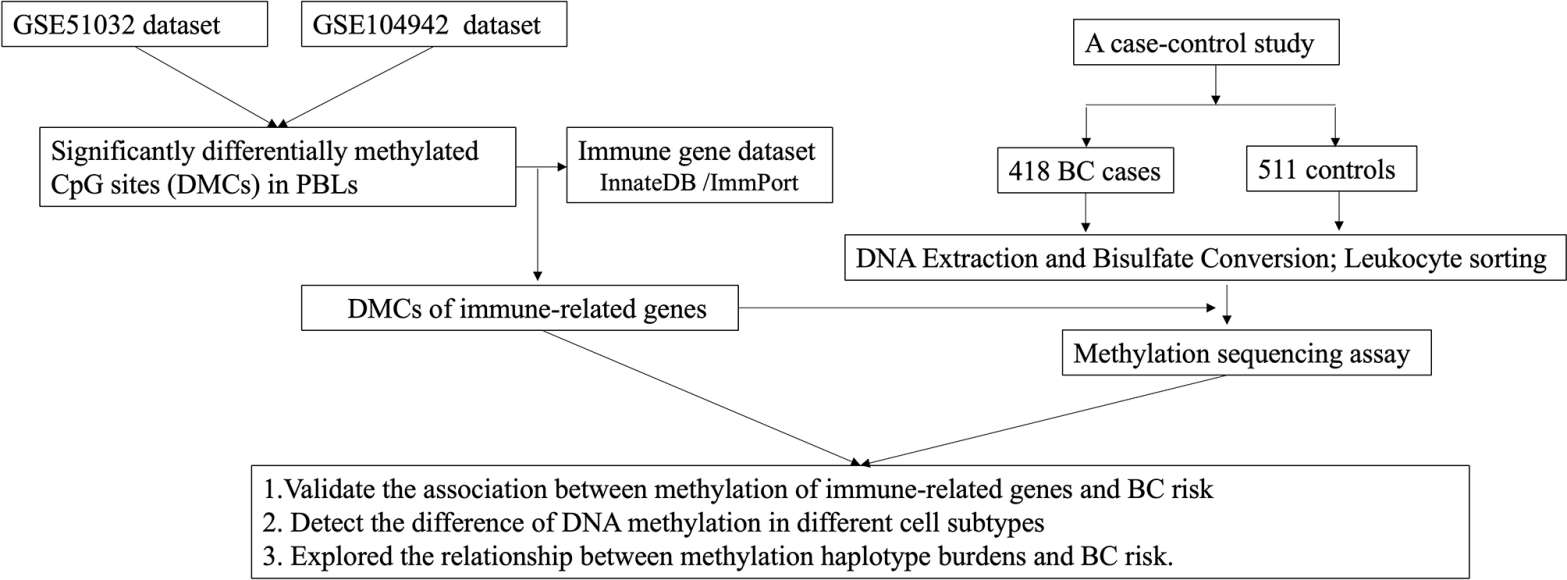
Flow chart of the study.

The workflow of the study is summarized in **Figure 1**. The public data of methylation in peripheral blood leukocytes for BC were obtained from the GEO database (https://www.ncbi.nlm.nih.gov/geo/) with the GEO accession numbers GSE51032 and GSE104942. The data of GSE51032 from the EPIC-Italy cohort were created by Human Genetics Foundation in Italy, including 233 BC patients and 340 cancer-free controls to screen methylation markers correlating with BC risk. The GSE104942 dataset with 87 BC patients and 123 healthy controls was conducted to explore the association between genome-wide gene methylation in PBLs and BC susceptibility. Among the two GEO datasets, the samples in GSE51032 dataset were collected before the onset of BC. Genome-wide methylation of GSE51032 and GSE104942 was assessed using the Infinium HumanMethylation450K platform. The methylation level of each CpG site was represented by the ratio between methylated probe intensities and total intensities (beta-value), and the range of that was 0 to 1.

### Data Source of Immune-Related Gene Dataset

Immune-related genes were downloaded from InnateDB database (https://www.innatedb.com) and ImmPort database (https://immport.niaid.nih.gov/home). A total of 6,148 immune-related genes were collected that are involved in various aspects of immune function, such as antigen processing and presentation, cytokines, and the regulation of T-cell and B-cell signal transduction pathway.

### Data Preprocessing and DMC Selection

Firstly, the CpG sites with missing values greater than 10% in the total sample were removed and all missing data were imputed by the k-nearest neighbor imputation method. Secondly, probes were filtered based on the following conditions using the “chAMP” package of Bioconductor: (1) probes with <3 beads in at least 5% of samples examined; (2) probes with a detection *p* > 0.01; (3) non-CpG probes; (4) single-nucleotide polymorphism (SNP)-related probes; (5) multihit probes; and (6) probes located in X and Y chromosome. Beta-mixture quantile normalization (BMIQ) was applied for normalization. Thirdly, all the CpG sites were annotated and combined with the immune-related gene dataset to obtain the CpG sites of immune-related genes. DMCs related to BC were identified using *t*-test with *p*-value less than 0.05 and the absolute value of the average methylation difference between cases and controls (|Δβ|) greater than 0.015. We integrated the immune-related DMCs associated with BC risk from the two GEO datasets and identified the final immune-related DMCs for the following study.

### Participants of the Case-Control Study

A hospital-based case-control study consisting of 418 BC cases and 511 controls was conducted. All the patients of BC identified by pathological diagnosis were enrolled at the Third Affiliated Hospital of Harbin Medical University from 2010 to 2014. The cancer-free controls were collected from the Second Affiliated Hospital of Harbin Medical University and the community of Xiangfang in Harbin during the same period. Peripheral blood (5 ml) of every participant was collected before surgery or before treatment and immediately stored at −80°C.

### Data Collection

Through in-person interviews, all subjects completed a structured questionnaire with information on demographic characteristics, environmental factors, and family history of cancers. We received informed consent from all subjects. All the procedures were in accordance with the ethical standards of the Ethics Committee in Harbin Medical University, as well as the 1964 Declaration of Helsinki and its subsequent revisions.

### DNA Extraction, Bisulfate Conversion, and Leukocyte Sorting

The genomic DNA was extracted using the QIAamp DNA Blood Mini kit (Qiagen, Hilden, Germany). Bisulfite modification of DNA was conducted using the EpiTect Plus DNA Bisulfite Kit (Qiagen). To analyze the difference of methylation levels in different cell subtypes, we sorted the white blood cells from 26 BC cases and 25 controls into T cells, B cells, and monocytes using a commercial kit (Solarbio, Beijing, China). All the operations followed with the manufacturer’s instructions.

### Methylation Sequencing Assay

We detected the DNA methylation levels and methylation haplotype using MethylTatget™ (Genesky Biotechnologies Inc., Shanghai, China), an NGS-based multiple targeted CpG methylation analysis method. The optimized primer sets were used for multiplex PCR. After constructing the library, samples were sequenced on the Illumina MiSeq platform (Illumina MiSeq Benchtop Sequencer, San Diego, CA, USA) with 2 × 150 bp paired-end model according to the manufacturer’s protocol. The methylation levels of the region within 150 bp adjacent to the screened CpG sites were detected. Moreover, repeated samples were set, and correlation analysis was conducted between DNA methylation levels of the initial samples and repeated samples to ensure the accuracy of the experiment. The detail information of primer sequences is shown in **Supplementary Table S1**.

### Quality Control

The procedure of quality control was conducted for the sequencing data. Firstly, samples with the bisulfite conversion rate lower than 95% after sequencing were deleted. Secondly, the CpG sites with ratios of missing value greater than 30% were filtered out. Finally, the samples with sequencing depth of less than 100× were removed.

### Comethylation Analyses

DNA methylation levels at nearby CpG sites may be highly correlated (also known as comethylation) (13). “coMET” package was applied to analyze and visualize comethylation of CpG sites. The correlations are calculated by Spearman’s analysis. When comethylation existed among adjacent CpG sites, weighted methylation level (WML) of the gene region was calculated using the following formula:


$$\text{WML}=\sum_{i=1}^{n}Ci/\sum_{i=1}^{n}Ci+Ti$$

where C is the read supporting methylated cytosine; T the read supporting unmethylated cytosine; i the position of cytosine; n the total number of cytosine positions; $\Sigma_{i=1}^{n}Ci$ the sum of methylated reads at all CpG sites in the targeted region; and $\Sigma_{i=1}^{n}\;Ci+Ti$ the sum of total reads at all CpG sites in the targeted region.

### DNA Methylation Haplotype Analysis

To evaluate the patterns of methylation haplotype, we defined a metric called methylation haplotype burden (MHB), which is weighted by the frequency of methylation in the targeted sequencing region (**Supplementary Figure S1**).


$$MHB=\sum_{i=1}^{n}\left(f_{i}\times P_{i}\right)$$

Where *f_i_* is the frequency of DNA methylation at the CpG sites in the haplotype pattern and *P_i_* is the reads of the haplotype divided by the total number of reads in the targeted region.

### Statistical Analysis

The homogeneity between cases and controls were evaluated using Student’s *t*-test and Chi-square test. ROC curves and cutoff values determined by Youden index were applied for categorizing all participants into hypomethylation group and hypermethylation group. Univariate and multivariate logistic regression analyses were used to estimate the association between the methylation of immune-related genes and BC risk with corresponding odds ratios (OR) and 95% confidence interval (CI). In the logistic regressing analyses, BC status of samples (Yes/No) and the methylation level of genes were considered the dependent and independent variables, respectively. In addition, we adjusted for the variables that were different in the distribution of demographic information between cases and controls in multivariate regression analysis. We performed all the statistical analyses using R version 3.5.1. All the statistical tests were two sided, *p*-values <0.05 were considered statistically significant in the overall analysis, and *p*-values in the subgroup analyses were adjusted by Bonferroni correction.

## Results

### Identification of DMCs

After integrating the annotated CpG sites in the GEO and immune-related gene datasets, 94,700 immune-related CpG sites in the GSE51032 and 103559 in GSE104942 datasets were selected. Based on the criteria of *p* < 0.05 and |Δβ| *>*0.015 in the differential methylation analysis, we preliminarily selected 1,857 DMCs of immune-related genes from the GSE51032 dataset and 3,844 DMCs from the GSE104942 dataset. After taking the intersection of the DMCs in the two GEO datasets, a total of 91 immune-related DMCs were defined in the two GEO datasets, including 85 hypomethylated CpG sites and 6 hypermethylated CpG sites (**Supplementary Table S2** and **Supplementary Figures S2**, **S3**).

### Go Enrichment Analysis

GO enrichment analysis was performed on the 86 genes covered by 91 CpG sites using DAVID software to further understand the function of the DMCs. The results showed that DMCs were mainly associated with 57 Go terms (**Supplementary Figure S4**). The top 5 significantly enriched GO terms of biological process included T-cell receptor signaling pathway, signal transduction, insulin-like growth factor receptor signaling pathway, positive regulation of apoptotic process and antigen processing, and presentation.

We selected the DMCs related to immune regulation mechanism for further study based on the results of the top 5 significant Go terms. Finally, a total of five DMCs that played the roles in T-cell receptor signaling pathway and antigen processing and presentation were determined as the candidate CpG sites, including cg01760846 [Proteasome 26S subunit, ATPase, 1 (*PSMC1*)], cg07141527 (*SPPL3*), cg15658543 (*CARD11*), cg21568368 (*PSMB8*), and cg24045276 (*NCF2*).

### The Association Between the Methylation of the Five CpG Sites and BC Risk in the GSE51032 Dataset

**Table 1** shows the relationship between the methylation of cg01760846, cg07141527, cg24045276, cg15658543, and cg21568368 and BC risk. Hypermethylation of cg01760846 was associated with increased risk of BC (OR_adj_ = 2.262, 95% CI: 1.590–3.218, *p* < 0.001). Moreover, there were significant associations between hypermethylation of cg07141527, cg15658543, cg21568368, and cg24045276 and reduced risk of BC (OR_adj_ = 0.433, 95% CI:0.301–0.625, *p* < 0.001; OR_adj_ = 0.470, 95% CI: 0.333–0.663, *p* < 0.001; OR_adj_ = 0.586, 95% CI: 0.414–0.830, *p* = 0.003; OR_adj_ = 0.513, 95% CI: 0.358–0.736, *p* < 0.001, respectively).

**Table 1:** The association between methylation of five CpG sites and the risk of BC in GSE51032.

CpG Site	GSE51032						GSE104942			
	Case (%)	Control (%)	Univariate Analysis		Multivariate Analysis		Case (%)	Control (%)	Univariate Analysis	
			OR (95% CI)	*p* ^*^	OR (95% CI)^a^	*p* ^*^			OR (95% CI)	*p* ^*^
cg01760846										
Hypo	127 (54.51%)	248 (72.94%)	2.250 (1.583–3.198)	<0.001	2.262 (1.590–3.218)	<0.001	34 (45.33%)	79 (67.52%)	2.507 (1.380–4.554)	0.003
Hyper	106 (45.49%)	92 (27.06%)					41 (54.67%)	38 (32.48%)		
cg07141527										
Hypo	93 (39.91%)	76 (22.35%)	0.433 (0.301–0.625)	<0.001	0.434 (0.301–0.626)	<0.001	65 (86.67%)	77 (65.81%)	0.296 (0.137–0.638)	0.002
Hyper	140 (40.09%)	264 (77.65%)					10 (13.33%)	40 (34.19%)		
cg15658543										
Hypo	153 (65.67%)	161 (47.35%)	0.470 (0.333–0.663)	<0.001	0.470 (0.333–0.663)	<0.001	61 (81.33%)	71 (60.68%)	0.354 (0.178–0.706)	0.003
Hyper	80 (34.33%)	179 (52.65%)					14 (18.67%)	46 (39.32%)		
cg21568368										
Hypo	158 (67.81%)	188 (55.29%)	0.587 (0.414–0.832)	0.003	0.586 (0.414–0.830)	0.003	42 (56.00%)	31 (26.50%)	0.283 (0.153–0.523)	<0.001
Hyper	75 (32.19%)	152 (44.71%)					33 (44.00%)	86 (73.50%)		
cg24045276										
Hypo	92 (39.48%)	86 (25.29%)	0.519 (0.362–0.743)	<0.001	0.513 (0.358–0.736)	<0.001	47 (62.67%)	46 (39.32%)	0.386 (0.212–0.701)	0.002
Hyper	141 (60.52%)	254 (74.71%)					28 (37.33%)	71 (60.68%)		

a ORs adjusted for age.

^*^p-values <0.05 were considered statistically significant.

### Basic Information of Subjects in the Case-Control Study

The basic characteristics of all the participants are shown in **Table 2**. There were significant statistical differences of family history of BC and other cancers between cases and controls. Considering age is the known confounding factor, we adjusted for age and family history of BC and other cancers in the following analysis.

**Table 2:** Demographic characteristics of BC patients and controls in case-control study.

Characteristics^a^	Case (%)	Control (%)	*p* ^*^
No. of participants	418	511	
Age (year) (mean ± SD)	51.93 ± 9.46	52.06 ± 10.60	0.813
≤50	202 (49.03%)	245 (48.80%)	0.075
50–	134 (32.52%)	133 (26.50%)	
60–	63 (15.29%)	103 (20.72%)	
>70	13 (3.16%)	20 (3.98%)	
BMI (kg/m^2^) (mean ± SD)	23.76 ± 3.48	23.85 ± 3.52	0.866
<18.5	12 (2.91%)	22 (4.54%)	0.418
18.5–	221 (53.64%)	244 (50.30%)	
24–	111 (26.94%)	127 (26.19%)	
≥27	68 (16.51%)	92 (18.97%)	
Race			0.236
Han	403 (97.82%)	479 (95.42%)	
Other	9 (2.18%)	23 (4.58%)	
Family history of other cancers			<0.001
No	310 (75.06%)	425 (84.66%)	
Yes	103 (24.94%)	77 (15.34%)	
Family history of breast cancer			<0.001
No	388 (93.95%)	495 (98.61%)	
Yes	25 (6.05%)	7 (1.39%)	

a Missing value of age, 6 cases and 9 controls; BMI, 6 cases and 26 controls; race, 6 cases and 9 controls; family history of BC, 5 cases and 9 controls; family history of other cancers, 5 cases and 9 controls.

SD, standard deviation; BMI, body mass index.

^*^p-values <0.05 were considered statistically significant.

### Quality Control

For the *PSMC1* (cg01760846) region, a total of 25 samples with sequencing depth less than 100× were deleted, including 9 cases and 16 controls. For the *SPPL3* (cg07141527) region, 122 samples were deleted, including 45 cases and 77 controls. For the *CARD11* (cg15658543) region, a total of 54 samples were deleted, including 17 cases and 37 controls. For the *PSMB8* (cg21568368) region, 9 samples were deleted, including 5 cases and 4 controls. For the *NCF2* (cg24045276) region, 8 samples were deleted, including 5 cases and 3 controls (**Supplementary Figure S5**). The basic characteristics of the subjects for each gene are shown in **Supplementary Tables S3–S7**.

### The Association Between Gene Methylation and BC Risk

**Supplementary Table S1** shows the basic information of targeted regions in the five genes. In the targeted regions of *PSMC1*, *SPPL3*, *CARD11*, *PSMB8*, and *NCF2*, the number of the CpG sites detected by MethylTarget sequencing were 14, 6, 14, 6, and 5. Cutoff values of each CpG site is listed in **Supplementary Table S8**.

#### The Region of *PSMC1*

Hypermethylation of 12 CpG sites were associated with increased risk of BC. Among the 12 CpG sites, the genomic position at 90722706 corresponded to the cg01760846 of the Illumina 450K BeadChip in the GEO dataset. We observed a significant association between the methylation of the CpG site and increased BC risk, which is consistent with the result of the GEO dataset (**Table 3** and **Supplementary Table S9**). Comethylation analysis showed that the methylation levels of the 12 CpG sites were highly correlated. Therefore, we calculated the weighted methylation level of *PSMC1* (*PSMC1*_DMR) and found that hypermethylation of *PSMC1*_DMR still correlated with increased BC risk (OR_adj_ = 1.693, 95% CI = 1.286–2.230, *p* < 0.001) (**Table 4**).

**Table 3:** The association between methylation of the CpG sites from five genes and the risk of BC.

CpG_Position	Case (%)	Control (%)	*χ* ^2^	*p* ^*^	Univariate Analysis		Multivariate Analysis	
					OR (95%CI)	*p* ^*^	OR (95%CI)^1^	*p* ^*^
*PSMC1*_90722706								
Hypo	200 (40.40%)	134 (32.68%)	5.741	0.017	1.396 (1.062–1.836)	0.017	1.385 (1.048–1.831)	0.022
Hyper	295 (59.60%)	276 (67.32%)						
*PSMC1*_90722716								
Hypo	289 (58.38%)	205 (50.00%)	6.359	0.012	1.403 (1.078–1.826)	0.012	1.403 (1.071–1.838)	0.014
Hyper	206 (41.62%)	205 (50.00%)						
*PSMC1*_90722782								
Hypo	271 (54.75%)	189 (46.10%)	6.713	0.010	1.415 (1.088–1.840)	0.010	1.466 (1.119–1.921)	0.005
Hyper	224 (45.25%)	221 (53.90%)						
*PSMC1*_90722795								
Hypo	295 (59.60%)	209 (50.98%)	6.753	0.009	1.419 (1.089–1.847)	0.009	1.471 (1.123–1.928)	0.005
Hyper	200 (40.40%)	201 (49.02%)						
*PSMC1*_90722799								
Hypo	226 (45.66%)	165 (40.24%)	2.677	0.102	1.247 (0.957–1.626)	0.102	1.184 (0.903–1.553)	0.222
Hyper	269 (54.34%)	245 (59.76%)						
*PSMC1*_90722830								
Hypo	308 (62.22%)	238 (58.05%)	2.051	0.201	1.190 (0.911–1.555)	0.202	1.168 (0.888–1.535)	0.267
Hyper	187 (37.78%)	172 (41.95%)						
*PSMC1*_90722856								
Hypo	254 (51.31%)	169 (41.22%)	9.178	0.002	1.503 (1.154–1.957)	0.002	1.492 (1.139–1.954)	0.004
Hyper	241 (48.69%)	241 (58.78%)						
*PSMC1*_90722861								
Hypo	376 (75.96%)	283 (69.02%)	5.449	0.020	1.418 (1.057–1.902)	0.02	1.360 (1.007–1.836)	0.045
Hyper	119 (24.04%)	127 (30.98%)						
*PSMC1*_90722870								
Hypo	248 (50.1%)	162 (39.51%)	10.147	0.001	1.537 (1.179–2.004)	0.001	1.534 (1.169–2.013)	0.002
Hyper	247 (49.9%)	248 (60.49%)						
*PSMC1*_90722877								
Hypo	238 (48.08%)	143 (34.88%)	16.037	<0.001	1.729 (1.321–2.263)	<0.001	1.682 (1.278–2.215)	<0.001
Hyper	257 (51.92%)	267 (65.12%)						
*PSMC1*_90722886								
Hypo	217 (43.84%)	139 (33.9%)	9.278	0.002	1.522 (1.1610–1.995)	0.002	1.511 (1.145–1.993)	0.004
Hyper	278 (56.16%)	271 (66.1%)						
*PSMC1*_90722891								
Hypo	96 (19.39%)	53 (12.93%)	6.819	0.009	1.621 (1.126–2.333)	0.009	1.703 (1.168–2.483)	0.006
Hyper	399 (80.61%)	357 (87.07%)						
*PSMC1*_90722911								
Hypo	242 (48.89%)	140 (34.15%)	19.981	<0.001	1.845 (1.409–2.416)	<0.001	1.844 (1.400–2.429)	<0.001
Hyper	253 (51.11%)	270 (65.85%)						
*PSMC1*_90722917								
Hypo	381 (76.97%)	278 (67.8%)	9.516	0.002	1.587 (1.182–2.130)	0.002	1.604 (1.187–2.167)	0.002
Hyper	114 (23.03%)	132 (32.2%)						
*SPPL3*_121202409								
Hypo	101 (23.27%)	64 (17.16%)	4.690	0.030	1.469 (1.036–2.083)	0.031	1.400 (0.979–2.001)	0.065
Hyper	333 (76.73%)	309 (82.84%)						
*SPPL3*_121202464								
Hypo	115 (26.5%)	76 (20.38%)	4.246	0.039	1.414 (1.016–1.966)	0.040	1.359 (0.968–1.908)	0.076
Hyper	319 (73.5%)	297 (79.62%)						
*SPPL3*_121202539								
Hypo	150 (34.56%)	103 (27.61%)	4.606	0.032	1.390 (1.028–1.878)	0.032	1.320 (0.970–1.795)	0.077
Hyper	284 (65.44%)	270 (72.39%)						
*SPPL3*_121202552								
Hypo	158 (36.41%)	172 (46.11%)	7.637	0.006	0.672 (0.507–0.891)	0.006	0.658 (0.493–0.879)	0.005
Hyper	276 (63.59%)	201 (53.89%)						
*SPPL3*_121202554								
Hypo	295 (67.97%)	283 (75.87%)	6.273	0.012	0.673 (0.493–0.918)	0.012	0.660 (0.479–0.909)	0.011
Hyper	139 (32.03%)	90 (24.13%)						
*SPPL3*_121202602								
Hypo	297 (68.43%)	225 (60.32%)	5.639	0.018	1.420 (1.063–1.897)	0.018	1.411 (1.049–1.898)	0.017
Hyper	137 (31.57%)	148 (39.68%)						
*CARD11*_3026478								
Hypo	306 (64.56%)	284 (70.65%)	3.669	0.055	0.757 (0.569–1.007)	0.056	0.747 (0.558–1.000)	0.050
Hyper	168 (35.44%)	118 (29.35%)						
*CARD11*_3026468								
Hypo	378 (79.75%)	338 (84.08%)	2.735	0.098	0.746 (0.526–1.057)	0.099	0.759 (0.532–1.083)	0.128
Hyper	96 (20.25%)	64 (15.92%)						
*CARD11*_3026460								
Hypo	393 (82.91%)	363 (90.30%)	10.040	0.002	0.521 (0.347–0.784)	0.002	0.498 (0.327–0.758)	0.001
Hyper	81 (17.09%)	39 (9.70%)						
*CARD11*_3026436								
Hypo	142 (29.96%)	88 (21.89%)	7.311	0.007	1.526 (1.122–2.075)	0.007	1.510 (1.101–2.072)	0.011
Hyper	332 (70.04%)	314 (78.11%)						
*CARD11*_3026433								
Hypo	161 (33.97%)	111 (27.61%)	4.102	0.043	1.349 (1.009–1.802)	0.043	1.375 (1.022–1.849)	0.035
Hyper	313 (66.03%)	291(72.39%)						
*CARD11*_3026413								
Hypo	172 (36.29%)	166 (41.29%)	2.301	0.129	0.810 (0.616–1.064)	0.130	0.753 (0.568–0.996)	0.050
Hyper	302 (63.71%)	236 (58.71%)						
*CARD11*_3026389								
Hypo	70 (14.77%)	34 (8.46%)	8.278	0.004	1.875 (1.216–2.893)	0.004	1.896 (1.217–2.953)	0.005
Hyper	404 (85.23%)	368 (91.54%)						
*CARD11*_3026380								
Hypo	226 (47.68%)	213 (52.99%)	2.449	0.118	0.809 (0.620–1.055)	0.118	0.806 (0.614–1.058)	0.120
Hyper	248 (52.32%)	189 (47.01%)						
*CARD11*_3026375								
Hypo	124 (26.16%)	81 (20.15%)	4.385	0.036	1.404 (1.021–1.930)	0.037	1.484 (1.070–2.058)	0.018
Hyper	350 (73.84%)	321 (79.85%)						
*CARD11*_3026348								
Hypo	154 (32.49%)	145 (36.07%)	1.240	0.265	0.853 (0.645–1.129)	0.266	0.858 (0.644–1.143)	0.295
Hyper	320 (67.51%)	257 (63.93%)						
*CARD11*_3026326								
Hypo	250 (52.74%)	191 (47.51%)	2.380	0.123	1.233 (0.945–1.609)	0.123	1.248 (0.951–1.638)	0.111
Hyper	224 (47.26%)	211 (52.49%)						
*CARD11*_3026321								
Hypo	70 (14.77%)	39 (9.70%)	5.125	0.024	1.613 (1.063–2.446)	0.025	1.657 (1.082–2.537)	0.020
Hyper	404 (85.23%)	363 (90.30%)						
*CARD11*_3026317								
Hypo	364 (76.79%)	339 (84.33%)	7.793	0.005	0.615 (0.436–0.867)	0.005	0.614 (0.432–0.872)	0.007
Hyper	110 (23.21%)	63 (15.67%)						
*CARD11*_3026310								
Hypo	325 (68.57%)	302 (75.12%)	4.600	0.032	0.722 (0.536–0.973)	0.032	0.749 (0.552–1.017)	0.064
Hyper	149 (31.43%)	100 (24.88%)						
*PSMB8*_32812098								
Hypo	152 (29.98%)	81 (19.57%)	13.081	<0.001	1.760 (1.293–2.396)	<0.001	1.768 (1.289–2.425)	<0.001
Hyper	355 (70.02%)	333 (80.43%)						
*PSMB8*_32812113								
Hypo	223 (43.98%)	147 (35.51%)	6.814	0.009	1.426 (1.092–1.863)	0.009	1.455 (1.106–1.914)	0.007
Hyper	284 (56.02%)	267 (64.49%)						
*PSMB8*_32812165								
Hypo	334 (65.88%)	249 (60.14%)	3.224	0.073	1.279 (0.977–1.674)	0.073	1.228 (0.931–1.618)	0.146
Hyper	173 (34.12%)	165 (39.86%)						
*PSMB8*_32812167								
Hypo	331 (65.29%)	243 (58.70%)	4.215	0.040	1.323 (1.013–1.730)	0.040	1.311 (0.997–1.725)	0.053
Hyper	176 (34.71%)	171 (41.30%)						
*PSMB8*_32812213								
Hypo	345 (68.05%)	255 (61.59%)	4.180	0.041	1.328 (1.011–1.743)	0.041	1.348 (1.020–1.781)	0.036
Hyper	162 (31.95%)	159 (38.41%)						
*PSMB8*_32812221								
Hypo	370 (72.98%)	270 (65.22%)	6.475	0.011	1.440 (1.087–1.909)	0.011	1.469 (1.101–1.959)	0.009
Hyper	137 (27.02%)	144 (34.78%)						
*NCF2*_183551942								
Hypo	163 (32.09%)	164 (39.61%)	5.646	0.017	0.720 (0.549–0.944)	0.018	0.706 (0.533–0.935)	0.015
Hyper	345 (67.91%)	250 (60.39%)						
*NCF2*_183551969								
Hypo	387 (76.18%)	341 (82.37%)	5.254	0.022	0.685 (0.495–0.948)	0.022	0.665 (0.476–0.929)	0.017
Hyper	121 (23.82%)	73 (17.63%)						
*NCF2*_183551986								
Hypo	371 (73.03%)	331 (79.95%)	6.013	0.014	0.679 (0.498–0.926)	0.014	0.667 (0.485–0.917)	0.013
Hyper	137 (26.97%)	83 (20.05%)						
*NCF2*_183552072								
Hypo	359 (70.67%)	318 (76.81%)	4.411	0.036	0.727 (0.540–0.980)	0.036	0.740 (0.545–1.003)	0.052
Hyper	149 (29.33%)	96 (23.19%)						
*NCF2*_183552095								
Hypo	414 (81.5%)	362 (87.44%)	6.046	0.014	0.633 (0.438–0.913)	0.014	0.600 (0.410–0.878)	0.008
Hyper	94 (18.5%)	52 (12.56%)						

CI, confidence interval; OR, odds ratio.

a ORs adjusted for age and family history of other cancers and breast cancer.

^*^p-values <0.05 were considered statistically significant.

**Table 4:** The association between weighted methylation levels of *PSMC1* and *PSMB8* region and BC risk.

Gene	Case (%)	Control (%)	*χ* ^2^	*p* ^*^	Univariate Analysis		Multivariate Analysis	
					OR (95% CI)	*p* ^*^	OR (95% CI)^a^	*p* ^*^
*PSMC1*_DMR								
Hypo	225 (55.01%)	337 (68.08%)	16.262	<0.001	1.744 (1.33–2.288)	<0.001	1.693 (1.286–2.230)	<0.001
Hyper	184 (44.99%)	158 (31.92%)						
*PSMB8*_DMR								
Hypo	162 (39.23%)	251 (49.51%)	9.726	0.002	1.519 (1.167–1.977)	0.002	1.561 (1.194–2.041)	<0.001
Hyper	251 (60.77%)	256 (50.49%)						

CI, confidence interval; OR, odds ratio.

a ORs adjusted for age and family history of other cancers and breast cancer.

^*^p-values <0.05 were considered statistically significant.

#### The Region of *SPPL3*

Multivariate logistic regression analysis showed that hypermethylation of the CpG sites located at 121202552 and 121202554 were associated with reduced BC risk (OR_adj_ = 0.658, 95% CI = 0.493–0.879, *p* = 0.005; OR_adj_ = 0.660, 95% CI = 0.479–0.909, *p* = 0.011). The hypermethylation of the CpG sites located at 121202602 was statistically associated with a higher risk of BC (OR_adj_ = 1.411, 95% CI = 1.049–1.898, *p* = 0.017). In addition, the genomic position at 121202554 corresponded to the cg07141527 in the GEO dataset, and the relationship between this CpG site and BC risk was similar to the result of the GEO dataset (**Table 3** and **Supplementary Table S9**).

#### The Region of *CARD11*

Among the 14 CpG sites, hypermethylation of 7 CpG sites were related to BC risk. Hypermethylation of 5 CpG sites located at 3026436, 3026433, 3026389, 3026375, and 3026321 were associated with higher risk of BC. Hypermethylation of the other two CpG sites located at 3026460 and 3026317 had the relationship with reduced BC risk. The CpG site that located at 3026310 corresponded to the cg15658543 in the GEO dataset. We did not observe the significant association between the methylation of the CpG site and BC risk (**Table 3** and **Supplementary Table S9**).

#### The Region of *PSMB8*

We found that hypermethylation of 4 CpG sites were associated with increased risk of BC. Moreover, comethylation existed among the 4 CpG sites (**Table 3**). After calculating the WML of *PSMB8* (*PSMB8*_DMR), we observed that there was significant association between hypermethylation of *PSMB8*_DMR and increased risk of BC (OR_adj_ = 1.561, 95% CI = 1.194–2.041, *p* < 0.001) (**Table 4**). However, we did not find the methylation of the CpG site in the case-control study, that corresponded to the cg21568368 in the GEO dataset was associated with BC risk (**Table 3** and **Supplementary Table S9**).

#### The Region of *NCF2*

The results showed that hypermethylation of 4 CpG sites located at 183551942, 183551969, 183551986, and 183552095 were related to decreased risk of BC, with the ORs of 0.706 (95% CI: 0.533–0.935, *p* = 0.015), 0.665 (95% CI: 0.476–0.929, *p* = 0.017), 0.667 (95% CI: 0.485–0.917, *p* = 0.013), and 0.600 (95% CI: 0.410–0.878, *p* = 0.008), respectively. The CpG site located at 183552095 corresponded to the cg24045276 of the Illumina 450K BeadChip and correlated with lower risk of BC, which is consistent with the result of the GEO dataset (**Table 3** and **Supplementary Table S9**).

### Subgroup Analysis

We carried out subgroup analyses based on age (≤60 and >60 years), molecular types (Luminal A, Luminal B, Her-2, base-like), and ER status (ER positive, ER negative). In the subgroup analysis stratified by age, we observed significant association between hypermethylation of *PSMC1*_DMR and *PSMB8*_DMR and increased BC risk in young subjects (≤60 years). Hypermethylation of *CARD11* was associated with BC risk in the subjects ≤60 years. The patients >60 years with the hypermethylation of *NCF2* were related to reduced BC risk (**Supplementary Table S10**). Moreover, stratified by BC molecular types and ER status, significant association between the methylation of the five genes and BC risk in different subgroups were observed. The detail results are shown in **Supplementary Tables S11–S13**.

### DNA Methylation Haplotype and BC Risk

A total of 83 methylation haplotypes were detected in the five genes, including 22 haplotypes in the *PSMC1* region, 20 haplotypes in the *SPPL3* region, 24 haplotypes in the *CARD11* region, 7 haplotypes in the *PSMB8* region, and 10 haplotypes in the *NCF2* region. The haplotypes in the region of each gene were quantified by MHB. After adjusting for age and family history of BC and other cancers, MHBs of *PSMC1*, *CARD11*, and *PSMB8* were found to be associated with reduced BC risk (OR_adj_ = 0.587, 95% CI: 0.438–0.785, *p* < 0.001; OR_adj_ = 0.584, 95% CI: 0.377–0.905, *p* = 0.016; OR_adj_ = 0.640, 95% CI: 0.485–0.843, *p* = 0.002, respectively) (**Table 5**).

**Table 5:** The association between methylation haplotype burden of five genes and BC risk.

Cpg Sites	Cases (%)	Controls (%)	Univariate Analysis		Multivariate Analysis	
			OR (95% CI)	*p* ^*^	OR (95% CI)^a^	*p* ^*^
*PSMC1*_Haplotype						
Low	149 (36.40%)	123 (24.85%)	0.577 (0.433–0.768)	<0.001	0.587 (0.438–0.785)	<0.001
High	260 (63.60%)	372 (75.15%)				
*SPPL3*_Haplotype						
Low	137 (36.73%)	186 (42.86%)	1.292 (0.973–1.716)	0.077	1.255 (0.941–1.674)	0.122
High	236 (63.27%)	248 (57.14%)				
*CARD11*_Haplotype						
Low	364 (90.77%)	407 (85.86%)	0.617 (0.403–0.945)	0.026	0.584 (0.377–0.905)	0.016
High	37 (9.22%)	67 (14.14%)				
*PSMB8*_Haplotype						
Low	169 (40.92%)	157 (30.97%)	0.648 (0.493–0.850)	0.002	0.640 (0.485–0.843)	0.002
High	244 (59.08%)	350 (69.03%)				
*NCF2*_Haplotype						
Low	255 (61.74%)	290 (57.08%)	0.824 (0.632–1.074)	0.153	0.810 (0.618–1.061)	0.127
High	158 (38.26%)	218 (42.92%)				

CI, confidence interval; OR, odds ratio.

a ORs adjusted for age and family history of other cancers and breast cancer.

^*^p-values <0.05 were considered statistically significant.

### Gene Methylation in Different Cell Types

A total of 212 cells were successfully isolated from 53 subjects, including 53 B cells, 53 monocytes, 53 T cells, and 53 residual cells. MethylTarget sequencing was performed on the methylation levels of *PSMC1*, *SPPL3*, and *CARD11* in the selected cells. We compared the differences of methylation levels in cell subtypes. The results showed that *PSMC1*_DMR methylation levels were different among the three cell subtypes in the control group. We also observed different distributions of methylation levels in the *SPPL3* and *CARD11* regions among the different cell subtypes (**Supplementary Figures S6–S8**).

Moreover, we observed that the distribution of *PSMC1*_DMR, *SPPL3*, and *CARD11* methylation in B cells was different between cases and controls. In the T-cell group, there was a statistical difference in the methylation level of CpG site in the SPPL3 region between cases and controls. We observed significant association between methylation of CpG sites in the *SPPL3* region (*SPPL3*_121202552: OR = 13.235, 95% CI = 1.535–114.296, *p* = 0.019; *SPPL3*_121202554: OR = 4.250, 95% CI = 1.322–13.562, *p* = 0.015) and *CARD11* region (*CARD11*_3026375: OR = 4.714, 95% CI = 1.266–17.561, *p* = 0.021; *CARD11*_3026321:OR = 4.792, 95% CI = 1.136–20.21, *p* = 0.033; *CARD11*_3026317:OR = 6.353, 95% CI = 1.216–33.191, *p* = 0.028) in B cells and BC risk. In the T-cell group, there was a significant association between the CpG site in the *SPPL3* region and BC risk (*SPPL3*_121202552: OR = 11.000, 95% CI = 2.902–41.690, *p* < 0.001).

## Discussion

In our study, we firstly screened the DMCs of immune-related genes that related to BC in PBLs from the GEO public database. A total of five immune-related genes were screened for further study. Secondly, we conducted a case-control study to further validate the significant correlation between the methylation of the genes and BC risk. We observed significant associations between the methylation of the genes and BC risk. Moreover, the differences in the methylation levels of five immune-related genes in T cells, B cells, and monocytes were analyzed by sorting PBLs. There were differences in the methylation levels of three genes in different cell subtypes. Finally, MHB was used to explore the relationship between DNA methylation haplotype pattern and BC risk. We found statistical differences between methylation haplotypes of *PSMC1*, *CARD11*, and *PSMB8* and BC risk.

Serving as a member of the PSMC family, *PSMC1* is located in the base region of the proteasome 19S regulatory particle and participates in the formation of the 19S regulatory complex (14). The 19S regulatory complex is essential for regulating the 26S proteasome, which is a multicatalytic proteinase complex and critical for depredating various oncoproteins, transcription factors, and other regulatory cellular proteins (15). Studies revealed that *PSMC1* plays an important role in adaptive immunity and antigen processing and is related to the occurrence, development, and prognosis of tumors. Christopher et al. detected the expression of *PSMC1* in 8 BC tissues and normal breast tissues and found that *PSMC1* was significantly overexpressed in BC patients (16). Tzu-Jen Kao et al. explored the role of six members of the PSMC family in BC. The results showed that *PSMC1*, *PSMC2*, *PSMC3*, and *PSMC4* were highly expressed in triple-negative BC. In addition, high levels of *PSMC1*, *PSMC3*, *PSMC4*, *PSMC5*, and *PSMC6* transcripts were associated with poor prognosis in BC patients (17). To now, there was no study to discover the relationship between methylation of *PSMC1* and BC risk. Our research observed that methylation of *PSMC1* was related to BC risk, which can provide more information about PSMC1 gene and cancers from the perspective of epigenetic changes.

In addition to *PSMC1*, *PSMB8* (also known as *LMP7*) is a member of proteasome B-type family, which is a 20S core beta subunit. *PSMB8* is located in the class II region of the MHC and encoded *LMP7* protein. As the member of immunoproteasome, *LMP7* participated in the antigen presentation, cell cycle regulation, and cell signal transduction (18–20). At present, many studies show that abnormal expression of *PSMB8* correlates with multiple cancers, including BC (21–25). However, the results obtained from different studies are not the same. There are few studies on the relationship between *PSMB8* methylation and BC. The results in our research found that hypermethylation of *PSMB8* was associated with BC risk. We hypothesized that *PSMB8* hypermethylation may result in abnormal expression of *PSMB8*, which can affect the processing of class I MHC peptides and antigen processing and cause cancers.

Moreover, we observed a significant association between the methylation of *CARD11*, *SPPL3*, and *NCF2* and BC risk. *CARD11* (known as *CARMA1*) is responsible for encoding a member of proteins that belongs to the membrane-associated guanylate kinase (MAGUK) family. *CARD11* plays an important role in adaptive immunity, particularly in the activation of NF-κB pathway mediated by TCR (26, 27). *SPPL3* belonging to the family of GxGD intramembrane proteases is important for a variety of immune system functions (28–31). Moreover, NADPH oxidase (NOX family protein) is composed of six subunits of gp91phox, p22phox, p47phox, p67phox, p40phox, and Rac2. Studies showed that most reactive oxygen species (ROS), which are crucial for regulating immune function and cell differentiation, are produced by the activation of NADPH oxidase (32). *NCF2* (known as P67phox), a subunit of NADPH oxidase, plays an important role in regulating cell growth, differentiation, and immune function (33). Studies suggested that genetic mutation and abnormal expression of the three genes contribute to cancers (33–40). Our study identified the methylation of the three genes were associated with BC risk in the GEO dataset and validated these results in the case-control study. Our study firstly revealed the relationship between the methylation of the three genes and BC risk in PBLs that can further demonstrate that three-gene methylation may play vital roles in the development of BC and are expected to be new biomarkers for BC.

In our research, the DMCs sequenced by MethylTarget sequencing technology were mainly located in the CpG island shore (2 kb regions flanking CpG island) and openseas (>4 kb to the nearest CpG island) (41, 42). Although, most studies have indicated that functionally important DNA methylation will occur in promoters, and that most DNA methylation changes in cancer occur in CpG islands (42). Studies revealed that methylation alterations in cancers also can occur in CpG island shore, and the methylation in CpG island shore was strongly related to gene expression. Moreover, CpG sites located in openseas may encompass distal genomic regulatory elements and were associated with enhancing or repressing transcription (43). Methylation of CpGs in the shore and openseas also play an essential role in the development of cancers. Our study further suggested that methylation of CpG sites located in the CpG shore and openseas were associated with BC risk. However, the relationship between abnormal methylation of CpGs in different locations and gene expression remains to be further studied.

It has been revealed that adjacent CpG sites on the same DNA molecules tend to possess similar methylation statuses, due to the processivity of the DNA methylation and/or demethylation enzymes. However, inconsistent DNA methylation pattern has been observed, that is methylation haplotype (13, 44). Studies suggested that DNA methylation haplotype signatures are present in several cancer types and associated with cancers. Normally, studies on DNA methylation markers are based on a single-CpG methylation level or the average methylation levels, which cannot reflect the DNA methylation load of patients. In our study, DNA methylation haplotype load was defined to quantify the DNA methylation haplotype pattern in the gene region. Our results showed that MHBs of PSMC1, CARD11, and PSMB8 were associated with reduced BC risk, which indicated the role of MHBs in BC risk.

Recent research suggested that DNA methylation is heterogeneous among different cell subtypes. The study of Reinius showed that there were differences in the methylation profiles in whole blood, mononuclear cells, and granulocytes. After analyzing the 8,252 probes of 343 genes implicated in immune-related disorders from genome-wide association study, they observed 22% difference of CpG methylation between mononuclear cells and granulocytes (45). In the case-control study, it also has been found that there were heterogeneities in the methylation levels of the five genes among different cell subtypes by sorting PBLs, which can illustrate that the methylation levels are affected by the proportion of cells. Since the cell sorting was performed in a small number of the samples in the case-control study, we did not adjust the cell proportion in the analysis. We calculated the proportion of six subtype cells (B cells, T cells, NK cells, CD4+T cells, CD8+T cells, monocytes, and neutrophils) using “EpiDISH” package in the GSE51032 dataset and adjusted for the proportion of six subtype cells when we analyzed the association between methylation of five screened CpG sites and BC risk. We still observed significance between methylation of the five CpG sites and BC risk (**Supplementary Table S14**), which suggested that the association between methylation and BC risk is still significant under the influence of the cell heterogeneity.

There are still some limits in our research. Firstly, a hospital-based case-control study may give rise to bias such as recall bias, Neyman bias, and Berkson’s bias. In our study, all the interviewees were trained uniformly before collecting information, and the quality control of questionnaire was carried out by special personnel. Secondly, case-control study cannot provide the temporal order of a causal relation between methylation statuses and BC. However, the data in the GSE51032 dataset came from a nested case-control study and all the samples in the GSE51032 dataset were collected before the onset of BC, which can directly confirm the temporal relationship between methylation changes and tumorigenesis. Our results about the association between methylation of five genes and BC risk are consistent with the GEO dataset. Moreover, studies showed that the difference of overall methylation level in peripheral blood between cases and controls is small and we also observed a small difference of methylation levels of immune-related CpG sites between BC cases and controls in public datasets. However, the |Δβ| threshold set as 0.015 for DMC screening is still very small regardless of the significant *p*-value. Moreover, the small difference of overall methylation level cannot represent the difference of methylation for an individual site. The above viewpoint suggests that our research needs to be cautious when drawing conclusions, and it is necessary to validate the difference of methylation level for an individual CpG site in peripheral blood and their association with BC risk in other public databases or other study. In addition, the sample sizes in the subgroup analysis and the process of sorting peripheral blood leukocytes are relatively small, which may limit the statistical power in our research. More studies involving larger samples may be needed to improve the statistical power.

In conclusion, our results suggested that methylation of immune-related genes in PBLs were associated with BC risk. The five gene methylation could be the potential biomarkers for predicting BC risk.

## Data Availability Statement

The data presented in the study are deposited in the Figshare repository. Dataset: https://doi.org/10.6084/m9.figshare.17450303.v1.

## Ethics Statement

The studies involving human participants were reviewed and approved by The Ethics Committee in Harbin Medical University. The patients/participants provided their written informed consent to participate in this study.

## Author Contributions

YZ and DP designed the study, directed its implementation, including quality assurance and control, and reviewed the manuscript. JF, DL, XZ, and YL put forward and helped the analytic strategy of the study. TT, JF, and YL did the data analysis and wrote the manuscript. TT, JF, XW, DZ, and TZ performed the experimental work and contributed to the sample collection. All the authors approved the submission of this manuscript. The work described has not been submitted elsewhere for publication, in whole or in part. All the authors have reviewed the final version of the manuscript and approved it for publication.

## Funding

This study was funded by the Natural Science Foundation of China (Grant No. 81172743) and Special fund for the construction of High-level Universities and Characteristic Disciplines in Heilongjiang Province.

## Conflict of Interest

The authors declare that the research was conducted in the absence of any commercial or financial relationships that could be construed as a potential conflict of interest.

## Publisher’s Note

All claims expressed in this article are solely those of the authors and do not necessarily represent those of their affiliated organizations, or those of the publisher, the editors and the reviewers. Any product that may be evaluated in this article, or claim that may be made by its manufacturer, is not guaranteed or endorsed by the publisher.

## References

[1] Sung H Ferlay J Siegel RL Laversanne M Soerjomataram I Jemal A. Global Cancer Statistics 2020: GLOBOCAN Estimates of Incidence and Mortality Worldwide for 36 Cancers in 185 Countries. CA Cancer J Clin (2021) 71(3):209–49. doi: 10.3322/caac.21660 33538338

[2] Zheng RS Sun KX Zhang SW Zeng HM Zou XN Chen R. Report of Cancer Epidemiology in China 2015. Zhonghua Zhong Liu Za Zhi (2019) 41(1):19–28. doi: 10.3760/cma.j.issn.0253-3766.2019.01.005 30678413

[3] Anderson RL. Understanding Breast Cancer Risk. Radiol Technol (2010) 81(5):457m–76m. 20445140

[4] Lo PK Sukumar S. Epigenomics and Breast Cancer. Pharmacogenomics (2008) 9(12):1879–902. doi: 10.2217/14622416.9.12.1879 PMC2633440 19072646

[5] Waddington CH. The Epigenotype. 1942. Int J Epidemiol (2012) 41(1):10–3. doi: 10.1093/ije/dyr184 22186258

[6] Kulis M Esteller M. DNA Methylation and Cancer. Adv Genet (2010) 70:27–56. doi: 10.1016/b978-0-12-380866-0.60002-2 20920744

[7] Paz MF Fraga MF Avila S Guo M Pollan M Herman JG. A Systematic Profile of DNA Methylation in Human Cancer Cell Lines. Cancer Res (2003) 63(5):1114–21. 12615730

[8] Dunn GP Bruce AT Ikeda H Old LJ Schreiber RD. Cancer Immunoediting: From Immunosurveillance to Tumor Escape. Nat Immunol (2002) 3(11):991–8. doi: 10.1038/ni1102-991 12407406

[9] Singh R Paterson Y. Immunoediting Sculpts Tumor Epitopes During Immunotherapy. Cancer Res (2007) 67(5):1887–92. doi: 10.1158/0008-5472.Can-06-3960 17332314

[10] Emens LA. Breast Cancer Immunobiology Driving Immunotherapy: Vaccines and Immune Checkpoint Blockade. Expert Rev Anticancer Ther (2012) 12(12):1597–611. doi: 10.1586/era.12.147 PMC3587160 23253225

[11] Cimino-Mathews A Foote JB Emens LA. Immune Targeting in Breast Cancer. Oncol (Williston Park) (2015) 29(5):375–85. 25979549

[12] Fitzpatrick DR Wilson CB. Methylation and Demethylation in the Regulation of Genes, Cells, and Responses in the Immune System. Clin Immunol (2003) 109(1):37–45. doi: 10.1016/s1521-6616(03)00205-5 14585274

[13] Shoemaker R Deng J Wang W Zhang K. Allele-Specific Methylation Is Prevalent and is Contributed by CpG-SNPs in the Human Genome. Genome Res (2010) 20(7):883–9. doi: 10.1101/gr.104695.109 PMC2892089 20418490

[14] Hoyle J Fisher EM. Genomic Organization and Mapping of the Mouse P26s4 ATPase Gene: A Member of the Remarkably Conserved AAA Gene Family. Genomics (1996) 31(1):115–8. doi: 10.1006/geno.1996.0017 8808288

[15] Kloetzel PM. The Proteasome and MHC Class I Antigen Processing. Biochim Biophys Acta (2004) 1695(1-3):225–33. doi: 10.1016/j.bbamcr.2004.10.004 15571818

[16] Barnes CJ Li F Talukder AH Kumar R. Growth Factor Regulation of a 26S Proteasomal Subunit in Breast Cancer. Clin Cancer Res (2005) 11(8):2868–74. doi: 10.1158/1078-0432.Ccr-04-1989 15837734

[17] Kao TJ Wu CC Phan NN Liu YH Ta HDK Anuraga G. Prognoses and Genomic Analyses of Proteasome 26S Subunit, ATPase (PSMC) Family Genes in Clinical Breast Cancer. Aging (Albany NY) (2021) 13(14):17970. doi: 10.18632/aging.203345 34329194 PMC8351721

[18] Gileadi U Moins-Teisserenc HT Correa I Booth BL Jr Dunbar PR Sewell AK. Generation of an Immunodominant CTL Epitope is Affected by Proteasome Subunit Composition and Stability of the Antigenic Protein. J Immunol (1999) 163(11):6045–52. 10570292

[19] Basler M Mundt S Groettrup M. The Immunoproteasome Subunit LMP7 is Required in the Murine Thymus for Filling Up a Hole in the T Cell Repertoire. Eur J Immunol (2018) 48(3):419–29. doi: 10.1002/eji.201747282 29067678

[20] Zhang X Li F Wang W Ji L Sun B Xiao X. Macrophage Pyroptosis Is Mediated by Immunoproteasome Subunit β5i (LMP7) in Abdominal Aortic Aneurysm. Biochem Biophys Res Commun (2020) 533(4):1012–20. doi: 10.1016/j.bbrc.2020.09.082 33019975

[21] Cabrera CM Jiménez P Cabrera T Esparza C Ruiz-Cabello F Garrido F. Total Loss of MHC Class I in Colorectal Tumors can be Explained by Two Molecular Pathways: Beta2-Microglobulin Inactivation in MSI-Positive Tumors and LMP7/TAP2 Downregulation in MSI-Negative Tumors. Tissue Antigens (2003) 61(3):211–9. doi: 10.1034/j.1399-0039.2003.00020.x 12694570

[22] Miller Z Ao L Kim KB Lee W. Inhibitors of the Immunoproteasome: Current Status and Future Directions. Curr Pharm Des (2013) 19(22):4140–51. doi: 10.2174/1381612811319220018 PMC3821965 23181576

[23] Kaur G Batra S. Emerging Role of Immunoproteasomes in Pathophysiology. Immunol Cell Biol (2016) 94(9):812–20. doi: 10.1038/icb.2016.50 PMC13360420 27192937

[24] Lee M Song IH Heo SH Kim YA Park IA Bang WS. Expression of Immunoproteasome Subunit LMP7 in Breast Cancer and Its Association With Immune-Related Markers. Cancer Res Treat (2019) 51(1):80–9. doi: 10.4143/crt.2017.500 PMC6333994 29510614

[25] Chen D Jin C Dong X Wen J Xia E Wang Q. Pan-Cancer Analysis of the Prognostic and Immunological Role of PSMB8. Sci Rep (2021) 11(1):20492. doi: 10.1038/s41598-021-99724-9 34650125 PMC8516870

[26] Thome M Charton JE Pelzer C Hailfinger S. Antigen Receptor Signaling to NF-kappaB *via* CARMA1, BCL10, and MALT1. Cold Spring Harb Perspect Biol (2010) 2(9):a003004. doi: 10.1101/cshperspect.a003004 20685844 PMC2926749

[27] Bedsaul JR Carter NM Deibel KE Hutcherson SM Jones TA Wang Z. Mechanisms of Regulated and Dysregulated CARD11 Signaling in Adaptive Immunity and Disease. Front Immunol (2018) 9:2105. doi: 10.3389/fimmu.2018.02105 30283447 PMC6156143

[28] Voss M Schröder B Fluhrer R. Mechanism, Specificity, and Physiology of Signal Peptide Peptidase (SPP) and SPP-Like Proteases. Biochim Biophys Acta (2013) 1828(12):2828–39. doi: 10.1016/j.bbamem.2013.03.033 24099004

[29] Makowski SL Wang Z Pomerantz JL. A Protease-Independent Function for SPPL3 in NFAT Activation. Mol Cell Biol (2015) 35(2):451–67. doi: 10.1128/mcb.01124-14 PMC4272424 25384971

[30] Hamblet CE Makowski SL Tritapoe JM Pomerantz JL. NK Cell Maturation and Cytotoxicity Are Controlled by the Intramembrane Aspartyl Protease Sppl3. J Immunol (2016) 196(6):2614–26. doi: 10.4049/jimmunol.1501970 PMC4779696 26851218

[31] Jongsma MLM de Waard AA Raaben M Zhang T Cabukusta B Platzer R. The SPPL3-Defined Glycosphingolipid Repertoire Orchestrates HLA Class I-Mediated Immune Responses. Immunity (2021) 54(1):132–50.e139. doi: 10.1016/j.immuni.2020.11.003 33271119 PMC8722104

[32] Segal BH Grimm MJ Khan AN Han W Blackwell TS. Regulation of Innate Immunity by NADPH Oxidase. Free Radic Biol Med (2012) 53(1):72–80. doi: 10.1016/j.freeradbiomed.2012.04.022 22583699 PMC3377837

[33] Juhasz A Ge Y Markel S Chiu A Matsumoto L van Balgooy J. Expression of NADPH Oxidase Homologues and Accessory Genes in Human Cancer Cell Lines, Tumours and Adjacent Normal Tissues. Free Radic Res (2009) 43(6):523–32. doi: 10.1080/10715760902918683 PMC2843555 19431059

[34] Lee JY Park AK Lee KM Park SK Han S Han W. Candidate Gene Approach Evaluates Association Between Innate Immunity Genes and Breast Cancer Risk in Korean Women. Carcinogenesis (2009) 30(9):1528–31. doi: 10.1093/carcin/bgp084 19372141

[35] Noh JM Kim J Cho DY Choi DH Park W Huh SJ. Exome Sequencing in a Breast Cancer Family Without BRCA Mutation. Radiat Oncol J (2015) 33(2):149–54. doi: 10.3857/roj.2015.33.2.149 PMC4493427 26157685

[36] Ostalecki C Lee JH Dindorf J Collenburg L Schierer S Simon B. Multiepitope Tissue Analysis Reveals SPPL3-Mediated ADAM10 Activation as a Key Step in the Transformation of Melanocytes. Sci Signal (2017) 10(470). doi: 10.1126/scisignal.aai8288 28292959

[37] Maltby VE Lea RA Graves MC Sanders KA Benton MC Tajouri L. Genome-Wide DNA Methylation Changes in CD19(+) B Cells From Relapsing-Remitting Multiple Sclerosis Patients. Sci Rep (2018) 8(1):17418. doi: 10.1038/s41598-018-35603-0 30479356 PMC6258668

[38] Qi F Qin WX Zang YS. Molecular Mechanism of Triple-Negative Breast Cancer-Associated BRCA1 and the Identification of Signaling Pathways. Oncol Lett (2019) 17(3):2905–14. doi: 10.3892/ol.2019.9884 PMC6365902 30854067

[39] Qin K Zheng Z He Y Gao Y Shi H Mo S. High Expression of Neutrophil Cytosolic Factor 2 (NCF2) Is Associated With Aggressive Features and Poor Prognosis of Esophageal Squamous Cell Carcinoma. Int J Clin Exp Pathol (2020) 13(12):3033–43. PMC7791380 33425104

[40] McGuire MH Dasari SK Yao H Wen Y Mangala LS Bayraktar E. Gene Body Methylation of the Lymphocyte-Specific Gene CARD11 Results in Its Overexpression and Regulates Cancer mTOR Signaling. Mol Cancer Res (2021) 19(11):1917–28. doi: 10.1158/1541-7786.Mcr-20-0753 PMC8568653 34348992

[41] Irizarry RA Ladd-Acosta C Wen B Wu Z Montano C Onyango P. The Human Colon Cancer Methylome Shows Similar Hypo- and Hypermethylation at Conserved Tissue-Specific CpG Island Shores. Nat Genet (2009) 41(2):178–86. doi: 10.1038/ng.298 PMC2729128 19151715

[42] Sandoval J Heyn H Moran S Serra-Musach J Pujana MA Bibikova M. Validation of a DNA Methylation Microarray for 450,000 CpG Sites in the Human Genome. Epigenetics (2011) 6(6):692–702. doi: 10.4161/epi.6.6.16196 21593595

[43] Malta TM de Souza CF Sabedot TS Silva TC Mosella MS Kalkanis SN. Glioma CpG Island Methylator Phenotype (G-CIMP): Biological and Clinical Implications. Neuro Oncol (2018) 20(5):608–20. doi: 10.1093/neuonc/nox183 PMC5892155 29036500

[44] Jones B. DNA Methylation: Switching Phenotypes With Epialleles. Nat Rev Genet (2014) 15(9):572. doi: 10.1038/nrg3797 25069489

[45] Reinius LE Acevedo N Joerink M Pershagen G Dahlén SE Greco D. Differential DNA Methylation in Purified Human Blood Cells: Implications for Cell Lineage and Studies on Disease Susceptibility. PloS One (2012) 7(7):e41361. doi: 10.1371/journal.pone.0041361 22848472 PMC3405143

