# Supplementary material for: Methylation of Immune-Related Genes in Peripheral Blood Leukocytes and Breast Cancer
Source: Front Oncol. 2022 Feb 10;12:817565. doi: 10.3389/fonc.2022.817565 (PMC8867609; doi:10.3389/fonc.2022.817565)
Supplement: Supplementary file 1 [file DataSheet_1.docx]

**Table S1. Primer sequences for the five genes.**

| **Gene** | **mRNA** | **Primer Sequence** |  | **Product information** | | | | | |
| --- | --- | --- | --- | --- | --- | --- | --- | --- | --- |
|  |  |  | **chromosomal location** | **Genomic Location** | **Product size (bp)** | **Strand** | **CpG** | **Relation_to_Island** | **UCSC_RefGene_Group** |
| *PSMC1* | NM_002802 | F- GGGGTTATTGTTTTAAGTTGTTGATAAG | Chr14 | 90722678-90722946 | 269 | + | 14 | N_Shore | TSS200 |
|  |  | R- ACCATCTTACCTTAACCACTTAAACTACC |  |  |  |  |  |  |  |
| *SPPL3* | NM_139015 | F- GTTTTAAAGTTTTYGAGGGGTTATTG | Chr12 | 121202374-121202629 | 256 | + | 6 | OpenSea | 3'UTR |
|  |  | R- ACCCTATTTAATTCCTTCTCTTCCTAC |  |  |  |  |  |  |  |
| *CARD11* | NM_032415 | F- AGGGGTTAGGTAGGGAGTGAA | Chr7 | 3026507-3026269 | 239 | - | 14 | OpenSea | 5'UTR |
|  |  | R- CAACCCCTACTCACAACCTCAA |  |  |  |  |  |  |  |
| *PSMB8* | NM_148919 | F- TGGAAATAGGGGTGGGTAGG | Chr6 | 32812076-32812252 | 177 | + | 6 | S_Shore | 1stExon |
|  |  | R- CCAAAACCTATTTCCAAAACTCTACC |  |  |  |  |  |  |  |
| *NCF2* | NM_000433 | F- AAGATTTATAAGGTATAGGATTTGAAAGATAG | Chr1 | 183551907-183552128 | 222 | + | 8 | OpenSea | Body |
|  |  | R- CCCCCTCTATTTCCCTCTATTTTC |  |  |  |  |  |  |  |

**Table S2. Differential methylation analysis of 91 CpG cites in GEO datasets.**

|  |  |  |  | **GSE51032** |  |  | **GSE104942** |  |  |
| --- | --- | --- | --- | --- | --- | --- | --- | --- | --- |
| **Gene** | **CpG site** | **Chr** | **Position** | **t** | **Δβ** | ***P* value*** | **t** | **Δβ** | ***P* value*** |
| *A2ML1* | cg23546356 | 12 | 8974786 | 2.361 | -0.015 | 0.019 | 2.577 | -0.020 | 0.011 |
| *ABCC4* | cg11744861 | 13 | 95920671 | 2.464 | -0.020 | 0.014 | 2.289 | -0.029 | 0.023 |
| *ABR* | cg26682335 | 17 | 945834 | 5.010 | -0.024 | <0.001 | 3.518 | -0.025 | 0.001 |
| *ADAMDEC1* | cg01759110 | 8 | 24241694 | -3.055 | 0.015 | 0.002 | -2.282 | 0.018 | 0.024 |
| *AGGF1* | cg15817406 | 5 | 76325554 | 5.393 | -0.020 | <0.001 | 3.398 | -0.021 | 0.001 |
| *APITD1* | cg07166784 | 1 | 10508703 | 4.484 | -0.033 | <0.001 | 1.983 | -0.017 | 0.049 |
| *ARL4A* | cg06415087 | 7 | 12726571 | 4.023 | -0.015 | <0.001 | 3.167 | -0.017 | 0.002 |
| *ASCC3* | cg05757007 | 6 | 101053555 | 4.035 | -0.029 | <0.001 | 2.881 | -0.023 | 0.004 |
| *ASGR2* | cg04281898 | 17 | 7011140 | 4.450 | -0.030 | <0.001 | 2.407 | -0.022 | 0.017 |
| *ATM* | cg19892525 | 11 | 108157498 | 7.491 | -0.041 | <0.001 | 2.357 | -0.019 | 0.020 |
| *BCL11A* | cg03754165 | 2 | 60780427 | -3.512 | 0.037 | <0.001 | -2.292 | 0.022 | 0.023 |
| *BRD8* | cg12977827 | 5 | 137512548 | 3.464 | -0.016 | 0.001 | 3.321 | -0.023 | 0.001 |
| *BTN3A1* | cg22068371 | 6 | 26407505 | 2.421 | -0.016 | 0.016 | 2.370 | -0.018 | 0.019 |
| *C1QTNF9* | cg20138486 | 13 | 24884072 | 3.312 | -0.020 | 0.001 | 2.536 | -0.015 | 0.012 |
| *C2* | cg27297326 | 6 | 31894990 | 3.283 | -0.029 | 0.001 | 2.129 | -0.021 | 0.035 |
| *CARD11* | cg15658543 | 7 | 3026309 | 4.704 | -0.019 | <0.001 | 2.948 | -0.020 | 0.004 |
| *CCL13* | cg01566155 | 17 | 32685450 | 3.255 | -0.021 | 0.001 | 2.243 | -0.018 | 0.026 |
| *CHST11* | cg04737991 | 12 | 105050768 | 2.684 | -0.028 | 0.008 | 2.886 | -0.038 | 0.004 |
| *CRIM1* | cg15371815 | 2 | 36582144 | 2.451 | -0.018 | 0.015 | 2.939 | -0.029 | 0.004 |
| *DCBLD1* | cg05053978 | 6 | 117869098 | 2.339 | -0.016 | 0.020 | 2.313 | -0.030 | 0.022 |
| *EBF3* | cg09746817 | 10 | 131671265 | 3.494 | -0.029 | 0.001 | 2.620 | -0.021 | 0.010 |
| *ESRRB* | cg14853964 | 14 | 76870538 | 3.904 | -0.018 | <0.001 | 3.144 | -0.017 | 0.002 |
| *ESRRG* | cg00903998 | 1 | 216897293 | -6.245 | 0.027 | <0.001 | -3.007 | 0.015 | 0.003 |
| *FCHO2* | cg17829914 | 5 | 72385895 | 3.619 | -0.018 | <0.001 | 3.091 | -0.018 | 0.002 |
| *FNDC3B* | cg26074575 | 3 | 171764199 | 3.255 | -0.021 | 0.001 | 3.103 | -0.025 | 0.002 |
| *GGCX* | cg07143583 | 2 | 85776941 | 2.571 | -0.019 | 0.010 | 2.546 | -0.019 | 0.012 |
| *GLT25D1* | cg19414763 | 19 | 17687657 | 3.502 | -0.025 | 0.001 | 2.209 | -0.021 | 0.029 |
| *GNE* | cg05844977 | 9 | 36277400 | 5.789 | -0.035 | <0.001 | 2.293 | -0.016 | 0.023 |
| *GP6* | cg20651389 | 19 | 55549842 | 3.770 | -0.017 | <0.001 | 3.097 | -0.024 | 0.002 |
| *GSTP1* | cg06841499 | 11 | 67349641 | 2.106 | -0.017 | 0.036 | 3.401 | -0.034 | 0.001 |
| *HBXIP* | cg25032124 | 1 | 110946480 | 6.719 | -0.050 | <0.001 | 2.040 | -0.017 | 0.043 |
| *HCG22* | cg14119792 | 6 | 31021422 | 2.614 | -0.018 | 0.009 | 2.671 | -0.026 | 0.008 |
| *HFE2* | cg14599576 | 1 | 145413157 | 3.630 | -0.035 | <0.001 | 2.133 | -0.020 | 0.034 |
| *HLA-DMA* | cg14180039 | 6 | 32919700 | 4.112 | -0.023 | <0.001 | 2.121 | -0.021 | 0.035 |
| *HLA-DPB2* | cg23376071 | 6 | 33087989 | 2.838 | -0.019 | 0.005 | 3.084 | -0.029 | 0.002 |
| *HOXA5* | cg02646423 | 7 | 27183794 | 3.880 | -0.025 | <0.001 | 2.023 | -0.020 | 0.045 |
| *HRH4* | cg26442509 | 18 | 22039496 | 2.945 | -0.016 | 0.003 | 2.978 | -0.018 | 0.003 |
| *ICA1* | cg20022118 | 7 | 8276021 | 4.692 | -0.024 | <0.001 | 2.600 | -0.015 | 0.010 |
| *IFIH1* | cg03538095 | 2 | 163139716 | 4.872 | -0.027 | <0.001 | 2.736 | -0.021 | 0.007 |
| *IGF1R* | cg05110803 | 15 | 99385323 | 2.156 | -0.018 | 0.032 | 2.651 | -0.025 | 0.009 |
| *IGF2R* | cg01188509 | 6 | 160509295 | 4.781 | -0.026 | <0.001 | 2.986 | -0.017 | 0.003 |
| *IGSF5* | cg24698780 | 21 | 41173950 | 3.472 | -0.022 | 0.001 | 2.154 | -0.016 | 0.033 |
| *IRAK1* | cg06334238 | X | 153284899 | -2.736 | 0.018 | 0.006 | -2.044 | 0.044 | 0.042 |
| *ITFG1* | cg05128386 | 16 | 47409367 | 4.172 | -0.026 | <0.001 | 3.157 | -0.021 | 0.002 |
| *ITGAD* | cg03087622 | 16 | 31403683 | 2.999 | -0.018 | 0.003 | 2.073 | -0.015 | 0.040 |
| *KRT1* | cg02425372 | 12 | 53074023 | 6.052 | -0.027 | <0.001 | 2.964 | -0.021 | 0.003 |
| *LAMA2* | cg27120934 | 6 | 129480619 | 4.269 | -0.016 | <0.001 | 3.211 | -0.023 | 0.002 |
| *LEFTY2* | cg27179622 | 1 | 226127290 | 2.519 | -0.021 | 0.012 | 2.013 | -0.028 | 0.046 |
| *LGMN* | cg21196628 | 14 | 93175350 | 4.271 | -0.017 | <0.001 | 2.437 | -0.016 | 0.016 |
| *MCF2L* | cg08890338 | 13 | 113700818 | 5.830 | -0.026 | <0.001 | 2.523 | -0.018 | 0.013 |
| *MID1* | cg06448666 | X | 10645625 | 4.936 | -0.025 | <0.001 | 2.392 | -0.017 | 0.018 |
| *MIR372* | cg22462000 | 19 | 54290434 | 5.836 | -0.026 | <0.001 | 3.081 | -0.017 | 0.002 |
| *MOG* | cg08725892 | 6 | 29629986 | 2.170 | -0.019 | 0.030 | 3.053 | -0.033 | 0.003 |
| *MUC1* | cg23256951 | 1 | 155163205 | 3.623 | -0.017 | <0.001 | 2.327 | -0.017 | 0.021 |
| *NCF2* | cg24045276 | 1 | 183552095 | 3.146 | -0.025 | 0.002 | 2.403 | -0.017 | 0.017 |
| *NET1* | cg01010839 | 10 | 5488225 | 2.737 | -0.023 | 0.006 | 2.841 | -0.031 | 0.005 |
| *NFATC2* | cg01334549 | 20 | 50137002 | 5.233 | -0.018 | <0.001 | 2.516 | -0.015 | 0.013 |
| *NOV* | cg05020510 | 8 | 120428057 | 3.565 | -0.021 | <0.001 | 3.602 | -0.040 | 0.000 |
| *PRMT1* | cg24127106 | 19 | 50183046 | 3.747 | -0.018 | <0.001 | 2.694 | -0.021 | 0.008 |
| *PSMA1* | cg00417576 | 11 | 14535610 | 4.060 | -0.020 | <0.001 | 3.030 | -0.016 | 0.003 |
| *PSMB8* | cg21568368 | 6 | 32812167 | 2.662 | -0.015 | 0.008 | 2.860 | -0.022 | 0.005 |
| *PSMC1* | cg01760846 | 14 | 90722706 | -2.676 | 0.019 | 0.008 | -2.045 | 0.016 | 0.043 |
| *PTPRJ* | cg19268720 | 11 | 48038449 | 2.072 | -0.016 | 0.039 | 3.123 | -0.024 | 0.002 |
| *PTPRS* | cg00614413 | 19 | 5286594 | 3.840 | -0.016 | <0.001 | 2.865 | -0.016 | 0.005 |
| *RAB11FIP4* | cg15331096 | 17 | 29861932 | 2.813 | -0.016 | 0.005 | 3.223 | -0.017 | 0.002 |
| *RHD* | cg10117171 | 1 | 25599238 | 4.750 | -0.039 | <0.001 | 2.113 | -0.022 | 0.036 |
| *RIN3* | cg16255729 | 14 | 93106394 | 2.435 | -0.016 | 0.015 | 3.089 | -0.022 | 0.002 |
| *RNF216* | cg02543993 | 7 | 5736195 | 3.383 | -0.015 | 0.001 | 2.186 | -0.018 | 0.030 |
| *RPS6KA2* | cg18495191 | 6 | 167200499 | -2.802 | 0.018 | 0.005 | -2.747 | 0.022 | 0.007 |
| *SCRN1* | cg10591077 | 7 | 30030138 | 4.064 | -0.029 | <0.001 | 2.727 | -0.020 | 0.007 |
| *SMAGP* | cg10993701 | 12 | 51660035 | 2.385 | -0.016 | 0.017 | 2.706 | -0.017 | 0.008 |
| *SMARCA4* | cg08151828 | 19 | 11093828 | 5.120 | -0.017 | <0.001 | 3.574 | -0.019 | 0.000 |
| *SORL1* | cg10746778 | 11 | 121460778 | 3.683 | -0.033 | <0.001 | 2.493 | -0.019 | 0.014 |
| *SPPL3* | cg07141527 | 12 | 121202554 | 3.635 | -0.030 | <0.001 | 2.553 | -0.018 | 0.012 |
| *SYTL1* | cg12748890 | 1 | 27676205 | 2.534 | -0.015 | 0.012 | 2.618 | -0.032 | 0.010 |
| *TAP2* | cg00167916 | 6 | 32795877 | 4.100 | -0.019 | <0.001 | 3.754 | -0.018 | 0.000 |
| *TCF7L2* | cg22795218 | 10 | 114765022 | 4.952 | -0.030 | <0.001 | 3.029 | -0.020 | 0.003 |
| *TLX1* | cg20120208 | 10 | 102896869 | 3.057 | -0.023 | 0.002 | 2.620 | -0.028 | 0.010 |
| *TNFAIP8* | cg17008454 | 5 | 118724372 | 6.550 | -0.050 | <0.001 | 2.919 | -0.022 | 0.004 |
| *TNXB* | cg19647111 | 6 | 32073035 | 5.068 | -0.029 | <0.001 | 2.683 | -0.016 | 0.008 |
| *TPX2* | cg26558420 | 20 | 30331315 | 4.163 | -0.024 | <0.001 | 2.282 | -0.016 | 0.024 |
| *UNC5B* | cg12873350 | 10 | 72976676 | 2.893 | -0.026 | 0.004 | 2.277 | -0.020 | 0.024 |
| *VHL* | cg15036157 | 3 | 10192045 | 3.819 | -0.025 | <0.001 | 2.301 | -0.020 | 0.023 |
| *VPS45* | cg05228338 | 1 | 150048339 | 3.663 | -0.021 | <0.001 | 1.997 | -0.018 | 0.048 |
| *WASF2* | cg05002165 | 1 | 27813172 | 2.600 | -0.016 | 0.010 | 2.418 | -0.018 | 0.017 |
| *WISP2* | cg04193901 | 20 | 43343736 | 3.908 | -0.015 | <0.001 | 3.116 | -0.021 | 0.002 |
|  | cg06491405 | 6 | 31895257 | 3.495 | -0.023 | 0.001 | 2.080 | -0.015 | 0.039 |
|  | cg12128839 | 7 | 27183436 | 2.682 | -0.024 | 0.008 | 2.184 | -0.033 | 0.030 |
|  | cg03368099 | 7 | 27184521 | 2.394 | -0.017 | 0.017 | 2.417 | -0.026 | 0.017 |
|  | cg05076221 | 7 | 27182637 | 2.285 | -0.016 | 0.023 | 2.197 | -0.024 | 0.029 |
|  | cg25307665 | 7 | 27183694 | 2.035 | -0.020 | 0.042 | 2.286 | -0.039 | 0.024 |

**P* values < 0.05 were considered statistically significant.

**Table S3. Demographic characteristics of participants for *PSMC1* sequencing.**

| **Characteristics** | **Case (%)** | **Control (%)** | ***P**** |
| --- | --- | --- | --- |
| No. of participants | 409 | 495 |  |
| Age (year) (Mean±SD) | 51.24±9.45 | 50.99±10.56 | 0.340 |
| ≤50 | 196 (48.64%) | 242 (49.79%) | 0.075 |
| 50- | 132 (32.75%) | 126 (25.93%) |  |
| 60- | 62 (15.38%) | 99 (20.37%) |  |
| >70 | 13 (3.23%) | 19 (3.91%) |  |
| BMI (kg/m^2^) (Mean±SD) | 23.78±3.49 | 23.87±3.89 | 0.817 |
| <18.5 | 10 (2.48%) | 20 (4.25%) | 0.327 |
| 18.5- | 218 (54.09%) | 239 (50.74%) |  |
| 24- | 109 (27.05%) | 122 (25.90%) |  |
| ≥27 | 66(16.38%) | 90 (19.11%) |  |
| Race |  |  |  |
| Han | 394 (97.77%) | 463 (95.27%) | 0.250 |
| Other | 9 (2.23%) | 23 (4.73%) |  |
| Family history of other cancer |  |  | <0.001 |
| No | 304 (75.25%) | 411 (84.57%) |  |
| Yes | 100 (24.75%) | 75 (15.43%) |  |
| Family history of breast cancer |  |  |  |
| No | 379 (93.81%) | 479 (98.56%) | <0.001 |
| Yes | 25 (6.19%) | 7 (1.45%) |  |

Missing value of age: 6 cases and 9 controls; BMI: 6 cases and 24 controls; Race: 6 cases and 9 controls. Family history of BC: 5 cases and 9 controls; Family history of other tumors: 5 cases and 9 controls.

SD, standard deviation; BMI, body mass index

**P* values < 0.05 were considered statistically significant.

**Table S4. Demographic characteristics of participants for *SPPL3* sequencing.**

| **Characteristics** | **Case (%)** | **Control (%)** | ***P**** |
| --- | --- | --- | --- |
| No. of participants | 373 | 434 |  |
| Age (year) (Mean±SD) | 51.89±9.34 | 52.67±10.62 | 0.279 |
| ≤50 | 179 (48.77%) | 197 (46.46%) | 0.098 |
| 50- | 121 (32.97%) | 120 (28.30%) |  |
| 60- | 56 (15.26%) | 88 (20.76%) |  |
| >70 | 11 (3.00%) | 19 (4.48%) |  |
| BMI (kg/m^2^) (Mean±SD) | 23.74±3.50 | 23.85±3.47 | 0.690 |
| <18.5 | 10 (2.72%) | 19 (46.46%) | 0.311 |
| 18.5- | 202 (54.89%) | 203 (28.30%) |  |
| 24- | 94 (25.54%) | 109 (20.76%) |  |
| ≥27 | 62(16.85%) | 78 (4.48%) |  |
| Race |  |  |  |
| Han | 357 (97.28%) | 406 (95.53%) | 0.192 |
| Other | 10 (2.72%) | 19 (4.47%) |  |
| Family history of other cancer |  |  | 0.001 |
| No | 274 (74.46%) | 356 (83.76%) |  |
| Yes | 94 (25.54%) | 69(16.24%) |  |
| Family history of breast cancer |  |  |  |
| No | 345 (93.75%) | 418 (98.35%) | 0.001 |
| Yes | 23 (6.25%) | 7 (1.65%) |  |

Missing value of age: 6 cases and 9 controls; BMI: 5 cases and 15 controls; Race: 6 cases and 9 controls. Family history of BC: 5 cases and 9 controls; Family history of other tumors: 5 cases and 9 controls.

SD, standard deviation; BMI, body mass index

**P* values < 0.05 were considered statistically significant.

**Table S5. Demographic characteristics of participants for *CARD11* sequencing.**

| **Characteristics** | **Case (%)** | **Control (%)** | ***P**** |
| --- | --- | --- | --- |
| No. of participants | 401 | 474 |  |
| Age (year) (Mean±SD) | 51.98±9.43 | 52.37±10.54 | 0.569 |
| ≤50 | 193 (48.86%) | 219 (47.10%) | 0.083 |
| 50- | 129 (32.66%) | 128 (27.52%) |  |
| 60- | 60 (15.19%) | 99 (21.29%) |  |
| >70 | 13 (3.29%) | 19 (4.09%) |  |
| BMI (kg/m^2^) (Mean±SD) | 23.75±3.52 | 23.84±3.48 | 0.695 |
| <18.5 | 12(3.04%) | 19 (4.23%) | 0.564 |
| 18.5- | 210 (53.16%) | 227 (50.56%) |  |
| 24- | 107(27.09%) | 116 (25.83%) |  |
| ≥27 | 66 (16.71%) | 87 (19.38%) |  |
| Race |  |  |  |
| Han | 386 (97.72%) | 444 (95.48%) | 0.075 |
| Other | 9 (2.28%) | 21 (4.52%) |  |
| Family history of other cancer |  |  | 0.002 |
| No | 299 (75.51%) | 391 (84.09%) |  |
| Yes | 97 (24.49%) | 74 (15.91%) |  |
| Family history of breast cancer |  |  |  |
| No | 372 (93.94%) | 458 (98.49%) | <0.001 |
| Yes | 24 (6.06%) | 7 (1.51%) |  |

Missing value of age: 6 cases and 9 controls; BMI: 5 cases and 25 controls; Race: 6 cases and 9 controls. Family history of BC: 5 cases and 9 controls; Family history of other tumors: 5 cases and 9 controls.

SD, standard deviation; BMI, body mass index

**P* values < 0.05 were considered statistically significant.

**Table S6. Demographic characteristics of participants for *PSMB8* sequencing.**

| **Characteristics** | **Case (%)** | **Control (%)** | ***P**** |
| --- | --- | --- | --- |
| No. of participants | 413 | 507 |  |
| Age (year) (Mean±SD) | 51.90±9.49 | 50.09±10.61 | 0.776 |
| ≤50 | 201 (49.39%) | 243 (48.79%) | 0.072 |
| 50- | 131 (32.19%) | 131 (26.31%) |  |
| 60- | 62 (15.23%) | 104 (20.88%) |  |
| >70 | 13 (3.19%) | 20 (4.02%) |  |
| BMI (kg/m^2^) (Mean±SD) | 23.77±3.49 | 23.78±3.51 | 0.968 |
| <18.5 | 12 (2.95%) | 22 (4.57%) | 0.431 |
| 18.5- | 218 (53.56%) | 243 (50.52%) |  |
| 24- | 110 (27.03%) | 125 (25.99%) |  |
| ≥27 | 67 (16.46%) | 91 (18.92%) |  |
| Race |  |  |  |
| Han | 398 (97.79%) | 475 (95.38%) | 0.051 |
| Other | 9 (2.21%) | 23 (4.62%) |  |
| Family history of other cancer |  |  | <0.001 |
| No | 305 (74.75%) | 422 (84.74%) |  |
| Yes | 103 (25.25%) | 76 (15.26%) |  |
| Family history of breast cancer |  |  |  |
| No | 383 (93.87%) | 491 (98.59%) | <0.001 |
| Yes | 25 (6.13%) | 7 (1.41%) |  |

Missing value of age: 6 cases and 9 controls; BMI: 5 cases and 26 controls; Race: 6 cases and 9 controls. Family history of BC: 5 cases and 9 controls; Family history of other tumors: 5 cases and 9 controls.

SD, standard deviation; BMI, body mass index

**P* values < 0.05 were considered statistically significant.

**Table S7. Demographic characteristics of participants for *NCF2* sequencing.**

| **Characteristics** | **Case (%)** | **Control (%)** | ***P**** |
| --- | --- | --- | --- |
| No. of participants | 413 | 508 |  |
| Age (year) (Mean±SD) | 51.95±9.49 | 52.06±10.62 | 0.872 |
| ≤50 | 201 (49.39%) | 243 (48.80%) | 0.084 |
| 50- | 131 (32.19%) | 131 (26.30%) |  |
| 60- | 62 (15.23%) | 104 (20.88%) |  |
| >70 | 13 (3.19%) | 20 (4.02%) |  |
| BMI (kg/m^2^) (Mean±SD) | 23.75±3.50 | 23.78±3.51 | 0.895 |
| <18.5 | 12 (2.95%) | 22 (4.56%) | 0.400 |
| 18.5- | 219 (53.81%) | 243 (50.42%) |  |
| 24- | 110 (27.03%) | 126 (26.14%) |  |
| ≥27 | 66 (16.21%) | 91 (18.88%) |  |
| Race |  |  |  |
| Han | 398 (97.79%) | 475 (95.38%) | 0.051 |
| Other | 9 (2.21%) | 23 (4.62%) |  |
| Family history of other cancer |  |  | <0.001 |
| No | 306 (75.00%) | 423 (84.77%) |  |
| Yes | 102 (25.00%) | 76 (15.23%) |  |
| Family history of breast cancer |  |  |  |
| No | 383 (93.86%) | 492 (98.60%) | <0.001 |
| Yes | 25 (6.14%) | 7 (1.40%) |  |

Missing value of age: 6 cases and 9 controls; BMI: 5 cases and 26 controls; Race: 6 cases and 9 controls. Family history of BC: 5 cases and 9 controls; Family history of other tumors: 5 cases and 9 controls.

SD, standard deviation; BMI, body mass index

**P* values < 0.05 were considered statistically significant.

**Table S8. Detail information of cut-off value of each detected CpG sites.**

| **CpG sites** | **AUC** | **z statistic** | ***P* value** | **Youden** | **Cut-off value** |
| --- | --- | --- | --- | --- | --- |
| PSMC1_90722706 | 0.53363 | 1.752 | 0.0797 | 0.08041 | 0.0059 |
| PSMC1_90722716 | 0.53137 | 1.620 | 0.1052 | 0.08788 | 0.0054 |
| PSMC1_90722782 | 0.54284 | 2.228 | 0.0259 | 0.09618 | 0.0072 |
| PSMC1_90722795 | 0.53797 | 1.982 | 0.0475 | 0.08864 | 0.0103 |
| PSMC1_90722799 | 0.50958 | 0.496 | 0.6200 | 0.059 | 0.0063 |
| PSMC1_90722830 | 0.51494 | 0.775 | 0.4385 | 0.0478 | 0.0080 |
| PSMC1_90722856 | 0.55134 | 2.690 | 0.0071 | 0.1071 | 0.0066 |
| PSMC1_90722861 | 0.5431 | 2.245 | 0.0248 | 0.07743 | 0.0081 |
| PSMC1_90722870 | 0.55326 | 2.785 | 0.0054 | 0.1116 | 0.0050 |
| PSMC1_90722877 | 0.57382 | 3.897 | 0.0001 | 0.1345 | 0.0052 |
| PSMC1_90722886 | 0.55262 | 2.751 | 0.0059 | 0.1022 | 0.0043 |
| PSMC1_90722891 | 0.52975 | 1.548 | 0.1217 | 0.06585 | 0.0038 |
| PSMC1_90722911 | 0.57181 | 3.780 | 0.0002 | 0.1478 | 0.0041 |
| PSMC1_90722917 | 0.54833 | 2.516 | 0.0119 | 0.09367 | 0.0083 |
| SPPL3_121202409 | 0.50009 | 0.00439 | 0.9965 | 0.0639 | 0.9525 |
| SPPL3_121202464 | 0.52103 | 1.034 | 0.301 | 0.06177 | 0.9667 |
| SPPL3_121202539 | 0.51969 | 0.966 | 0.3339 | 0.07253 | 0.9057 |
| SPPL3_121202552 | 0.5223 | 1.094 | 0.2741 | 0.09584 | 0.9793 |
| SPPL3_121202554 | 0.52188 | 1.078 | 0.2812 | 0.08231 | 0.9847 |
| SPPL3_121202602 | 0.5279 | 1.371 | 0.1705 | 0.08199 | 0.9748 |
| CARD11_3026478 | 0.52166 | 1.110 | 0.2671 | 0.06836 | 0.9762 |
| CARD11_3026468 | 0.51345 | 0.688 | 0.4913 | 0.04582 | 0.9662 |
| CARD11_3026460 | 0.5144 | 0.738 | 0.4605 | 0.07885 | 0.9898 |
| CARD11_3026436 | 0.51174 | 0.601 | 0.5479 | 0.08489 | 0.9718 |
| CARD11_3026433 | 0.50909 | 0.464 | 0.6425 | 0.06354 | 0.9554 |
| CARD11_3026413 | 0.5109 | 0.556 | 0.5782 | 0.05218 | 0.9722 |
| CARD11_3026389 | 0.51037 | 0.531 | 0.5952 | 0.06521 | 0.9617 |
| CARD11_3026380 | 0.51569 | 0.801 | 0.4231 | 0.05592 | 0.9747 |
| CARD11_3026375 | 0.50582 | 0.298 | 0.7659 | 0.06011 | 0.9681 |
| CARD11_3026348 | 0.50247 | 0.126 | 0.900 | 0.04002 | 0.9727 |
| CARD11_3026326 | 0.51242 | 0.635 | 0.5253 | 0.0523 | 0.9792 |
| CARD11_3026321 | 0.50958 | 0.491 | 0.6235 | 0.05066 | 0.9660 |
| CARD11_3026317 | 0.52074 | 1.064 | 0.2871 | 0.07784 | 0.9823 |
| CARD11_3026310 | 0.51945 | 0.997 | 0.3189 | 0.06559 | 0.9713 |
| PSMB8_32812098 | 0.56146 | 3.254 | 0.0011 | 0.1101 | 0.0047 |
| PSMB8_32812113 | 0.54663 | 2.447 | 0.0144 | 0.09419 | 0.0062 |
| PSMB8_32812165 | 0.52268 | 1.186 | 0.2358 | 0.05733 | 0.0072 |
| PSMB8_32812167 | 0.53151 | 1.648 | 0.0993 | 0.06788 | 0.0063 |
| PSMB8_32812213 | 0.52664 | 1.393 | 0.1636 | 0.06848 | 0.0069 |
| PSMB8_32812221 | 0.52358 | 1.228 | 0.2193 | 0.08047 | 0.0080 |
| NCF2_183551942 | 0.518 | 0.939 | 0.3478 | 0.07768 | 0.8450 |
| NCF2_183551969 | 0.51891 | 0.994 | 0.3202 | 0.06911 | 0.9384 |
| NCF2_183551986 | 0.52924 | 1.535 | 0.1247 | 0.07162 | 0.8956 |
| NCF2_183552072 | 0.52307 | 1.210 | 0.2261 | 0.06384 | 0.9293 |
| NCF2_183552095 | 0.50844 | 0.444 | 0.6569 | 0.06427 | 0.8763 |
| PSMC_DMR | 0.57836 | 4.13 | <0.0001 | 0.134 | 0.0069 |
| PSMB8_DMR | 0.55406 | 2.839 | 0.0045 | 0.1134 | 0.0059 |
| PSMC1_Haplot | 0.55821 | 3.041 | 0.0024 | 0.1158 | 0.8153 |
| SPPL 3_Haplot | 0.53098 | 1.520 | 0.1286 | 0.06396 | 0.6438 |
| CARD11_Haplot | 0.50896 | 0.457 | 0.6477 | 0.04908 | 0.5410 |
| PSMB8_Haplot | 0.54475 | 2.344 | 0.0191 | 0.09954 | 0.9141 |
| NCF2_Haplot | 0.51226 | 0.643 | 0.5202 | 0.04899 | 0.3841 |

**Table S9. Comparison the results from GEO datasets and case-control study.**

|  |  | **GSE51032** | | | | **GSE104942** | | | | **Case-control study** | | | |
| --- | --- | --- | --- | --- | --- | --- | --- | --- | --- | --- | --- | --- | --- |
| **CpG site** |  | **Case (%)** | **Control (%)** | **OR (95% CI) ^1^** | ***P**** | **Case (%)** | **Control (%)** | **OR (95% CI)** | ***P**** | **Case (%)** | **Control (%)** | **OR (95% CI)^2^** | ***P**** |
| cg01760846 |  |  |  |  |  |  |  |  |  |  |  |  |  |
|  | hypo | 127 (54.51%) | 248 (72.94%) | 2.262 (1.590-3.218) | <0.001 | 34 (45.33%) | 79 (67.52%) | 2.507 (1.380-4.554) | 0.003 | 200 (40.40%) | 134 (32.68%) | 1.385 (1.048-1.831) | 0.022 |
|  | hyper | 106 (45.49%) | 92 (27.06%) |  |  | 41 (54.67%) | 38 (32.48%) |  |  | 295 (59.60%) | 276 (67.32%) |  |  |
| cg07141527 |  |  |  |  |  |  |  |  |  |  |  |  |  |
|  | hypo | 93 (39.91%) | 76 (22.35%) | 0.434 (0.301-0.626) | <0.001 | 65 (86.67%) | 77 (65.81%) | 0.296 (0.137-0.638) | 0.002 | 295 (67.97%) | 283 (75.87%) | 0.660 (0.479-0.909) | 0.011 |
|  | hyper | 140 (40.09%) | 264 (77.65%) |  |  | 10(13.33%) | 40 (34.19%) |  |  | 139 (32.03%) | 90 (24.13%) |  |  |
| cg15658543 |  |  |  |  |  |  |  |  |  |  |  |  |  |
|  | hypo | 153 (65.67%) | 161 (47.35%) | 0.470 (0.333-0.663) | <0.001 | 61 (81.33%) | 71 (60.68%) | 0.354 (0.178-0.706) | 0.003 | 325 (68.57%) | 302 (75.12%) | 0.749 (0.552-1.017) | 0.064 |
|  | hyper | 80 (34.33%) | 179 (52.65%) |  |  | 14 (18.67%) | 46 (39.32%) |  |  | 149 (31.43%) | 100 (24.88%) |  |  |
| cg21568368 |  |  |  |  |  |  |  |  |  |  |  |  |  |
|  | hypo | 158 (67.81%) | 188 (55.29%) | 0.586 (0.414-0.830) | 0.003 | 42 (56.00%) | 31 (26.50%) | 0.283 (0.153-0.523) | <0.001 | 331 (65.29%) | 243 (58.70%) | 1.311 (0.997-1.725) | 0.053 |
|  | hyper | 75 (32.19%) | 152 (44.71%) |  |  | 33 (44.00%) | 86 (73.50%) |  |  | 176 (34.71%) | 171 (41.30%) |  |  |
| cg24045276 |  |  |  |  |  |  |  |  |  |  |  |  |  |
|  | hypo | 92 (39.48%) | 86 (25.29%) | 0.513 (0.358-0.736) | <0.001 | 47 (62.67%) | 46 (39.32%) | 0.386 (0.212-0.701) | 0.002 | 414 (81.5%) | 362 (87.44%) | 0.600 (0.410-0.878) | 0.008 |
|  | hyper | 141 (60.52%) | 254 (74.71%) |  |  | 28 (37.33%) | 71 (60.68%) |  |  | 94 (18.5%) | 52 (12.56%) |  |  |

^1^ ORs adjusted for age. ^2^ ORs adjusted for age, family history of other cancers and breast cancer. **P* values < 0.05 were considered statistically significant

**Table S10. The association between methylation levels of the CpG sites in five genes and the risk of breast cancer in the subgroup analysis of age.**

|  |  | **≤60 years** | | | | | | **>60 years** | | | | | | |
| --- | --- | --- | --- | --- | --- | --- | --- | --- | --- | --- | --- | --- | --- | --- |
| **CpG sites*^1^*** | |  | | **Univariate analysis** | | **Multivariate analysis** | |  | | **Univariate analysis** | | **Multivariate analysis** | | |
|  |  | **Cases (%)** | **Controls (%)** | **OR (95%CI)** | ***P**** | **OR (95%CI)^1^** | ***P**** | **Cases (%)** | **Controls (%)** | **OR (95%CI)** | ***P**** | | **OR (95%CI)^1^** | ***P**** |
| *PSMC1_DMR* | hypo | 179(54.57%) | 262(71.20%) |  |  |  |  | 43(57.33%) | 70(59.32%) |  |  | |  |  |
|  | hyper | 149(45.43%) | 106(28.80%) | 2.057(1.504-2.814) | <0.001 | 1.975(1.438-2.712) | <0.001 | 32(42.67%) | 48(40.68%) | 1.085(0.604-1.951) | 0.785 | | 1.122(0.614-2.048) | 0.708 |
| *SPPL3_121202552* | hypo | 116(36.59%) | 144(48.00%) |  |  |  |  | 26(37.14%) | 36(33.33%) |  |  | |  |  |
|  | hyper | 201(63.41%) | 156(52.00%) | 0.625(0.453-0.863) | 0.004 | 0.683(0.460-1.014) | 0.058 | 44(62.86%) | 72(66.67%) | 0.846(0.451-1.586) | 0.271 | | 0.880(0.457-1.697) | 0.703 |
| *SPPL3_121202554* | hypo | 219(69.09%) | 230(76.67%) |  |  |  |  | 52(74.29%) | 70(64.81%) |  |  | |  |  |
|  | hyper | 98(30.91%) | 70(23.33%) | 0.680(0.475-0.973) | 0.035 | 0.690(0.465-1.024) | 0.065 | 18(25.71%) | 38(35.19%) | 0.638(0.328-1.241) | 0.185 | | 0.611(0.301-1.241) | 0.173 |
| *SPPL3_121202602* | hypo | 219(69.09%) | 183(61.00%) |  |  |  |  | 40(57.14%) | 69(63.89%) |  |  | |  |  |
|  | hyper | 98(30.91%) | 117(39.00%) | 1.429(1.025-1.992) | 0.035 | 1.424(1.017-1.995) | 0.040 | 30(42.86%) | 39(36.11%) | 1.327(0.717-2.454) | 0.367 | | 1.290(0.678-2.456) | 0.297 |
| *CARD11_3026460* | hypo | 294(91.30%) | 289(83.29%) |  |  |  |  | 63(86.30%) | 96(81.36%) |  |  | |  |  |
|  | hyper | 28(8.70%) | 58(16.71%) | 0.475(0.294-0.766) | 0.002 | 0.459(0.281-0.749) | 0.002 | 10(13.70%) | 22(18.64%) | 0.693(0.307-1.56) | 0.376 | | 0.684(0.298-1.570) | 0.370 |
| *CARD11_3026436* | hypo | 70(21.74%) | 105(30.26%) |  |  |  |  | 15(20.55%) | 31(26.27%) |  |  | |  |  |
|  | hyper | 252(78.26%) | 242(69.74%) | 1.562(1.101-2.217) | 0.013 | 1.579(1.108-2.251) | 0.012 | 58(79.45%) | 87(73.73%) | 1.378(0.684-2.775) | 0.370 | | 1.408(0.684-2.897) | 0.353 |
| *CARD11_3026433* | hypo | 86(26.71%) | 130(37.46%) |  |  |  |  | 23(31.51%) | 29(24.58%) |  |  | |  |  |
|  | hyper | 236(73.29%) | 217(62.54%) | 1.644(1.183-2.284) | 0.003 | 1.658(1.188-2.313) | 0.003 | 50(68.49%) | 89(75.42%) | 0.708(0.371-1.354) | 0.297 | | 0.710(0.366-1.377) | 0.311 |
| *CARD11_3026389* | hypo | 26(8.07%) | 55(15.85%) |  |  |  |  | 8(10.96%) | 13(11.02%) |  |  | |  |  |
|  | hyper | 296(91.93%) | 292(84.15%) | 2.144(1.309-3.513) | 0.002 | 2.166(1.314-3.572) | 0.002 | 65(89.04%) | 105(88.98%) | 1.006(0.396-2.558) | 0.990 | | 1.148(0.438-3.008) | 0.778 |
| *CARD11_3026375* | hypo | 61(18.94%) | 88(25.36%) |  |  |  |  | 18(24.66%) | 36(30.51%) |  |  | |  |  |
|  | hyper | 261(81.06%) | 259(74.64%) | 1.454(1.005-2.103) | 0.047 | 1.468(1.009-2.135) | 0.045 | 55(75.34%) | 82(69.49%) | 1.341(0.693-2.598) | 0.384 | | 1.526(0.768-3.034) | 0.228 |
| *CARD11_3026321* | hypo | 33(10.25%) | 55(15.85%) |  |  |  |  | 6(8.22.00%) | 14(11.86%) |  |  | |  |  |
|  | hyper | 289(89.75%) | 292(84.15%) | 1.650(1.040-2.616) | 0.033 | 1.707(1.067-2.731) | 0.026 | 67(91.78%) | 104(88.14%) | 1.503(0.551-4.104) | 0.426 | | 1.589(0.563-4.486) | 0.382 |
| *CARD11_3026317* | hypo | 272(84.47%) | 267(76.95%) |  |  |  |  | 61(83.56%) | 91(77.12%) |  |  | |  |  |
|  | hyper | 50(15.53%) | 80(23.05%) | 0.614(0.415-0.908) | 0.009 | 0.603(0.405-0.896) | 0.012 | 12(16.44%) | 27(22.88%) | 0.663(0.312-1.408) | 0.285 | | 0.665(0.307-1.440) | 0.301 |
| *PSMB8_DMR* | hypo | 132(39.76%) | 185(49.47%) |  |  |  |  | 27(36.00%) | 60(48.39%) |  |  | |  |  |
|  | hyper | 200(60.24%) | 189(50.53%) | 1.483(1.100-2.000) | 0.010 | 1.490(1.100-2.018) | 0.010 | 48(64.00%) | 64(51.61%) | 1.667(0.925-3.002) | 0.089 | | 1.803(0.979-3.323) | 0.059 |
| *NCF2_183551942* | hypo | 117(35.35%) | 109(29.07%) |  |  |  |  | 51(57.95%) | 53(39.26%) |  |  | |  |  |
|  | hyper | 214(64.65%) | 266(70.93%) | 0.750(0.546-1.029) | 0.075 | 0.750(0.546-1.029) | 0.054 | 37(42.05%) | 82(60.74%) | 0.469(0.272-0.810) | 0.007 | | 0.495(0.283-0.866) | 0.014 |
| *NCF2_183551969* | hypo | 271(81.87%) | 288(76.80%) |  |  |  |  | 75(85.23%) | 98(72.59%) |  |  | |  |  |
|  | hyper | 60(18.13%) | 87(23.20%) | 0.733(0.507-1.059) | 0.098 | 0.733(0.507-1.059) | 0.101 | 13(14.77%) | 37(27.41%) | 0.459(0.228-0.924) | 0.029 | | 0.386(0.185-0.806) | 0.011 |
| *NCF2_183551986* | hypo | 261(78.85%) | 282(75.20%) |  |  |  |  | 74(84.09%) | 89(65.93%) |  |  | |  |  |
|  | hyper | 70(21.15%) | 93(24.80%) | 0.813(0.571-1.157) | 0.251 | 0.813(0.571-1.157) | 0.206 | 14(15.91%) | 46(34.07%) | 0.366(0.187-0.718) | 0.003 | | 0.379(0.191-0.751) | 0.005 |
| *NCF2_183552095* | hypo | 292(88.22%) | 313(83.47%) |  |  |  |  | 75(85.23%) | 101(74.81%) |  |  | |  |  |
|  | hyper | 39(11.78%) | 62(16.53%) | 0.674(0.438-1.038) | 0.073 | 0.674(0.438-1.038) | 0.073 | 13(14.77%) | 34(25.19%) | 0.515(0.254-1.043) | 0.065 | | 0.530(0.257-1.091) | 0.085 |

CI, confidence interval; OR, odds ratio;^1^ ORs adjusted for age, family history of other cancers and breast cancer. **P* values < 0.025 were considered statistically significant.

**Table S11. The association between methylation level of the CpG sites in five genes and the risk of breast cancer in the subgroup analysis of BC molecular type.**

| **CpG sites^1^** | | **Basal like** | | | | | | **Her2** | | | | | |
| --- | --- | --- | --- | --- | --- | --- | --- | --- | --- | --- | --- | --- | --- |
|  |  |  | | **Univariate analysis** | | **Multivariate analysis** | |  | | **Univariate analysis** | | **Multivariate analysis** | |
|  |  | **Cases (%)** | **Controls (%)** | **OR (95%CI)** | ***P**** | **OR (95%CI)^1^** | ***P**** | **Cases (%)** | **Controls (%)** | **OR (95%CI)** | ***P**** | **OR (95%CI)^1^** | ***P**** |
| *PSMC1_DMR* | hypo | 15(44.12%) | 337(68.08%) |  |  |  |  | 33(67.35%) | 337(68.08%) |  |  |  |  |
|  | hyper | 19(55.88%) | 158(31.92%) | 2.702(1.338-5.456) | 0.006 | 2.721(1.339-5.527) | 0.006 | 16(32.65%) | 158(31.92%) | 1.034(0.553-1.934) | 0.916 | 0.984(0.521-1.861) | 0.961 |
| *SPPL3_121202552* | hypo | 15(44.12%) | 158(36.41%) |  |  |  |  | 19(46.34%) | 158(36.41%) |  |  |  |  |
|  | hyper | 19(55.88%) | 276(63.59%) | 0.725(0.358-1.467) | 0.371 | 0.702(0.345-1.429) | 0.330 | 22(53.66%) | 276(63.59%) | 0.663(0.348-1.262) | 0.211 | 0.612(0.318-1.179) | 0.142 |
| *SPPL3_121202554* | hypo | 29(85.29%) | 295(67.97%) |  |  |  |  | 35(85.37%) | 295(67.97%) |  |  |  |  |
|  | hyper | 5(14.71%) | 139(32.03%) | 0.366(0.139-0.966) | 0.042 | 0.376(0.142-0.999) | 0.050 | 6(14.63%) | 139(32.03%) | 0.364(0.150-0.885) | 0.026 | 0.348(0.141-0.859) | 0.022 |
| *SPPL3_121202602* | hypo | 20(58.82%) | 297(68.43%) |  |  |  |  | 24(58.54%) | 297(68.43%) |  |  |  |  |
|  | hyper | 14(41.18%) | 137(31.57%) | 1.518(0.744-3.094) | 0.251 | 1.503(0.730-3.092) | 0.269 | 17(41.46%) | 137(31.57%) | 1.536(0.799-2.952) | 0.198 | 1.451(0.747-2.817) | 0.272 |
| *CARD11_3026460* | hypo | 29(82.86%) | 393(82.91%) |  |  |  |  | 43(93.48%) | 393(82.91%) |  |  |  |  |
|  | hyper | 6(17.14%) | 81(17.09%) | 1.004(0.404-2.496) | 0.993 | 1.010(0.403-2.531) | 0.984 | 3(6.52%) | 81(17.09%) | 0.339(0.103-1.118) | 0.076 | 0.349(0.105-1.157) | 0.085 |
| *CARD11_3026436* | hypo | 9(25.71%) | 142(29.96%) |  |  |  |  | 9(19.57%) | 142(29.96%) |  |  |  |  |
|  | hyper | 26(74.29%) | 332(70.04%) | 1.236(0.565-2.704) | 0.596 | 1.195(0.541-2.639) | 0.659 | 37(80.43%) | 332(70.04%) | 1.758(0.827-3.739) | 0.143 | 1.672(0.783-3.570) | 0.184 |
| *CARD11_3026433* | hypo | 12(34.29%) | 161(33.97%) |  |  |  |  | 17(36.96%) | 161(33.97%) |  |  |  |  |
|  | hyper | 23(65.71%) | 313(66.03%) | 0.986(0.478-2.032) | 0.969 | 0.978(0.472-2.027) | 0.952 | 29(63.04%) | 313(66.03%) | 0.877(0.468-1.644) | 0.683 | 0.881(0.468-1.660) | 0.696 |
| *CARD11_3026389* | hypo | 2(5.71%) | 70(14.77%) |  |  |  |  | 5(10.87%) | 70(14.77%) |  |  |  |  |
|  | hyper | 33(94.29%) | 404(85.23%) | 2.859(0.671-12.184) | 0.156 | 2.994(0.697-12.873) | 0.140 | 41(89.13%) | 404(85.23%) | 1.421(0.543-3.720) | 1.421 | 1.345(0.510-3.544) | 0.549 |
| *CARD11_3026375* | hypo | 6(17.14%) | 124(26.16%) |  |  |  |  | 8(17.39%) | 124(26.16%) |  |  |  |  |
|  | hyper | 29(82.86%) | 350(73.84%) | 1.712(0.694-4.223) | 0.243 | 1.804(0.725-4.487) | 0.205 | 38(82.61%) | 350(73.84%) | 1.683(0.764-3.706) | 0.196 | 1.751(0.791-3.877) | 0.167 |
| *CARD11_3026321* | hypo | 4(11.43%) | 70(14.77%) |  |  |  |  | 5(10.87%) | 70(14.77%) |  |  |  |  |
|  | hyper | 31(88.57%) | 404(85.23%) | 1.343(0.460-3.922) | 0.590 | 1.393(0.472-4.107) | 0.548 | 41(89.13%) | 404(85.23%) | 1.421(0.543-3.720) | 0.474 | 1.404(0.534-3.692) | 0.492 |
| *CARD11_3026317* | hypo | 30(85.71%) | 364(76.79%) |  |  |  |  | 40(86.96%) | 364(76.79%) |  |  |  |  |
|  | hyper | 5(14.29%) | 110(23.21%) | 0.552(0.209-1.456) | 0.229 | 0.556(0.210-1.473) | 0.238 | 6(13.04%) | 110(23.21%) | 0.496(0.205-1.202) | 0.120 | 0.471(0.191-1.156) | 0.100 |
| *PSMB8_DMR* | hypo | 13(37.14%) | 251(49.51%) |  |  |  |  | 21(43.75%) | 251(49.51%) |  |  |  |  |
|  | hyper | 22(62.86%) | 256(50.49%) | 1.659(0.818-3.366) | 0.161 | 1.674(0.820-3.416) | 0.157 | 27(56.25%) | 256(50.49%) | 1.261(0.694-2.288) | 0.446 | 1.245(0.681-2.276) | 0.476 |
| *NCF2_183551942* | hypo | 7(20.00%) | 163(32.09%) |  |  |  |  | 27(55.10%) | 163(32.09%) |  |  |  |  |
|  | hyper | 28(80.00%) | 345(67.91%) | 1.890(0.809-4.417) | 0.142 | 0.750(0.546-1.029) | 0.146 | 22(44.90%) | 345(67.91%) | 0.385(0.213-0.697) | 0.002 | 0.382(0.209-0.696) | 0.002 |
| *NCF2_183551969* | hypo | 28(80.00%) | 387(76.18%) |  |  |  |  | 38(77.55%) | 387(76.18%) |  |  |  |  |
|  | hyper | 7(20.00%) | 121(23.82%) | 0.800(0.341-1.876) | 0.607 | 0.733(0.507-1.059) | 0.630 | 11(22.45%) | 121(23.82%) | 0.926(0.459-1.867) | 0.830 | 0.882(0.432-1.802) | 0.731 |
| *NCF2_183551986* | hypo | 22(62.86%) | 371(73.03%) |  |  |  |  | 41(83.67%) | 371(73.03%) |  |  |  |  |
|  | hyper | 13(37.14%) | 137(26.97%) | 1.600(0.784-3.265) | 0.196 | 0.813(0.571-1.157) | 0.181 | 8(16.33%) | 137(26.97%) | 0.528(0.242-1.156) | 0.110 | 0.502(0.226-1.113) | 0.090 |
| *NCF2_183552095* | hypo | 32(91.43%) | 414(81.50%) |  |  |  |  | 38(77.55%) | 414(81.50%) |  |  |  |  |
|  | hyper | 3(8.57%) | 94(18.5%) | 0.413(0.124-1.377) | 0.150 | 0.674(0.438-1.038) | 0.160 | 11(22.45%) | 94(18.50%) | 1.275(0.628-2.587) | 0.501 | 1.266(0.620-2.586) | 0.518 |

CI, confidence interval; OR, odds ratio;^1^ ORs adjusted for age, family history of other cancer and breast cancer. **P* values < 0.0125 were considered statistically significant.

**Table S12. The association between methylation level of the CpG sites in 5 genes and the risk of breast cancer in the subgroup analysis of BC molecular type.**

|  |  | **Luminal_A** | | | | | | | **Luminal_B** | | | | | |
| --- | --- | --- | --- | --- | --- | --- | --- | --- | --- | --- | --- | --- | --- | --- |
| **CpG sites^1^** | |  | | **Univariate analysis** | | | **Multivariate analysis** | |  | | **Univariate analysis** | | **Multivariate analysis** | |
|  |  | **Cases (%)** | **Controls (%)** | | **OR (95%CI)** | ***P**** | **OR (95%CI)^1^** | ***P**** | **Cases (%)** | **Controls (%)** | **OR (95%CI)** | ***P**** | **OR (95%CI)^1^** | ***P**** |
| *PSMC1_DMR* | hypo | 83(58.04%) | 337(68.08%) | |  |  |  |  | 80(55.60%) | 337(68.08%) |  |  |  |  |
|  | hyper | 60(41.96%) | 158(31.92%) | | 1.542(1.052-2.259) | 0.026 | 1.517(1.032-2.228) | 0.034 | 64(44.40%) | 158(31.92%) | 2.133(1.484-3.066) | <0.001 | 2.128(1.466-3.090) | <0.001 |
| *SPPL3_121202552* | hypo | 60(45.8%) | 158(36.41%) | |  |  |  |  | 69(47.92%) | 158(36.41%) |  |  |  |  |
|  | hyper | 71(54.2%) | 276(63.59%) | | 0.677(0.456-1.006) | 0.054 | 0.660(0.442-0.986) | 0.042 | 75(52.08%) | 276(63.59%) | 0.622(0.425-0.911) | 0.015 | 0.619(0.418-0.916) | 0.016 |
| *SPPL3_121202554* | hypo | 102(77.86%) | 295(67.97%) | |  |  |  |  | 104(72.22%) | 295(67.97%) |  |  |  |  |
|  | hyper | 29(22.14%) | 139(32.03%) | | 0.603(0.381-0.955) | 0.031 | 0.606(0.380-0.968) | 0.036 | 40(27.78%) | 139(32.03%) | 0.816(0.538-1.238) | 0.340 | 0.813(0.530-1.248) | 0.344 |
| *SPPL3_121202602* | hypo | 86(65.65%) | 297(68.43%) | |  |  |  |  | 83(57.64%) | 297(68.43%) |  |  |  |  |
|  | hyper | 45(34.35%) | 137(31.57%) | | 1.134(0.750-1.715) | 0.550 | 1.102(0.725-1.676) | 0.649 | 61(42.36%) | 137(31.57%) | 1.593(1.081-2.348) | 0.019 | 1.58(1.061-2.352) | 0.024 |
| *CARD11_3026460* | hypo | 126(88.73%) | 393(82.91%) | |  |  |  |  | 140(91.50%) | 393(82.91%) |  |  |  |  |
|  | hyper | 16(11.27%) | 81(17.09%) | | 0.616(0.348-1.092) | 0.097 | 0.625(0.352-1.112) | 0.110 | 13(8.50%) | 81(17.09%) | 0.451(0.243-0.835) | 0.011 | 0.409(0.214-0.781) | 0.007 |
| *CARD11_3026436* | hypo | 26(18.31%) | 142(29.96%) | |  |  |  |  | 39(25.49%) | 142(29.96%) |  |  |  |  |
|  | hyper | 116(81.69%) | 332(70.04%) | | 1.908(1.194-3.049) | 0.007 | 1.885(1.168-3.044) | 0.009 | 114(74.51%) | 332(70.04%) | 1.250(0.827-1.890) | 0.290 | 1.159(0.760-1.769) | 0.494 |
| *CARD11_3026433* | hypo | 39(27.46%) | 161(33.97%) | |  |  |  |  | 34(22.22%) | 161(33.97%) |  |  |  |  |
|  | hyper | 103(72.54%) | 313(66.03%) | | 1.358(0.897-2.057) | 0.148 | 1.331(0.876-2.021) | 0.180 | 119(77.78%) | 313(66.03%) | 1.800(1.176-2.756) | 0.007 | 1.858(1.200-2.876) | 0.005 |
| *CARD11_3026389* | hypo | 15(10.56%) | 70(14.77%) | |  |  |  |  | 10(6.54%) | 70(14.77%) |  |  |  |  |
|  | hyper | 127(89.44%) | 404(85.23%) | | 1.467(0.811-2.652) | 0.205 | 1.462(0.804-2.656) | 0.213 | 143(93.46%) | 404(85.23%) | 2.478(1.243-4.937) | 0.010 | 2.549(1.258-5.166) | 0.009 |
| *CARD11_3026375* | hypo | 30(21.13%) | 124(26.16%) | |  |  |  |  | 32(20.92%) | 124(26.16%) |  |  |  |  |
|  | hyper | 112(78.87%) | 350(73.84%) | | 1.323(0.842-2.079) | 0.225 | 1.378(0.871-2.182) | 0.171 | 121(79.08%) | 350(73.84%) | 1.340(0.863-2.080) | 0.193 | 1.462(0.928-2.305) | 0.102 |
| *CARD11_3026321* | hypo | 14(9.86%) | 70(14.77%) | |  |  |  |  | 11(7.19%) | 70(14.77%) |  |  |  |  |
|  | hyper | 128(90.14%) | 404(85.23%) | | 1.584(0.863-2.908) | 0.138 | 1.596(0.866-2.944) | 0.134 | 142(92.81%) | 404(85.23%) | 2.237(1.152-4.344) | 0.017 | 2.352(1.188-4.658) | 0.014 |
| *CARD11_3026317* | hypo | 123(86.62%) | 364(76.79%) | |  |  |  |  | 124(81.05%) | 364(76.79%) |  |  |  |  |
|  | hyper | 19(13.38%) | 110(23.21%) | | 0.511(0.301-0.867) | 0.013 | 0.531(0.312-0.903) | 0.019 | 29(18.95%) | 110(23.21%) | 0.774(0.490-1.222) | 0.272 | 0.768(0.480-1.228) | 0.270 |
| *PSMB8_DMR* | hypo | 64(44.14%) | 251(49.51%) | |  |  |  |  | 51(31.88%) | 251(49.51%) |  |  |  |  |
|  | hyper | 81(55.86%) | 256(50.49%) | | 1.241(0.856-1.799) | 0.254 | 1.258(0.866-1.827) | 0.229 | 109(68.13%) | 256(50.49%) | 2.096(1.440-3.050) | 0.000 | 2.208(1.496-3.257) | <0.001 |
| *NCF2_183551942* | hypo | 63(43.75%) | 163(32.09%) | |  |  |  |  | 58(36.25%) | 163(32.09%) |  |  |  |  |
|  | hyper | 81(56.25%) | 345(67.91%) | | 0.607(0.416-0.887) | 0.010 | 0.602(0.412-0.881) | 0.009 | 102(63.75%) | 345(67.91%) | 0.831(0.573-1.206) | 0.329 | 0.867(0.591-1.274) | 0.468 |
| *NCF2_183551969* | hypo | 122(84.72%) | 387(76.18%) | |  |  |  |  | 135(84.38%) | 387(76.18%) |  |  |  |  |
|  | hyper | 22(15.28%) | 121(23.82%) | | 0.577(0.351-0.949) | 0.030 | 0.570(0.345-0.941) | 0.028 | 25(15.63%) | 121(23.82%) | 0.592(0.369-0.951) | 0.030 | 0.610(0.376-0.990) | 0.045 |
| *NCF2_**183551986* | hypo | 125(86.81%) | 371(73.03%) | |  |  |  |  | 127(79.38%) | 371(73.03%) |  |  |  |  |
|  | hyper | 19(13.19%) | 137(26.97%) | | 0.412(0.245-0.693) | 0.001 | 0.396(0.234-0.672) | 0.001 | 33(20.63%) | 137(26.97%) | 0.704(0.458-1.082) | 0.109 | 0.679(0.435-1.059) | 0.088 |
| *NCF2_183552095* | hypo | 130(90.28%) | 414(81.5%) | |  |  |  |  | 141(88.13%) | 414(81.50%) |  |  |  |  |
|  | hyper | 14(9.72%) | 94(18.5%) | | 0.474(0.262-0.860) | 0.014 | 0.484(0.267-0.879) | 0.017 | 19(11.88%) | 94(18.50%) | 0.593(0.35-1.007) | 0.053 | 0.567(0.327-0.981) | 0.043 |

CI, confidence interval; OR, odds ratio;^1^ ORs adjusted for age, family history of other cancers and breast cancer. **P* values < 0.0125 were considered statistically significant.

**Table S13. The association between methylation level of the CpG sites in 5 genes and the risk of breast cancer in the subgroup analysis of ER expression.**

|  |  | **ER_negative** | | | | | | | **ER_positive** | | | | | |
| --- | --- | --- | --- | --- | --- | --- | --- | --- | --- | --- | --- | --- | --- | --- |
| **CpG sites^1^** | |  | | **Univariate analysis** | | | **Multivariate analysis** | |  | | **Univariate analysis** | | **Multivariate analysis** | |
|  |  | **Cases (%)** | **Controls (%)** | | **OR (95%CI)** | ***P**** | **OR (95%CI)^1^** | ***P**** | **Cases (%)** | **Controls (%)** | **OR (95%CI)** | ***P**** | **OR (95%CI)^1^** | ***P**** |
| *PSMC1_DMR* | hypo | 51(56.04%) | 337(68.08%) | |  |  |  |  | 173(54.75%) | 337(68.08%) |  |  |  |  |
|  | hyper | 40(43.96%) | 158(31.92%) | | 1.673(1.061-2.637) | 0.027 | 1.647(1.039-2.611) | 0.034 | 143(45.25%) | 158(31.92%) | 1.763(1.318-2.359) | 0.000 | 1.717(1.278-2.308) | <0.001 |
| *SPPL3_121202552* | hypo | 37(44.05%) | 158(36.41%) | |  |  |  |  | 135(47.04%) | 158(36.41%) |  |  |  |  |
|  | hyper | 47(55.95%) | 276(63.59%) | | 0.727(0.453-1.167) | 0.187 | 0.693(0.429-1.119) | 0.134 | 152(52.96%) | 276(63.59%) | 0.645(0.476-0.873) | 0.005 | 0.639(0.468-0.871) | 0.005 |
| *SPPL3_121202554* | hypo | 68(80.95%) | 295(67.97%) | |  |  |  |  | 214(74.56%) | 295(67.97%) |  |  |  |  |
|  | hyper | 16(19.05%) | 139(32.03%) | | 0.499(0.279-0.893) | 0.019 | 0.504(0.280-0.907) | 0.022 | 73(25.44%) | 139(32.03%) | 0.724(0.519-1.011) | 0.058 | 0.715(0.508-1.006) | 0.054 |
| *SPPL3_121202602* | hypo | 47(55.95%) | 297(68.43%) | |  |  |  |  | 177(61.67%) | 297(68.43%) |  |  |  |  |
|  | hyper | 37(44.05%) | 137(31.57%) | | 1.707(1.06-2.747) | 0.028 | 1.639(1.012-2.654) | 0.066 | 110(38.33%) | 137(31.57%) | 1.347(0.986-1.841) | 0.061 | 1.323(0.963-1.819) | 0.085 |
| *CARD11_3026460* | hypo | 80(88.89%) | 393(82.91%) | |  |  |  |  | 280(90.61%) | 393(82.91%) |  |  |  |  |
|  | hyper | 10(11.11%) | 81(17.09%) | | 0.606(0.301-1.221) | 0.161 | 0.606(0.299-1.228) | 0.164 | 29(9.39%) | 81(17.09%) | 0.503(0.32-0.789) | 0.003 | 0.480(0.302-0.763) | 0.002 |
| *CARD11_3026436* | hypo | 18(20.00%) | 142(29.96%) | |  |  |  |  | 68(22.01%) | 142(29.96%) |  |  |  |  |
|  | hyper | 72(80.00%) | 332(70.04%) | | 1.711(0.984-2.973) | 0.057 | 1.639(0.939-2.861) | 0.082 | 241(77.99%) | 332(70.04%) | 1.516(1.086-2.115) | 0.014 | 1.455(1.035-2.045) | 0.031 |
| *CARD11_3026433* | hypo | 32(35.56%) | 161(33.97%) | |  |  |  |  | 78(25.24%) | 161(33.97%) |  |  |  |  |
|  | hyper | 58(64.44%) | 313(66.03%) | | 0.932(0.582-1.494) | 0.771 | 0.938(0.583-1.510) | 0.793 | 231(74.76%) | 313(66.03%) | 1.523(1.107-2.097) | 0.010 | 1.544(1.114-2.139) | 0.009 |
| *CARD11_3026389* | hypo | 9(10.00%) | 70(14.77%) | |  |  |  |  | 25(8.09%) | 70(14.77%) |  |  |  |  |
|  | hyper | 81(90.00%) | 404(85.23%) | | 1.559(0.749-3.249) | 0.235 | 1.521(0.726-3.189) | 0.267 | 284(91.91%) | 404(85.23%) | 1.968(1.216-3.185) | 0.006 | 2.023(1.237-3.309) | 0.005 |
| *CARD11_3026375* | hypo | 15(16.67%) | 124(26.16%) | |  |  |  |  | 66(21.36%) | 124(26.16%) |  |  |  |  |
|  | hyper | 75(83.33%) | 350(73.84%) | | 1.771(0.981-3.199) | 0.058 | 1.874(1.031-3.406) | 0.040 | 243(78.64%) | 350(73.84%) | 1.304(0.928-1.834) | 0.126 | 1.404(0.990-1.991) | 0.057 |
| *CARD11_3026321* | hypo | 11(12.22%) | 70(14.77%) | |  |  |  |  | 28(9.06%) | 70(14.77%) |  |  |  |  |
|  | hyper | 79(87.78%) | 404(85.23%) | | 1.244(0.630-2.456) | 0.529 | 1.274(0.641-2.533) | 0.490 | 281(90.94%) | 404(85.23%) | 1.739(1.093-2.765) | 0.019 | 1.784(1.111-2.866) | 0.017 |
| *CARD11_3026317* | hypo | 78(86.67%) | 364(76.79%) | |  |  |  |  | 259(83.82%) | 364(76.79%) |  |  |  |  |
|  | hyper | 12(13.33%) | 110(23.21%) | | 0.509(0.267-0.969) | 0.040 | 0.502(0.262-0.964) | 0.038 | 50(16.18%) | 110(23.21%) | 0.639(0.441-0.925) | 0.018 | 0.653(0.448-0.952) | 0.027 |
| *PSMB8_DMR* | hypo | 37(40.22%) | 251(49.51%) | |  |  |  |  | 124(38.87%) | 251(49.51%) |  |  |  |  |
|  | hyper | 55(59.78%) | 256(50.49%) | | 1.457(0.928-2.289) | 0.102 | 1.469(0.929-2.321) | 0.100 | 195(61.13%) | 256(50.49%) | 1.542(1.16-2.049) | 0.003 | 1.587(1.187-2.121) | 0.002 |
| *NCF2_183551942* | hypo | 38(40.86%) | 163(32.09%) | |  |  |  |  | 125(39.31%) | 163(32.09%) |  |  |  |  |
|  | hyper | 55(59.14%) | 345(67.91%) | | 0.684(0.435-1.076) | 0.100 | 0.750(0.546-1.029) | 0.099 | 193(60.69%) | 345(67.91%) | 0.729(0.545-0.977) | 0.034 | 0.734(0.546-0.988) | 0.041 |
| *NCF2_183551969* | hypo | 70(75.27%) | 387(76.18%) | |  |  |  |  | 269(84.59%) | 387(76.18%) |  |  |  |  |
|  | hyper | 23(24.73%) | 121(23.82%) | | 1.051(0.629-1.756) | 0.850 | 0.733(0.507-1.059) | 0.885 | 49(15.41%) | 121(23.82%) | 0.583(0.404-0.841) | 0.004 | 0.584(0.402-0.849) | 0.005 |
| *NCF2_183551986* | hypo | 67(72.04%) | 371(73.03%) | |  |  |  |  | 262(82.39%) | 371(73.03%) |  |  |  |  |
|  | hyper | 26(27.96%) | 137(26.97%) | | 1.051(0.642-1.721) | 0.742 | 0.813(0.571-1.157) | 0.817 | 56(17.61%) | 137(26.97%) | 0.579(0.408-0.82) | 0.002 | 0.555(0.388-0.794) | 0.001 |
| *NCF2_183552095* | hypo | 76(81.72%) | 414(81.50%) | |  |  |  |  | 284(89.31%) | 414(81.50%) |  |  |  |  |
|  | hyper | 17(18.28%) | 94(18.50%) | | 0.985(0.556-1.745) | 0.959 | 0.674(0.438-1.038) | 0.965 | 34(10.69%) | 94(18.50%) | 0.527(0.346-0.803) | 0.003 | 0.523(0.340-0.803) | 0.003 |

CI, confidence interval; OR, odds ratio;^1^ ORs adjusted for age, family history of other cancers and breast cancer. **P* values < 0.025 were considered statistically significant.

**Table S14. The association between five CpGs of immune-related genes and the risk of breast cancer after adjusting the distribution of six cells.**

| **CpG site** |  | **OR (95% CI)** | ***P* value** | **OR (95% CI)^1^** | ***P* value** |
| --- | --- | --- | --- | --- | --- |
| cg01760846 |  |  |  |  |  |
|  | hypo | 2.250(1.583-3.198) | <0.001 | 2.241(1.567-3.205) | <0.001 |
|  | hyper |  |  |  |  |
| cg07141527 |  |  |  |  |  |
|  | hypo | 0.433(0.301-0.625) | <0.001 | 0.435(0.301-0.629) | <0.001 |
|  | hyper |  |  |  |  |
| cg15658543 |  |  |  |  |  |
|  | hypo | 0.470(0.333-0.663) | <0.001 | 0.476(0.337-0.672) | <0.001 |
|  | hyper |  |  |  |  |
| cg21568368 |  |  |  |  |  |
|  | hypo | 0.587(0.414-0.832) | 0.003 | 0.588(0.414-0.834) | 0.003 |
|  | hyper |  |  |  |  |
| cg24045276 |  |  |  |  |  |
|  | hypo | 0.519(0.362-0.743) | <0.001 | 0.503(0.350-0.724) | <0.001 |
|  | hyper |  |  |  |  |

CI, confidence interval; OR, odds ratio;^1^ ORs adjusted for age and proportion of cell types.

**P* values < 0.05 were considered statistically significant.


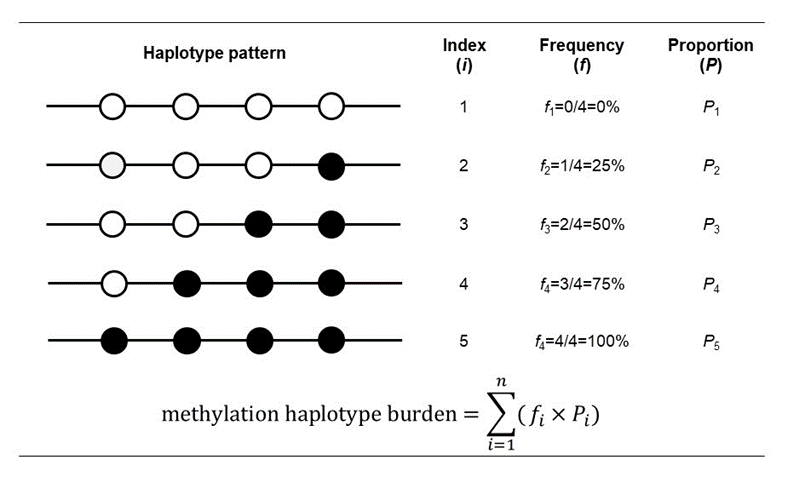


**Figure S1. ﻿Schematic representation of methylation haplotype burden.**

The diagram is an example for a genome region with 4 CpG sites and 5 patterns of methylation haplotypes in a sequencing sample. The black circles represent the methylated CpGs, and white circles represent the unmethylated CpGs. fi is the frequency of DNA methylation at the CpG sites in the targeted region; Pi is the number of reads of the haplotype divided by the total number of reads in the targeted region. Based on the formula shown below the figure, which is weighted by the frequency of methylation in the targeted sequencing region, we can calculate the methylation haplotype burden.

**
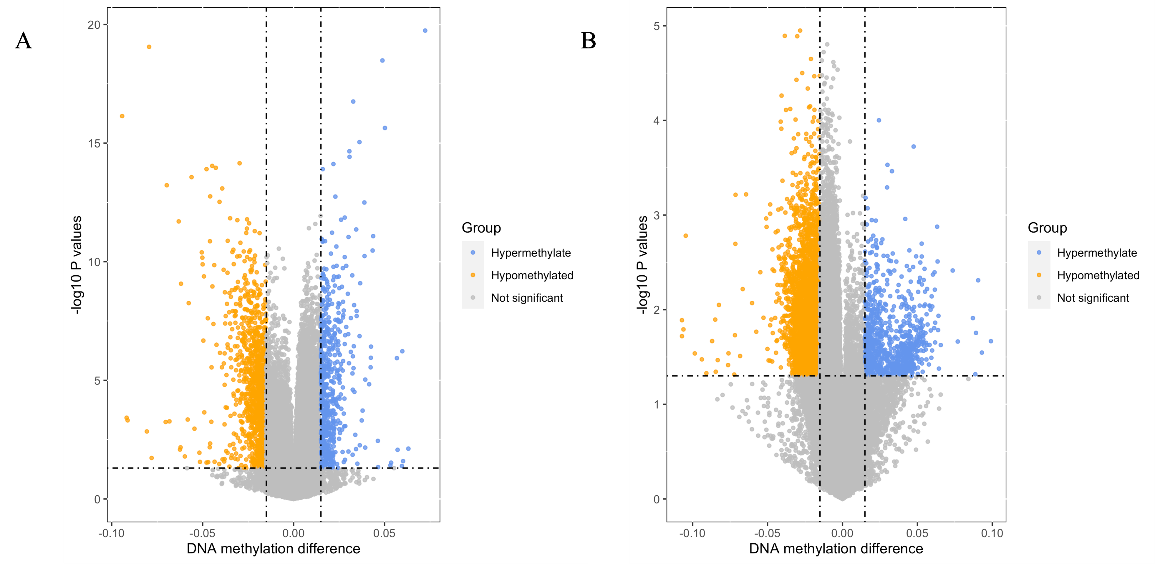
**

**Figure S2. Volcano plots of differential methylation analysis in the samples from GSE51032 and** **GSE104942.**

A represents the volcano plot of the sample from GSE51032; B represents the volcano plot of the samples from GSE104942.


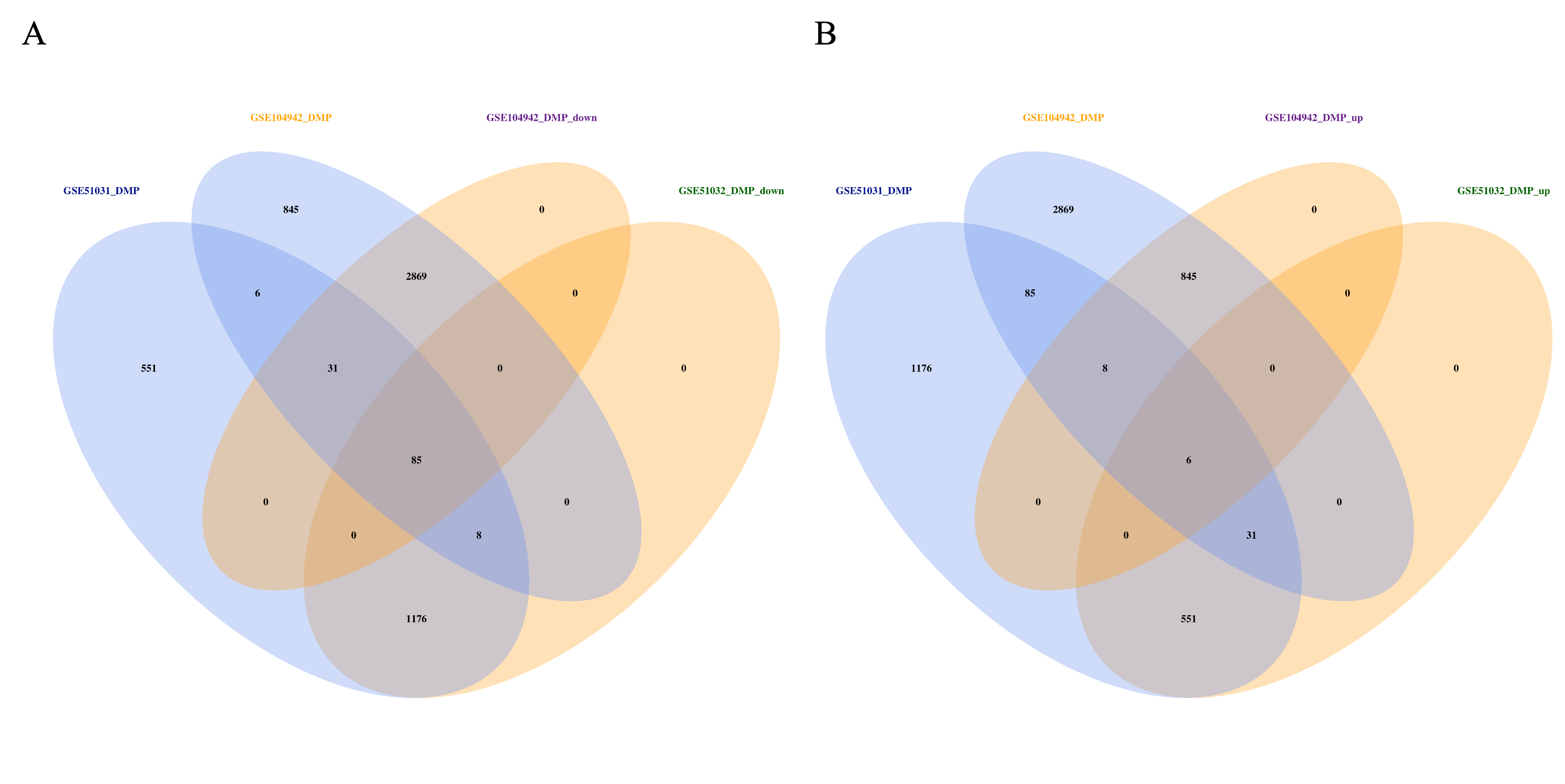


**Figure S3. ﻿Integrative analysis of DNA methylation.**

**﻿**A four-way venn diagram shows intersection of genes containing differentially methylated. A represents the venn diagram of hypomethylation of CpG sites associated to immune; B represents the venn diagram of hypermethylation of CpG sites associated to immune.

**
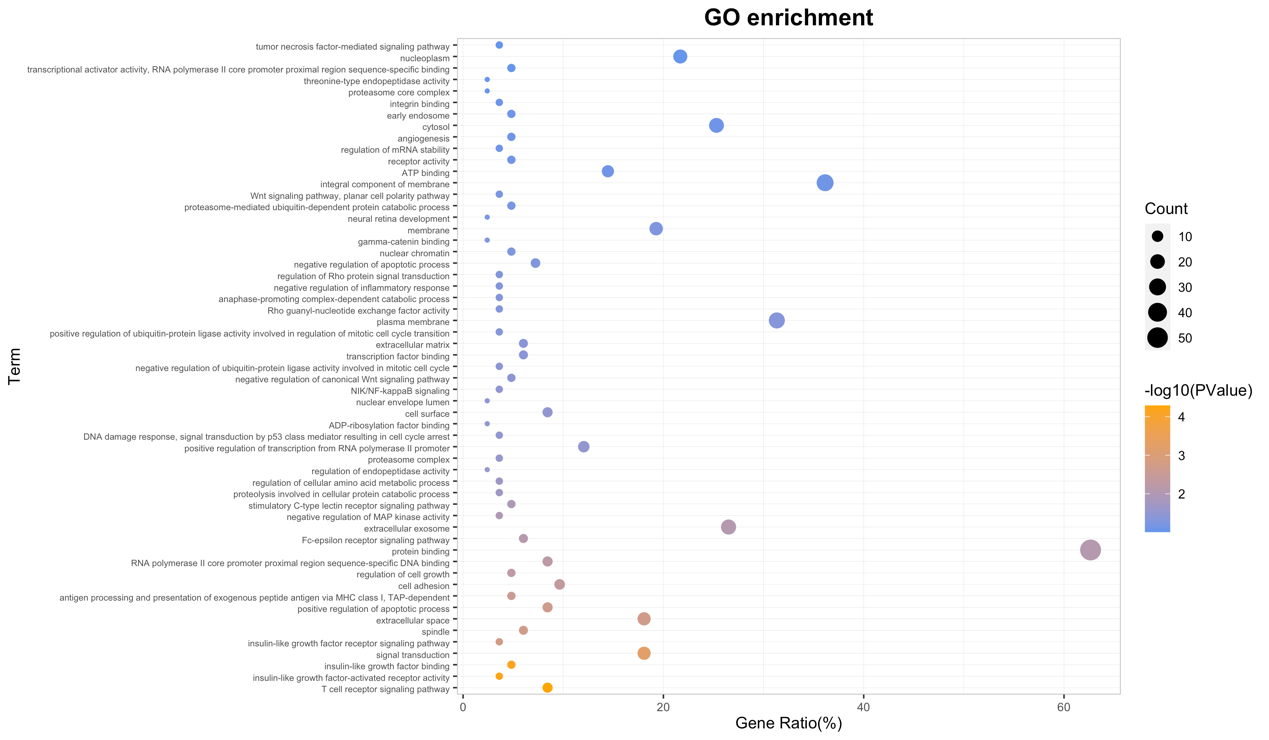
**

**Figure S4. A dotplot of Go enrichment analysis.**

The X-axis represents the gene ratio, which means the ratio of genes to the total genes in a term. The Y-axis rest represents the functional terms. The color represents the *P* value. The size of the dot represents the number of the enriched genes


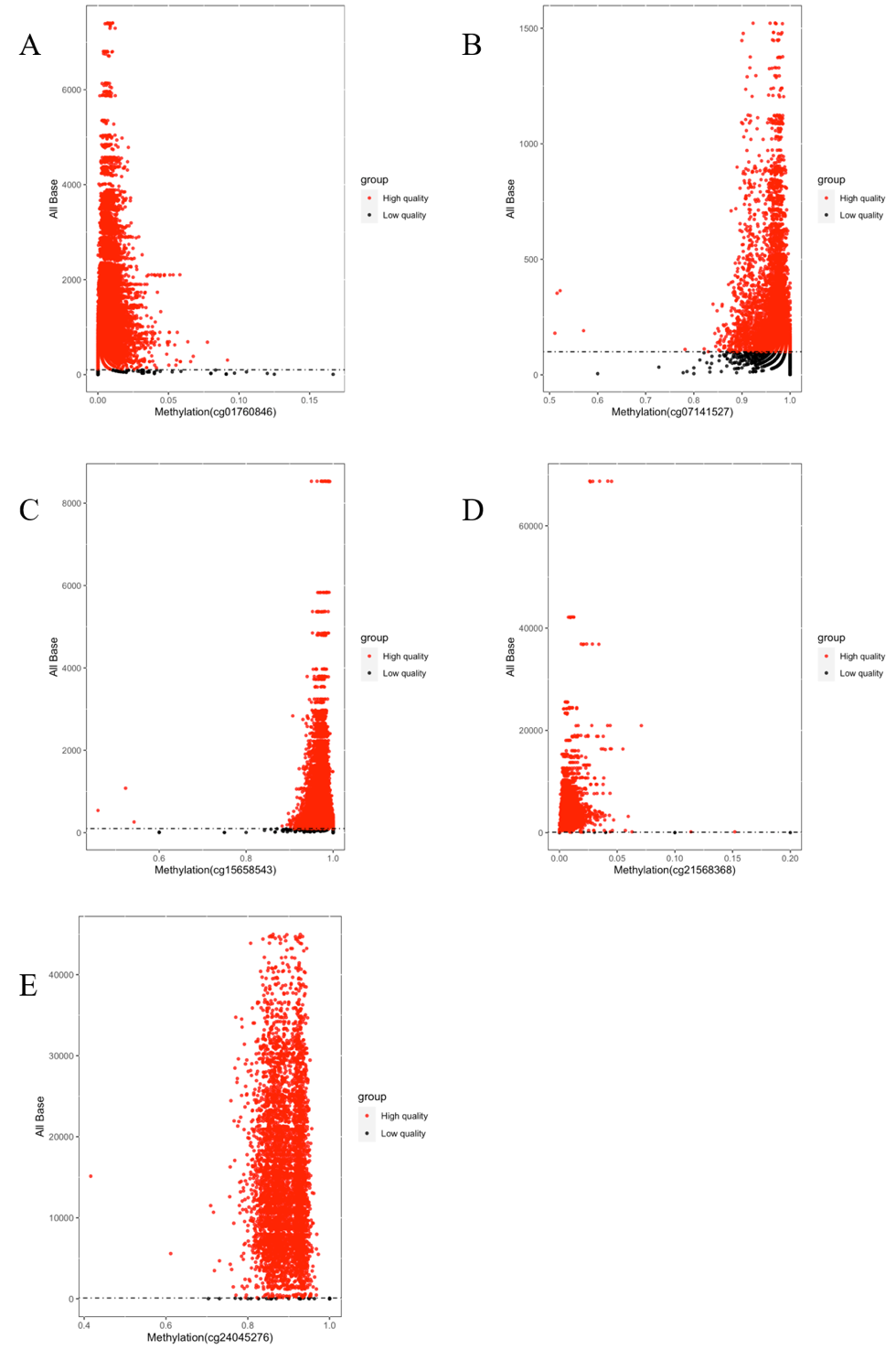


**Figure S5. Quality control of samples based on different target regions.**

(A), (B), (C), (D) ,(E),represent the sequencing depth of cg01760846, cg07141527, cg15658543, cg21568368 cg24045276 in targeted sequencing, respectively. The red points represent the samples that the sequencing depth are greater than 100X.


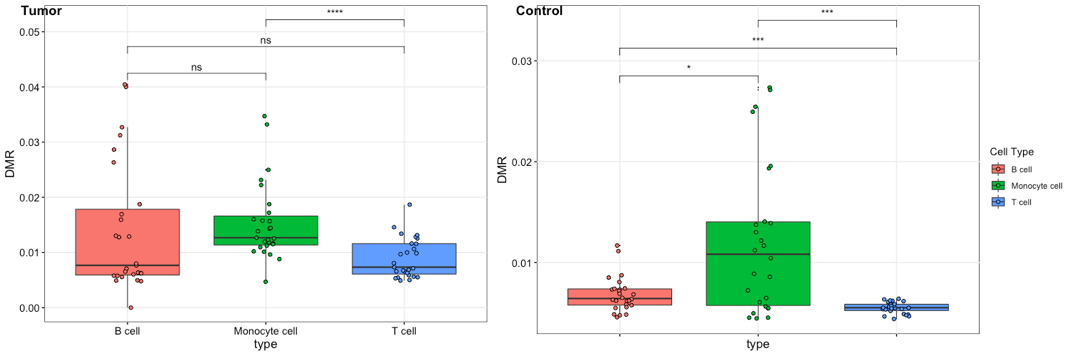


**Figure S6.** **Distribution of *PSMC1*_DMR methylation among sorted leukocyte fractions**


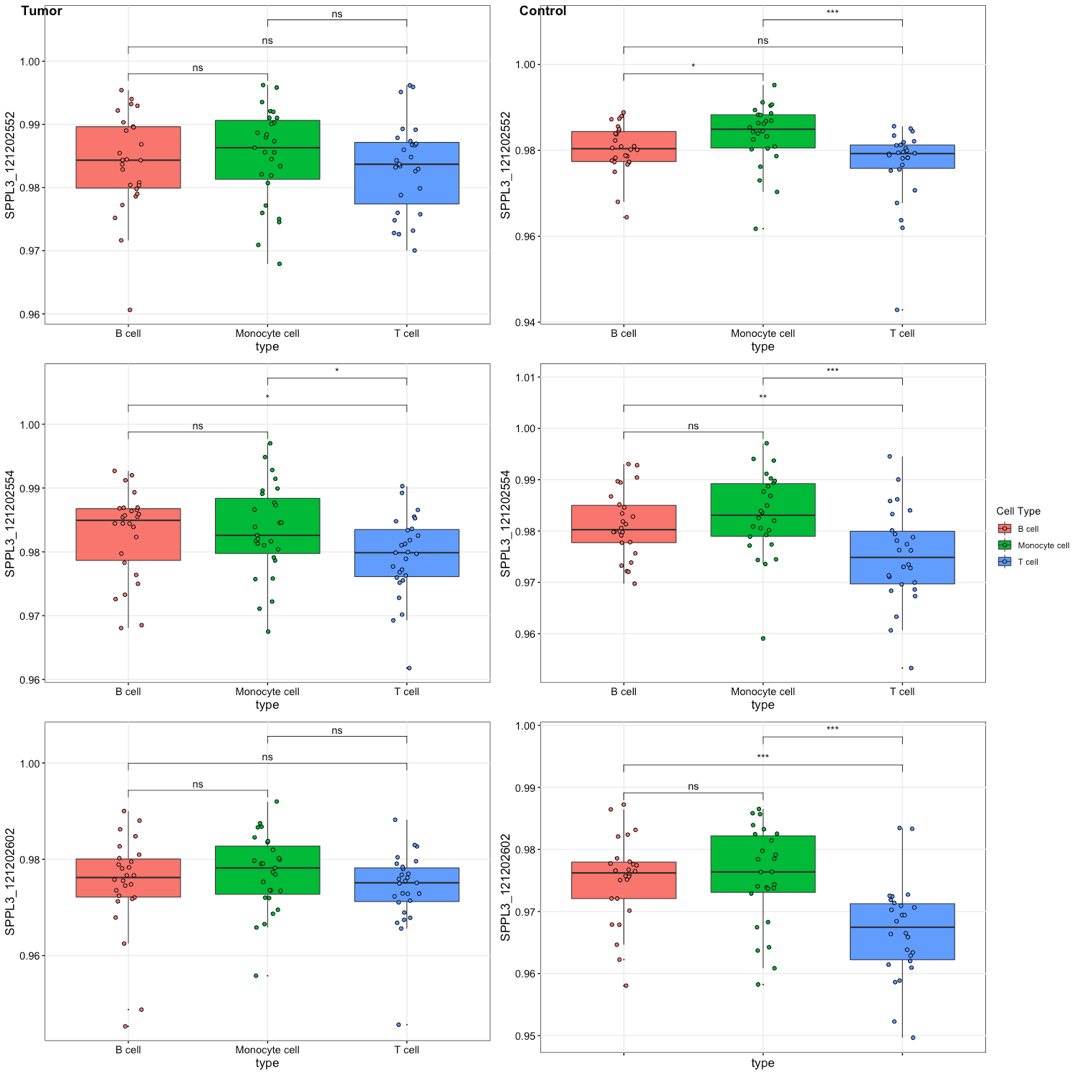


**Figure S7. Distribution of the methylation of 3 CpG sites in *SPPL3* region among sorted leukocyte fractions.**


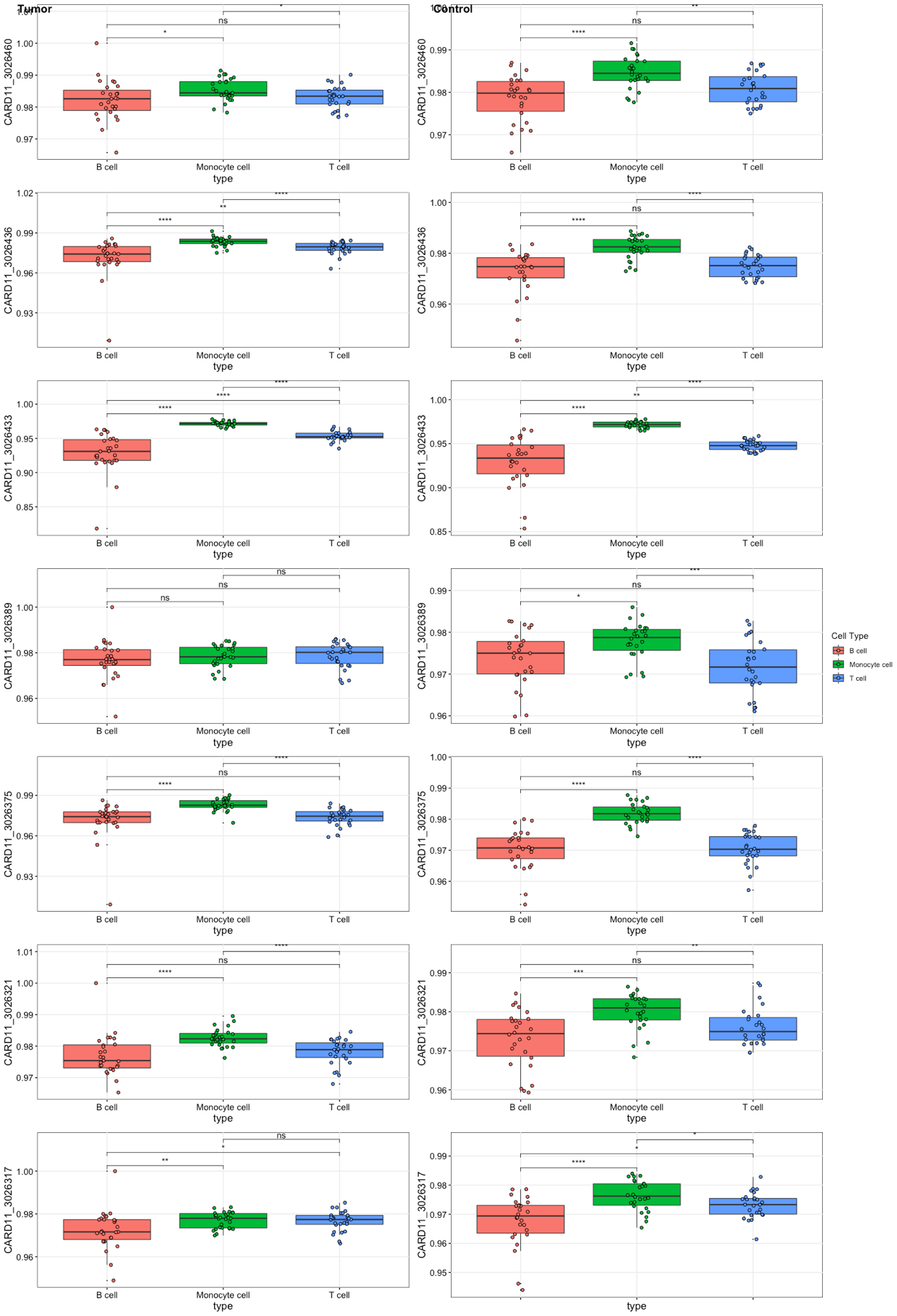


**Figure S8. Distribution of the methylation of 7 CpG sites in *CARD11* region among sorted leukocyte fractions.**

**
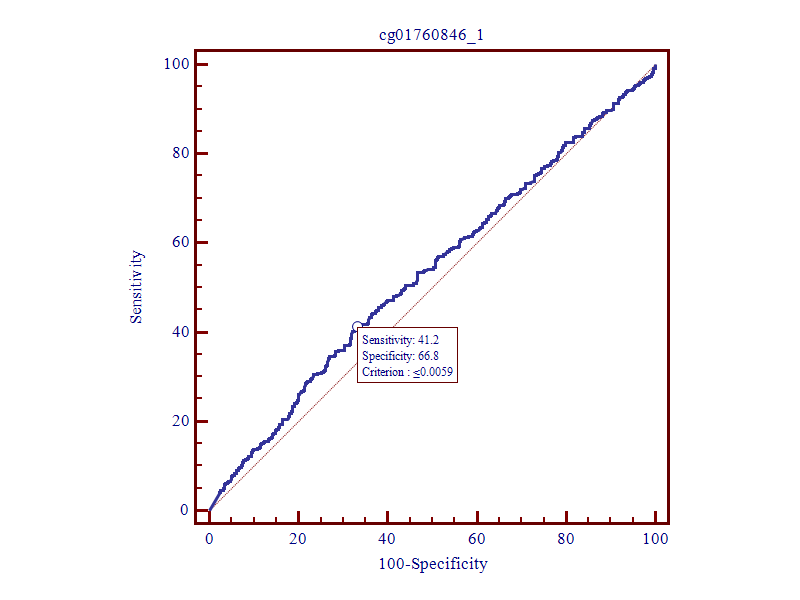
**

**Figure S9. ROC curves of PSMC1_90722706.**

**
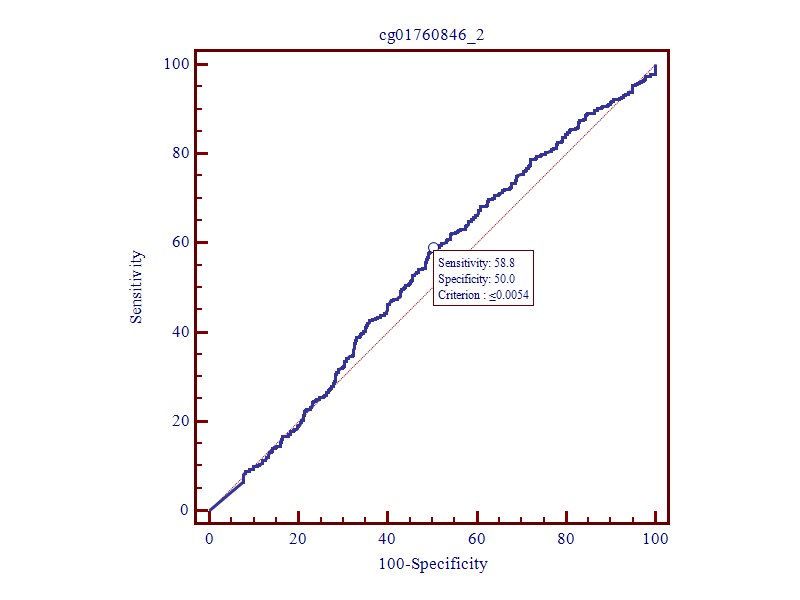
**

**Figure S10. ROC curves of PSMC1_90722716.**

**
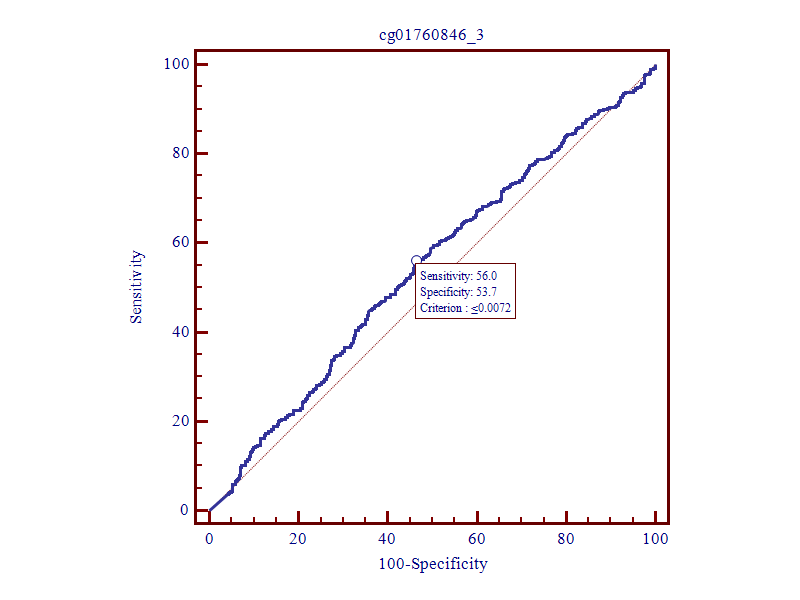
**

**Figure S11. ROC curves of PSMC1_90722782
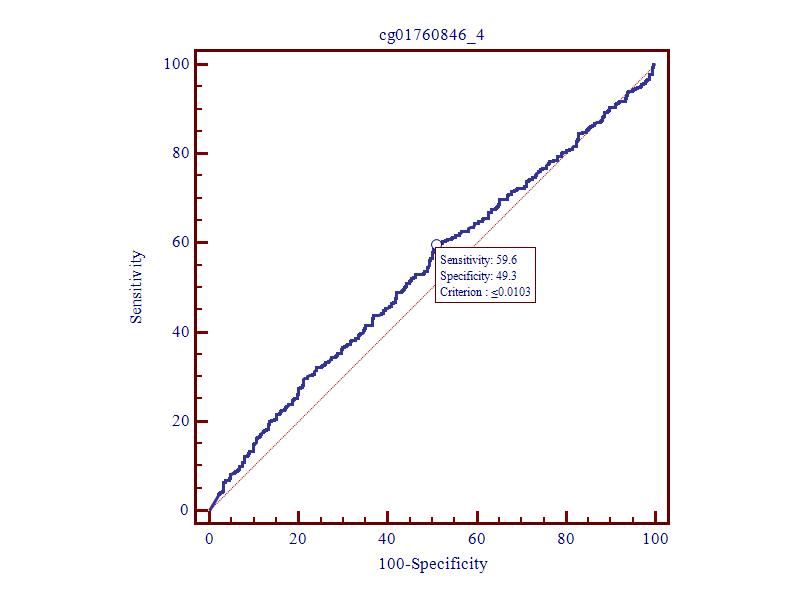
 Figure S12. ROC curves of PSMC1_90722795.**

**
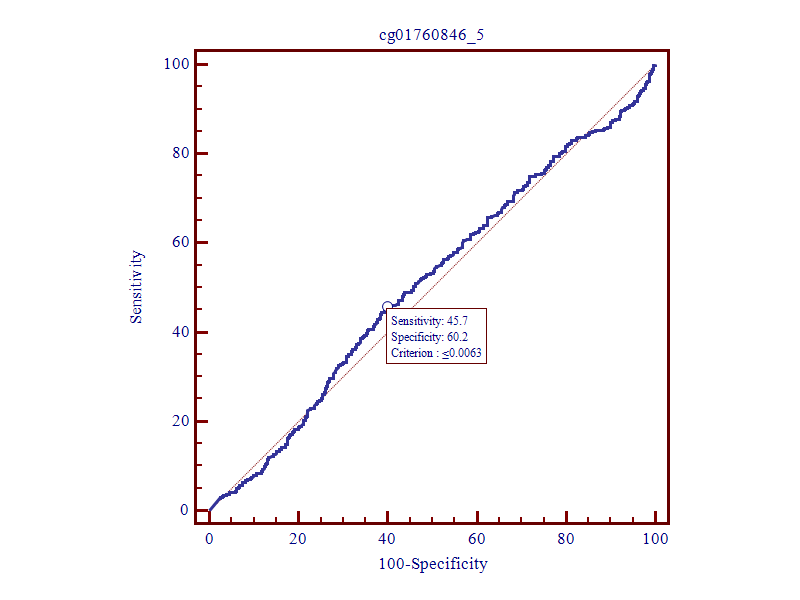
**

**Figure S13. ROC curves of PSMC1_90722799
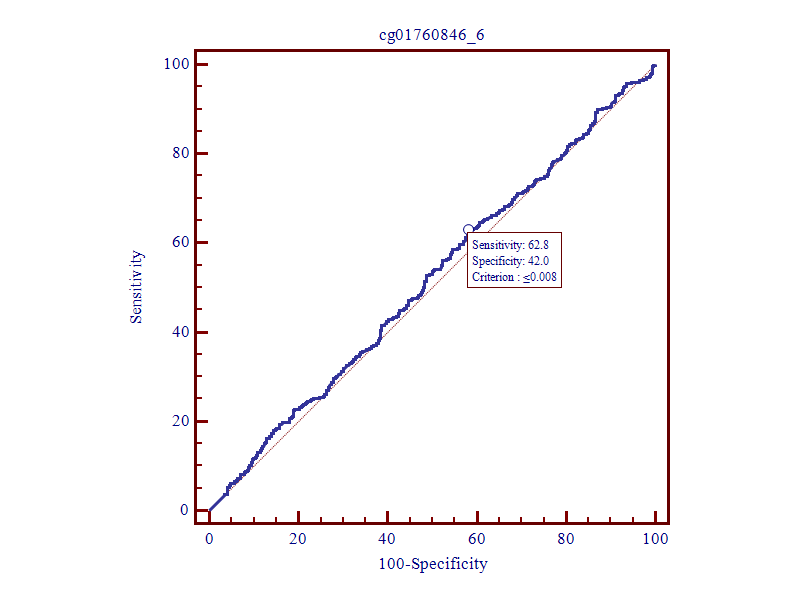
 Figure S14. ROC curves of PSMC1_90722830.**

**
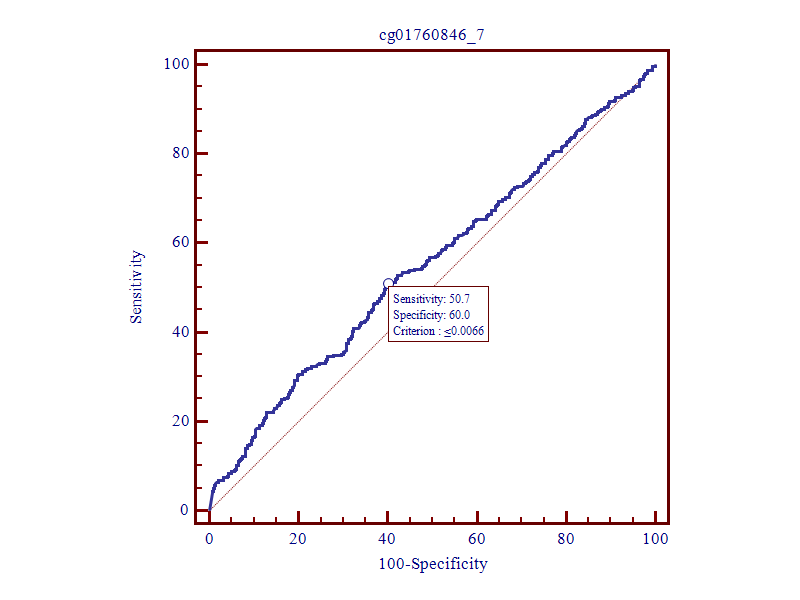
**

**Figure S15. ROC curves of PSMC1_90722856.**

**
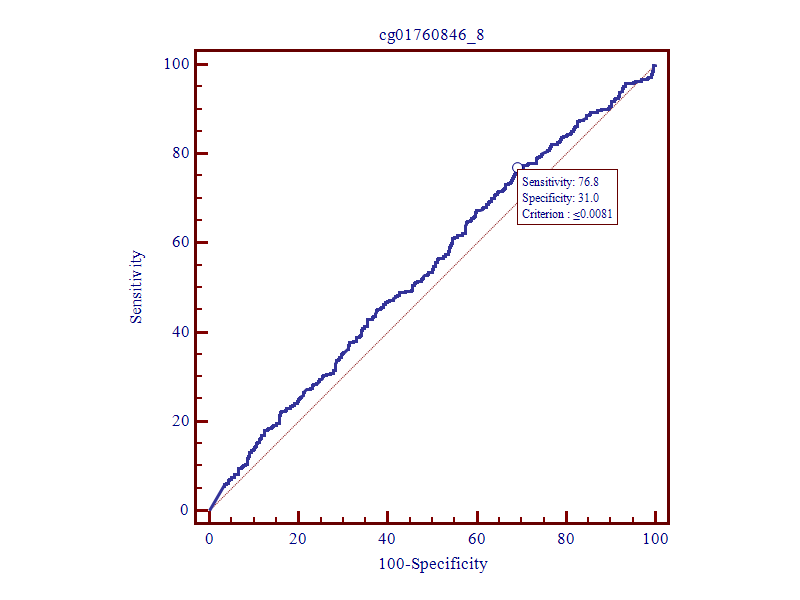
**

**Figure S16. ROC curves of PSMC1_90722861.
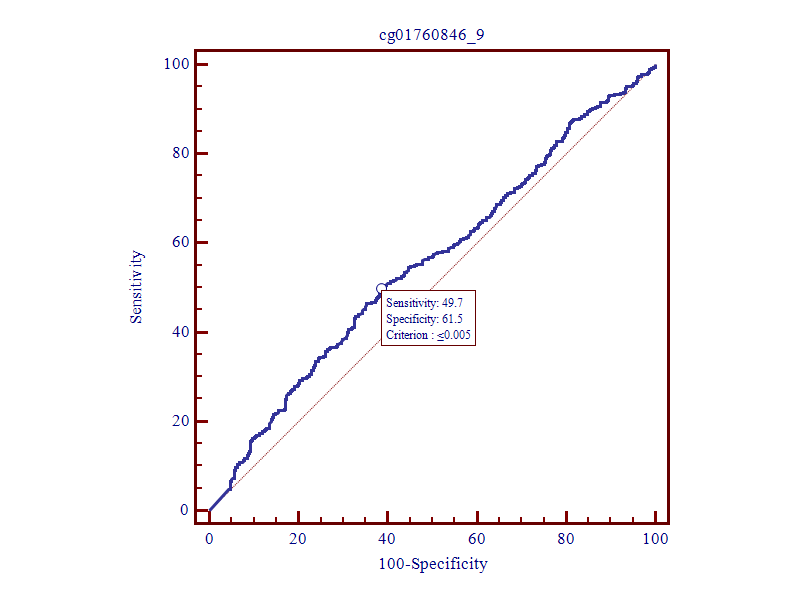
**

**Figure S17. ROC curves of PSMC1_90722870.
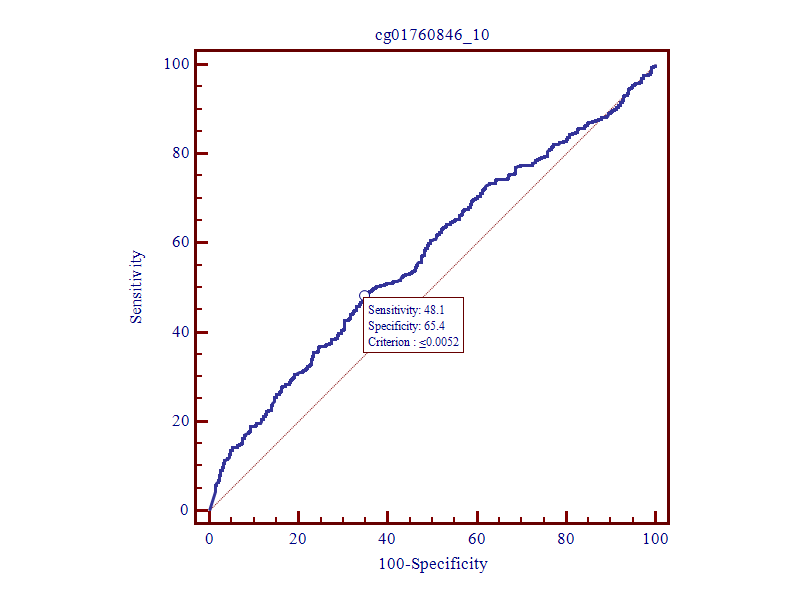
**

**Figure S18. ROC curves of PSMC1_90722877.
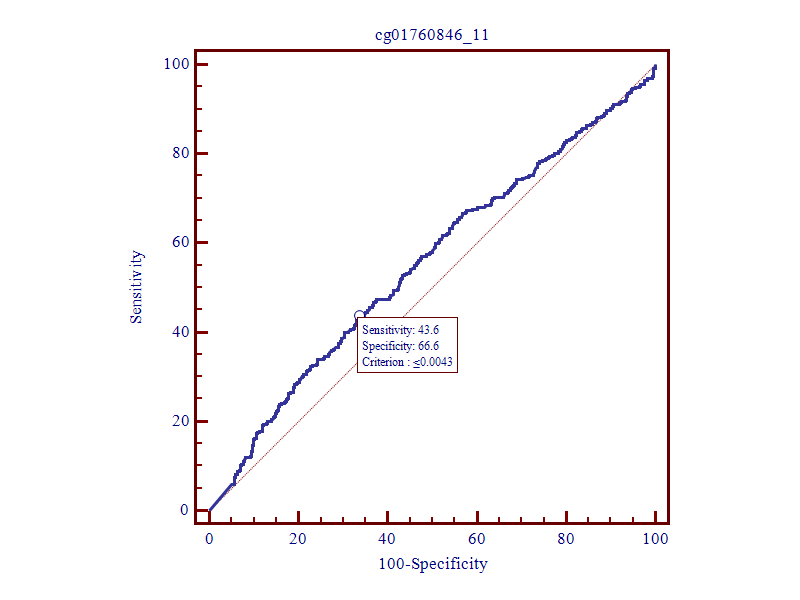
**

**Figure S19. ROC curves of PSMC1_90722886.
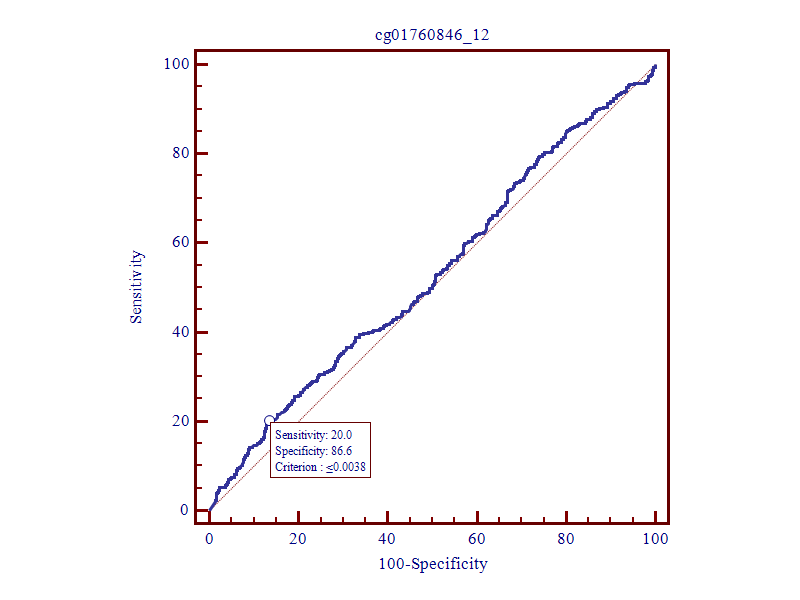
**

**Figure S20. ROC curves of PSMC1_90722891.
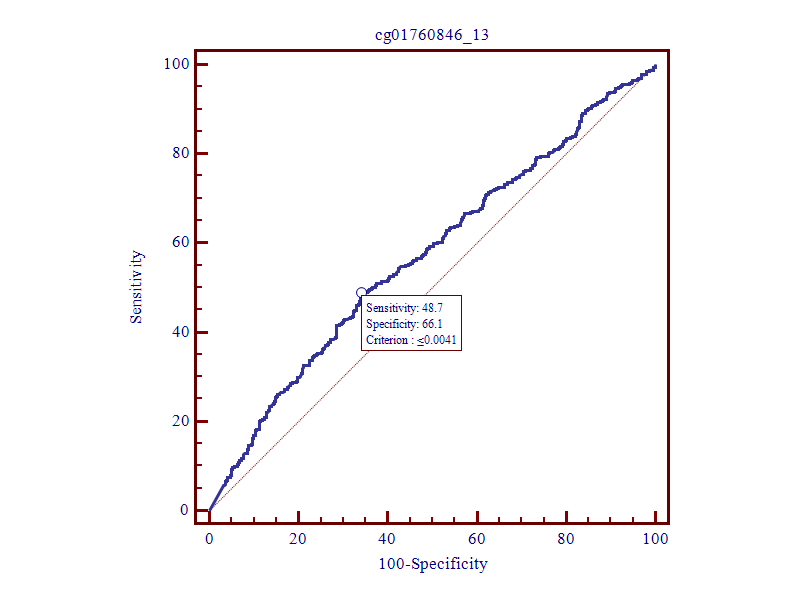
**

**Figure S21. ROC curves of PSMC1_90722911.**

**
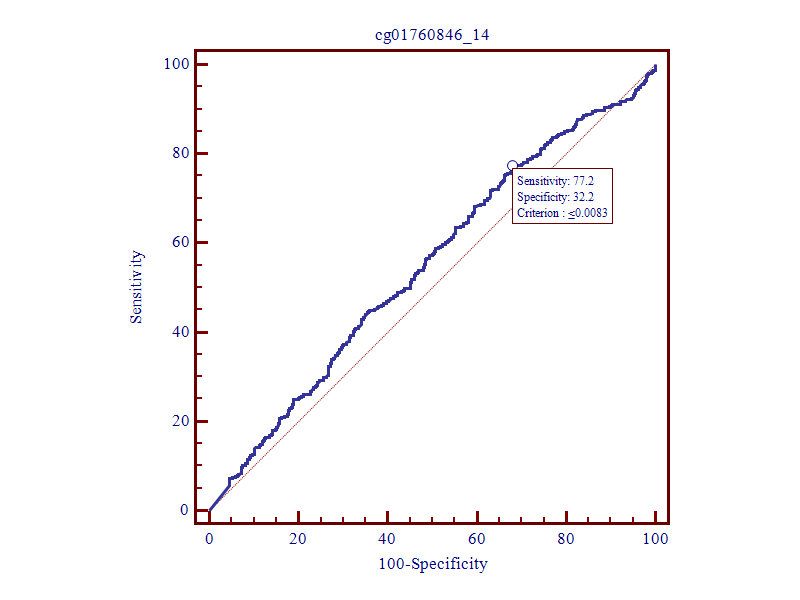
**

**Figure S22. ROC curves of PSMC1_90722917.
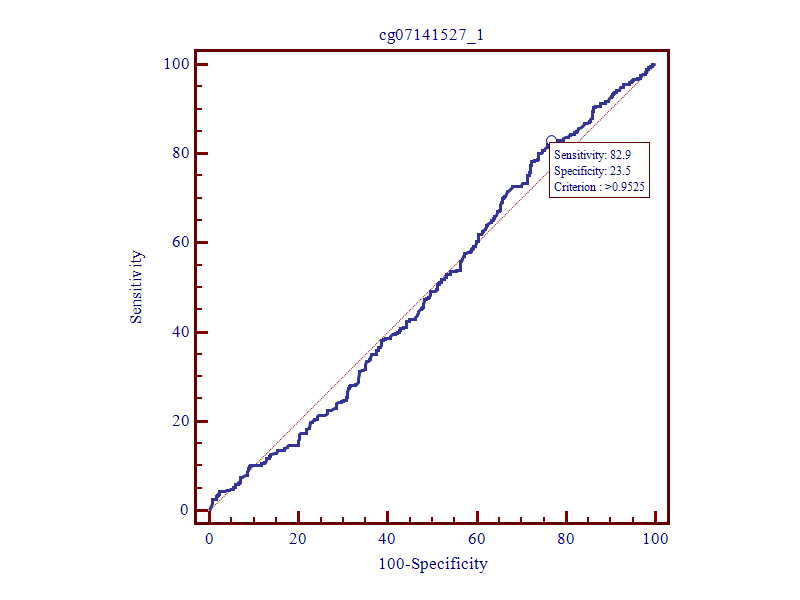
**

**Figure S23. ROC curves of SPPL3_121202409**

**
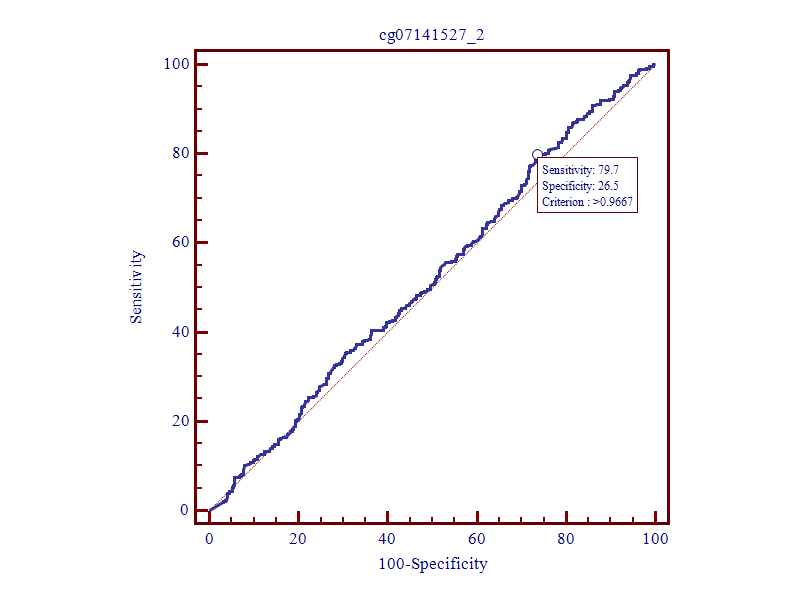
 Figure S24. ROC curves of SPPL3_121202464**

**
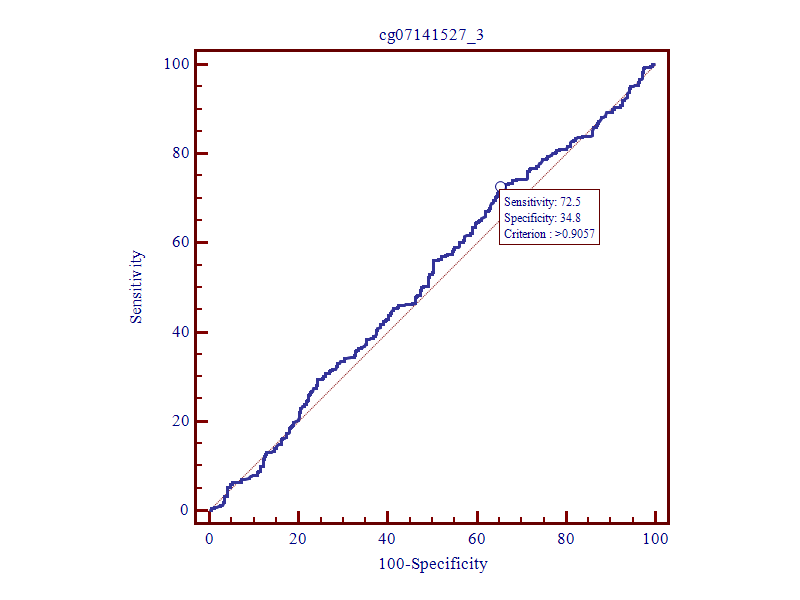
**

**Figure S25. ROC curves of SPPL3_121202539.**

**
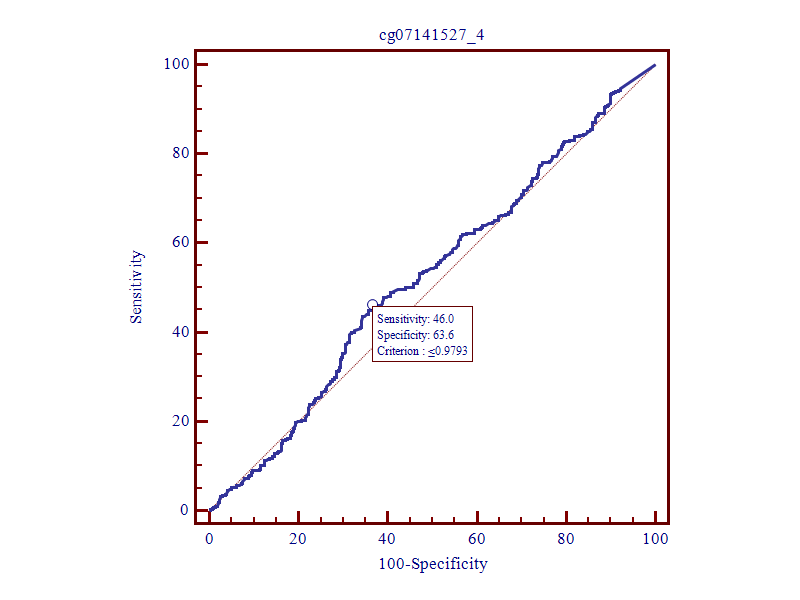
**

**Figure S26. ROC curves of SPPL3_121202552.**

**
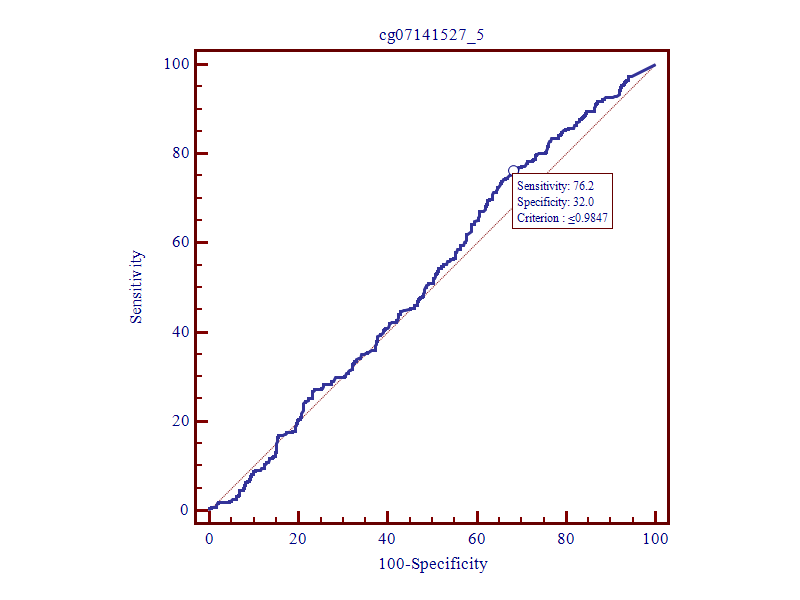
**

**Figure S27. ROC curves of SPPL3_121202554.**

**
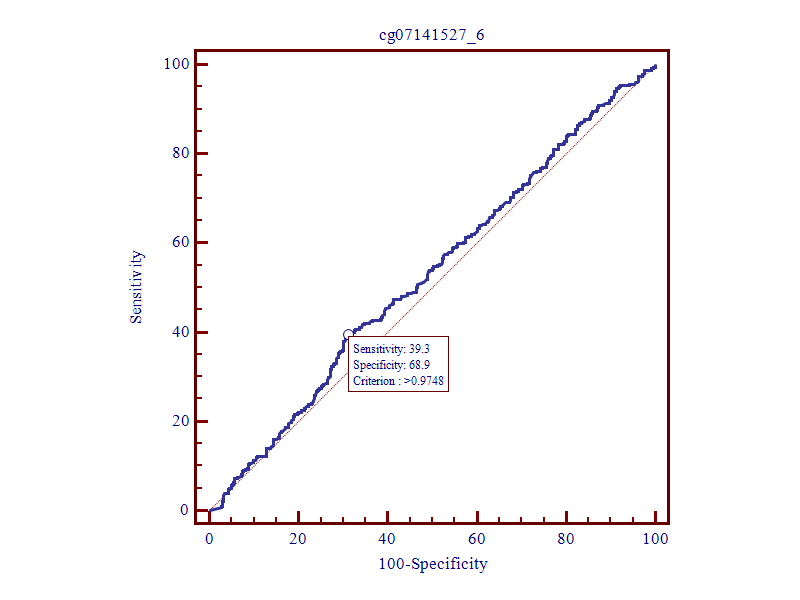
**

**Figure S28. ROC curves of SPPL3_121202602.
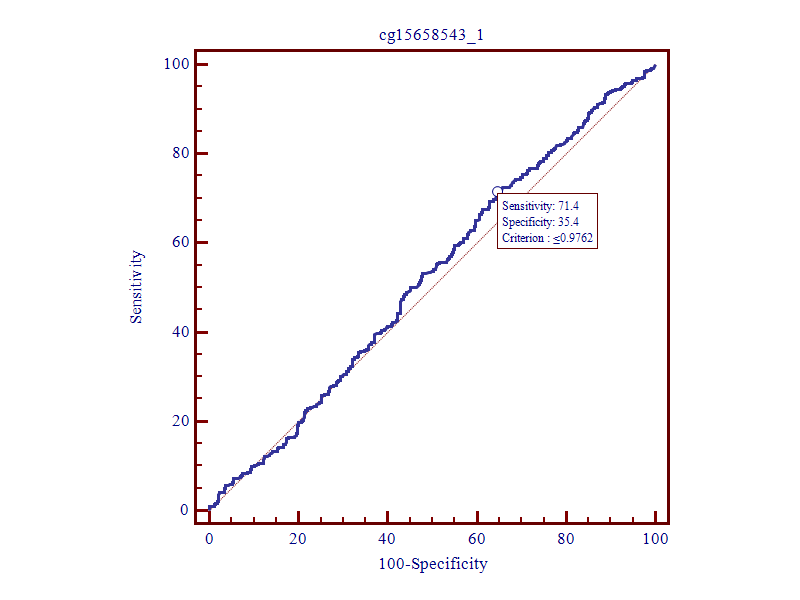
**

**Figure S29. ROC curves of CARD11_3026478.**

**
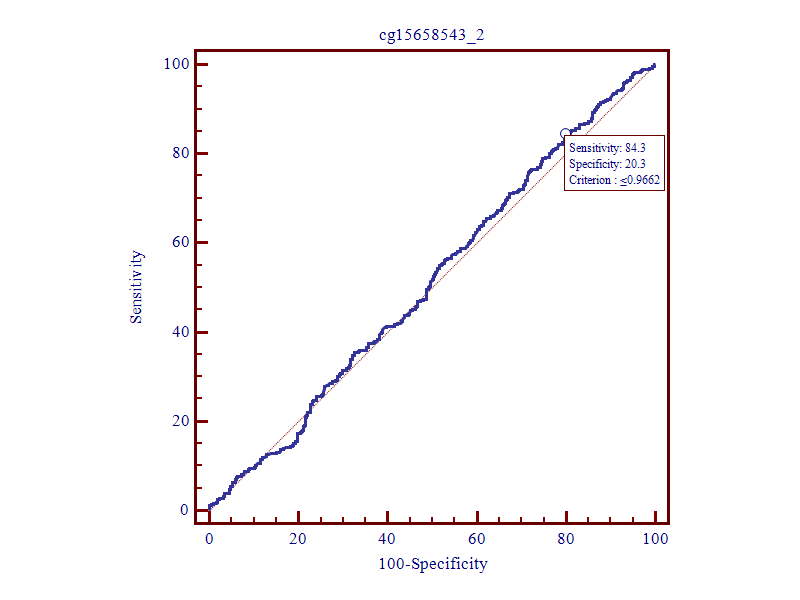
**

**Figure S30. ROC curves of CARD11_3026468**

**.
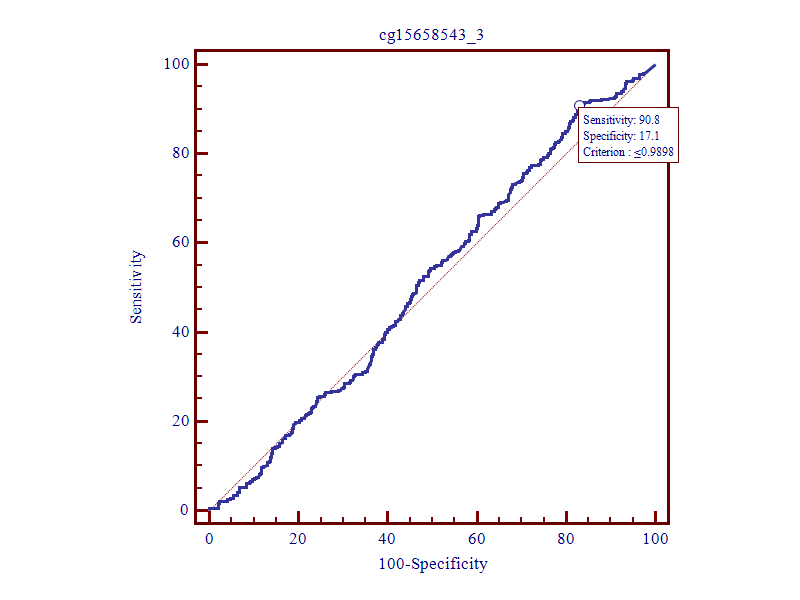
**

**Figure S31. ROC curves of CARD11_3026460.**

**
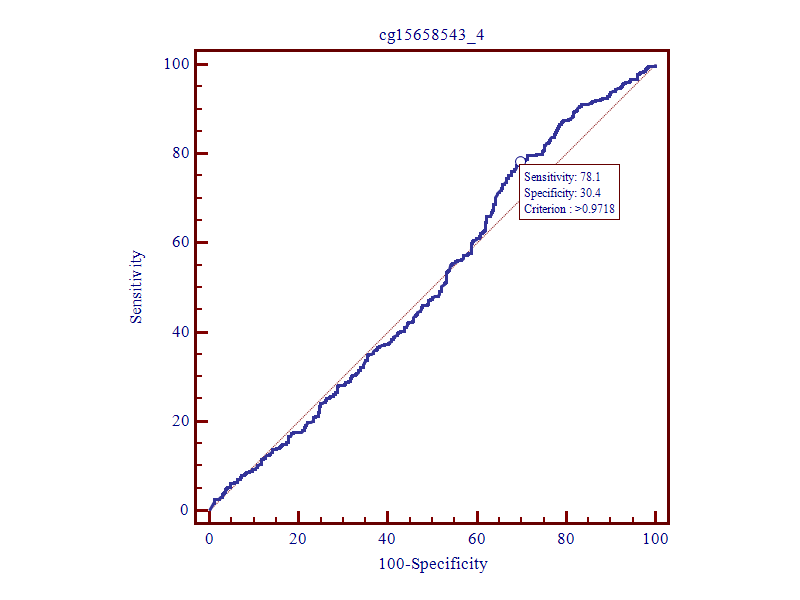
**

**Figure S32. ROC curves of CARD11_3026436.
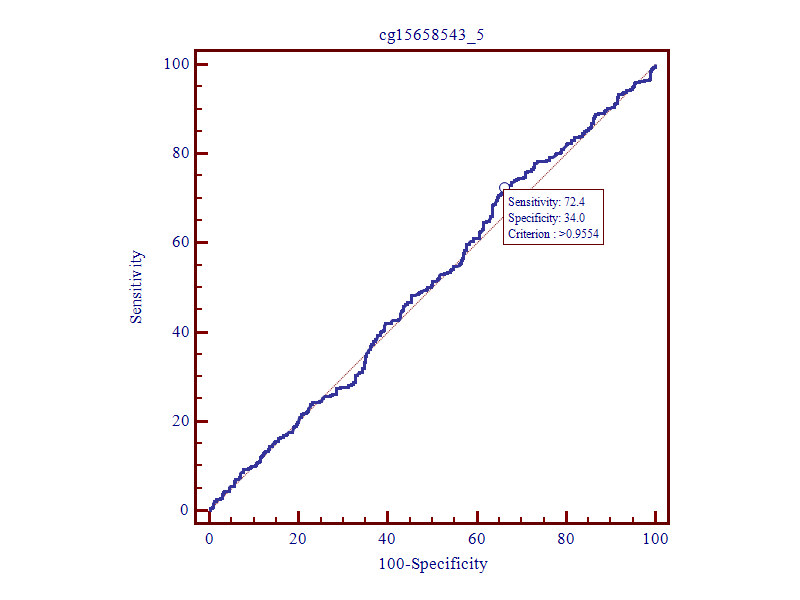
**

**Figure S33. ROC curves of CARD11_3026433.**

**
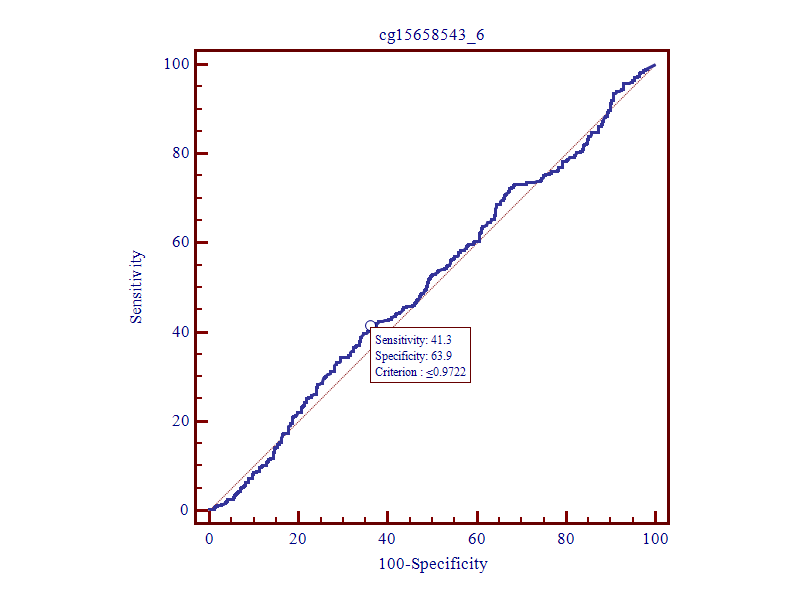
**

**Figure S34. ROC curves of CARD11_3026413.
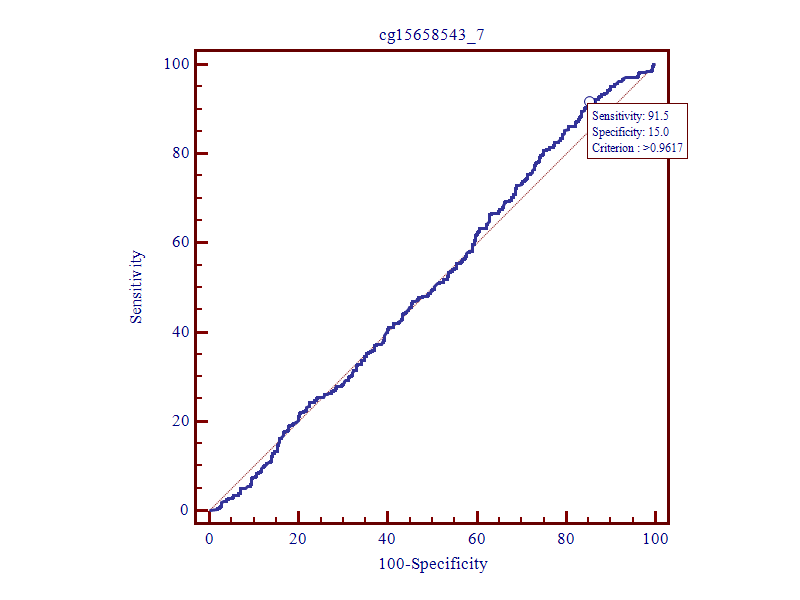
**

**Figure S35. ROC curves of CARD11_3026389.
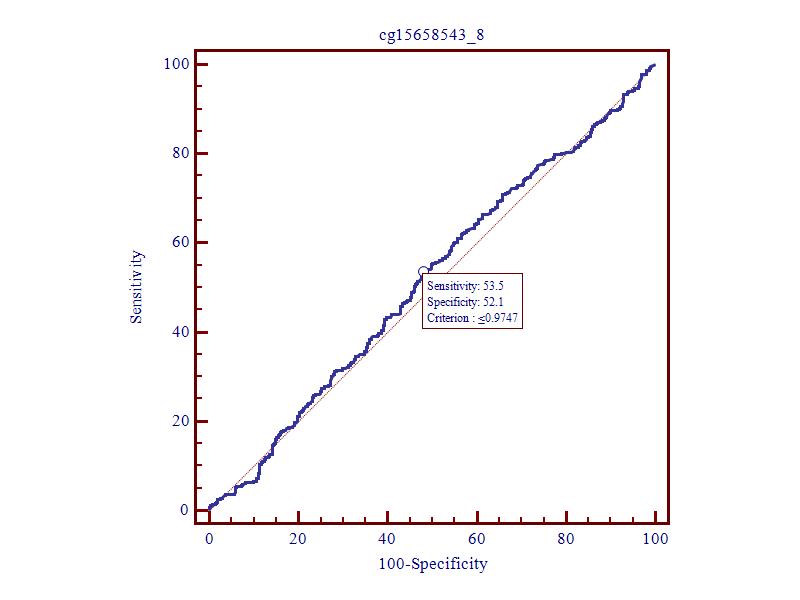
 Figure S36. ROC curves of CARD11_3026380.
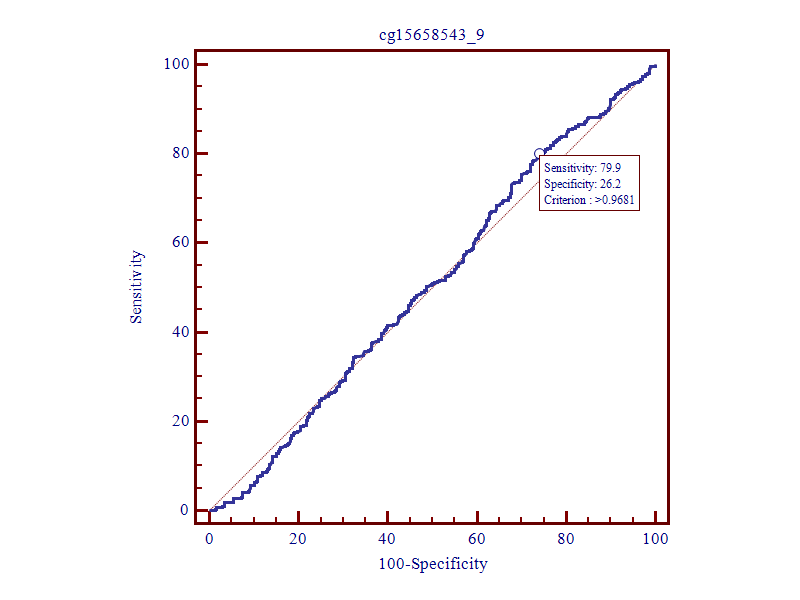
 Figure S37. ROC curves of CARD11_3026375.
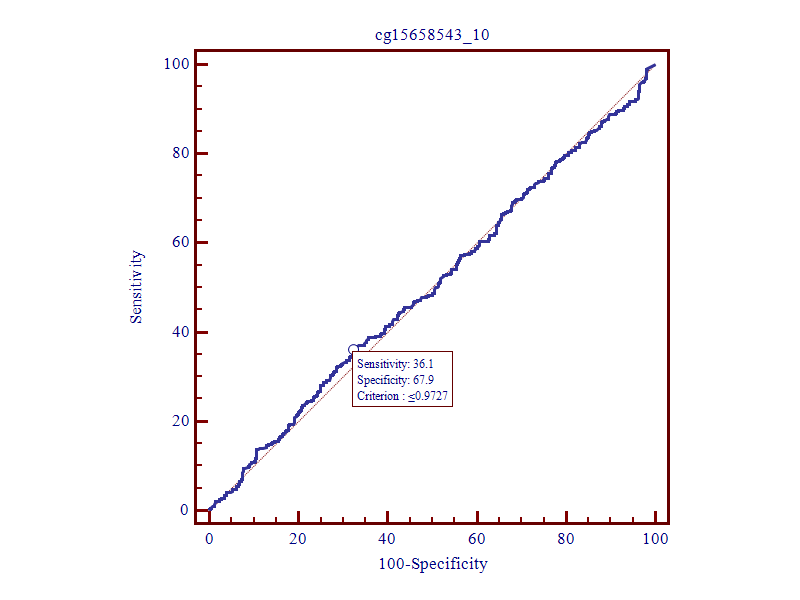
 Figure S38. ROC curves of CARD11_3026348.
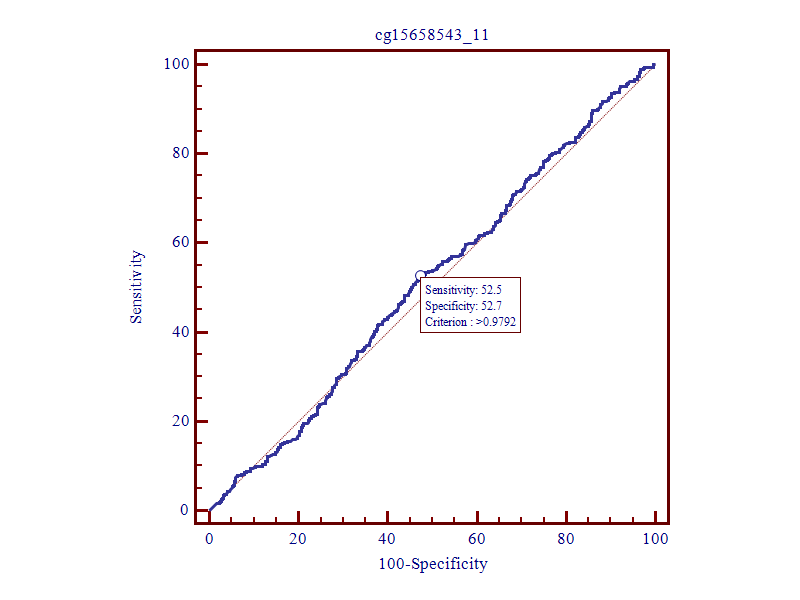
**

**Figure S39. ROC curves of CARD11_3026326
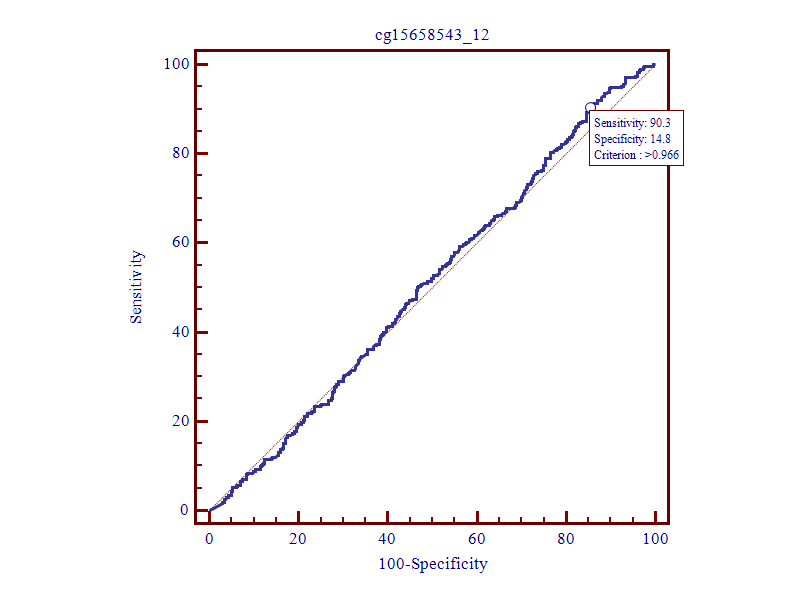
 Figure S40. ROC curves of CARD11_3026321.
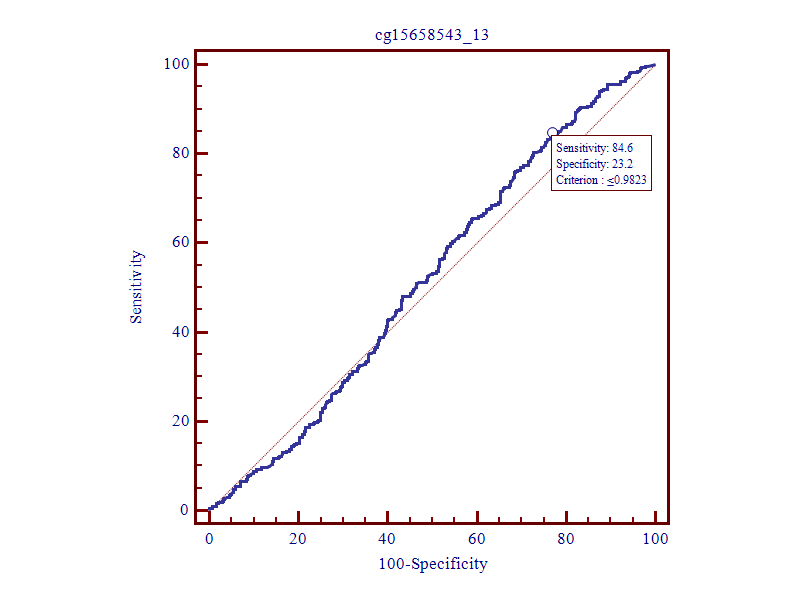
 Figure S41. ROC curves of CARD11_3026317.
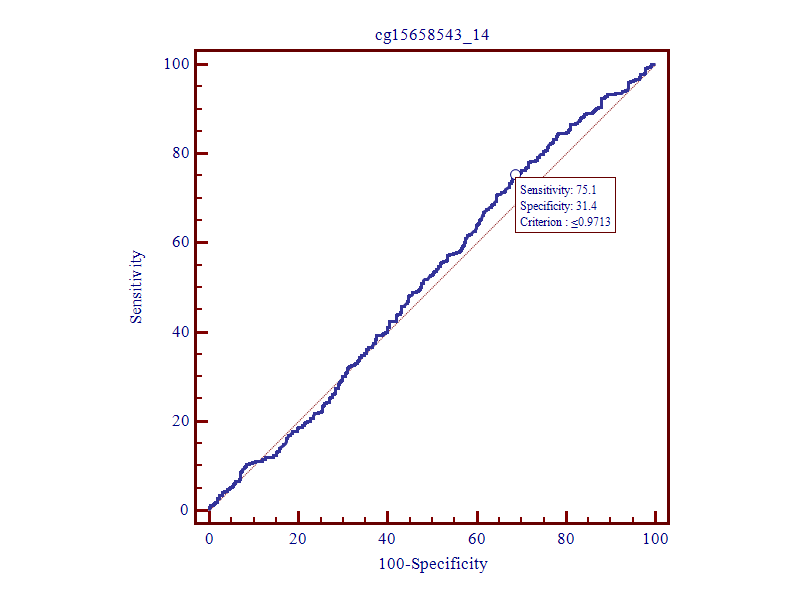
 Figure S42. ROC curves of CARD11_3026310.
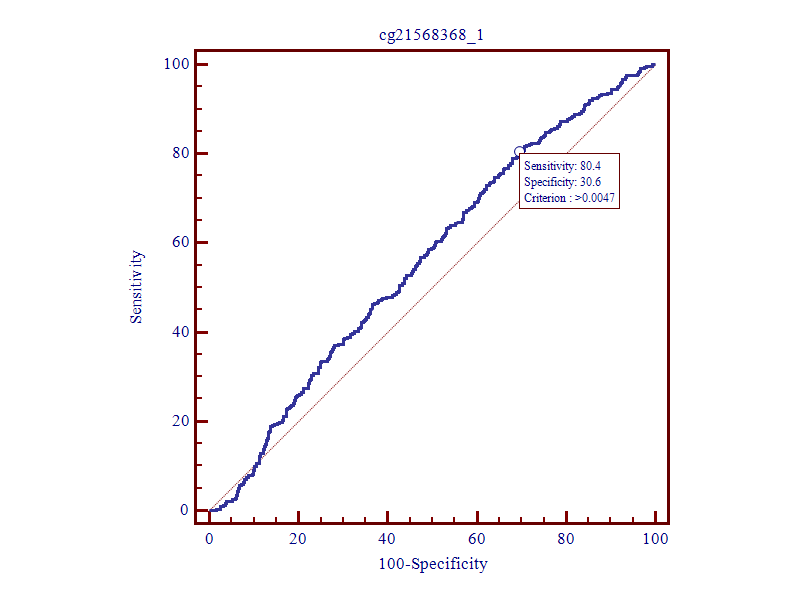
**

**Figure S43. ROC curves of PSMB8_32812098
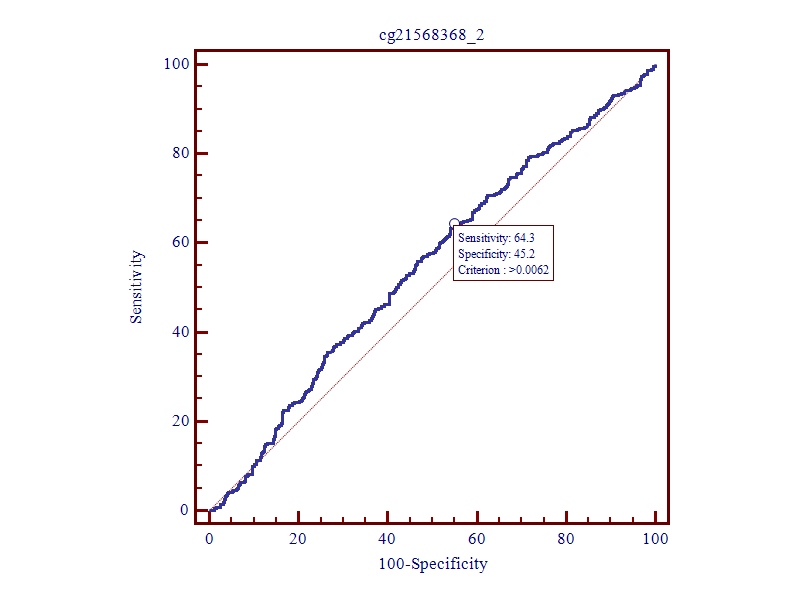
 Figure S44. ROC curves of PSMB8_32812113.
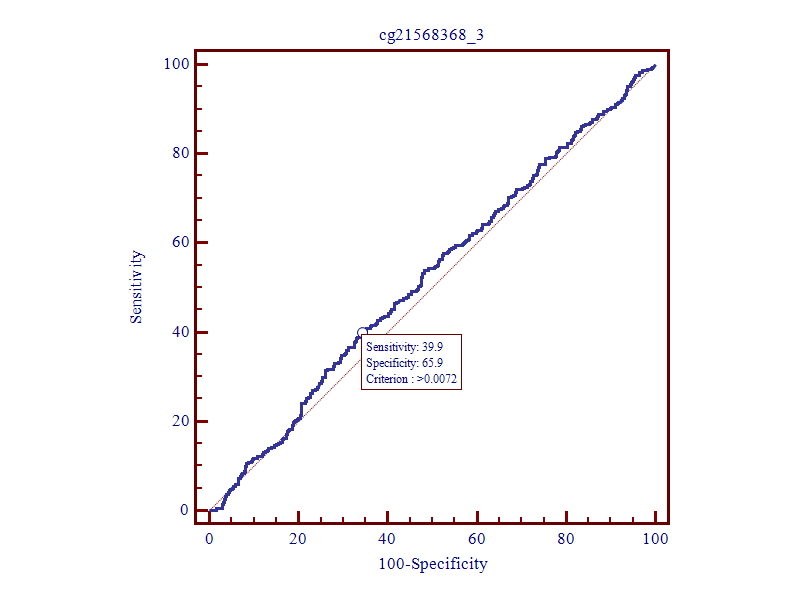
**

**Figure S45. ROC curves of PSMB8_32812165.
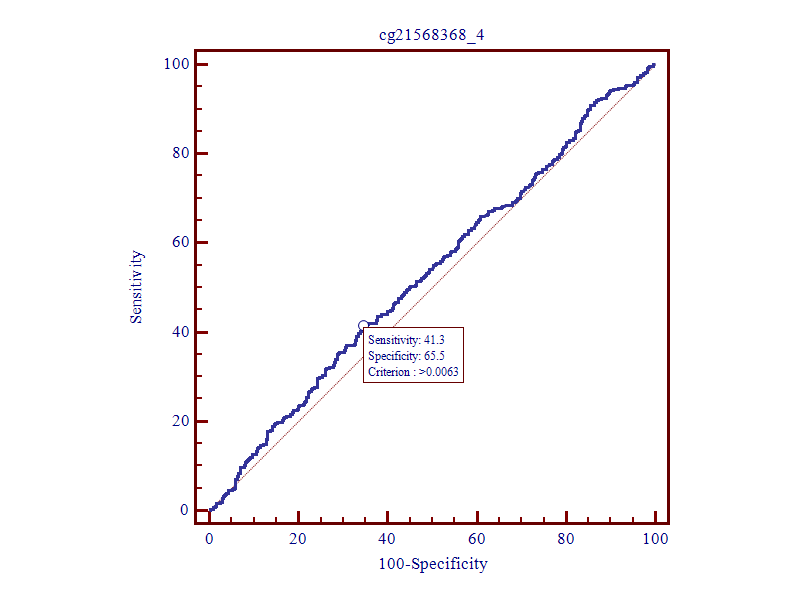
 Figure S46. ROC curves of PSMB8_32812167.
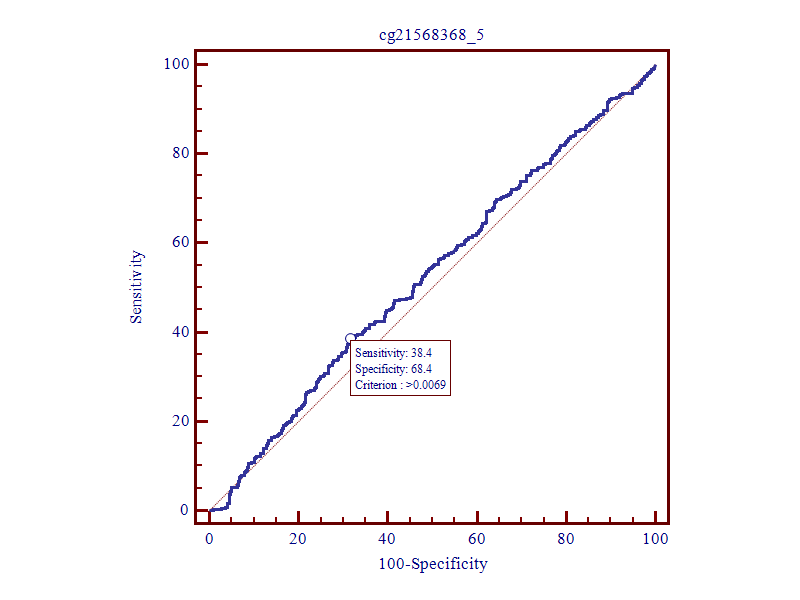
**

**Figure S47. ROC curves of PSMB8_32812213.
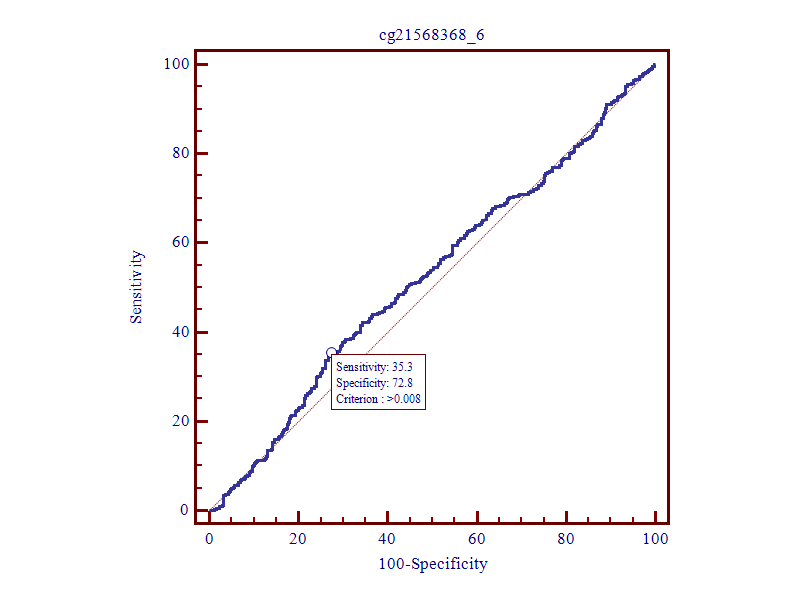
 Figure S48. ROC curves of PSMB8_32812221.
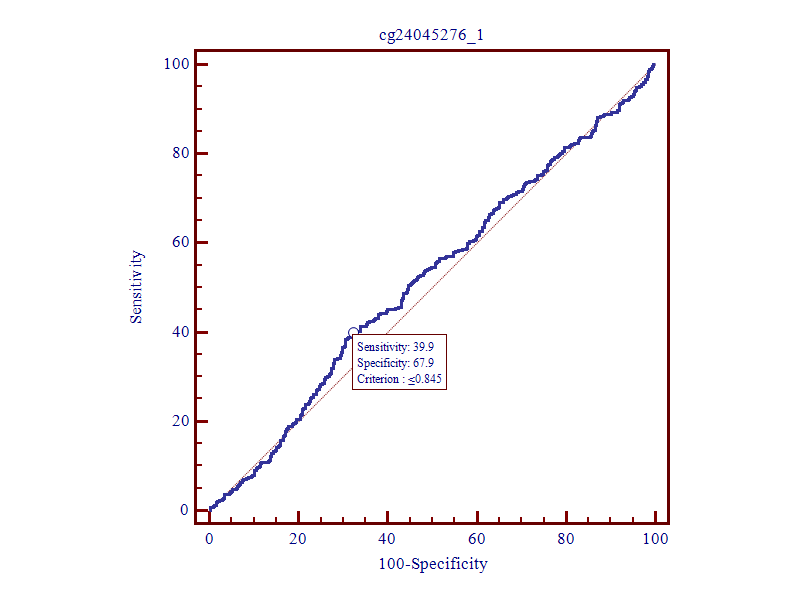
 Figure S49. ROC curves of NCF2_183551942.**

**
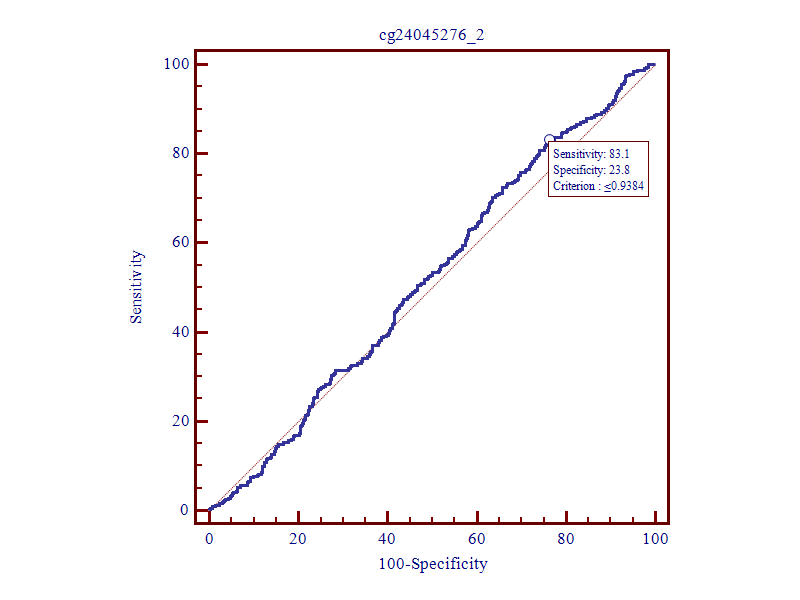
**

**Figure S50. ROC curves of NCF2_183551969.
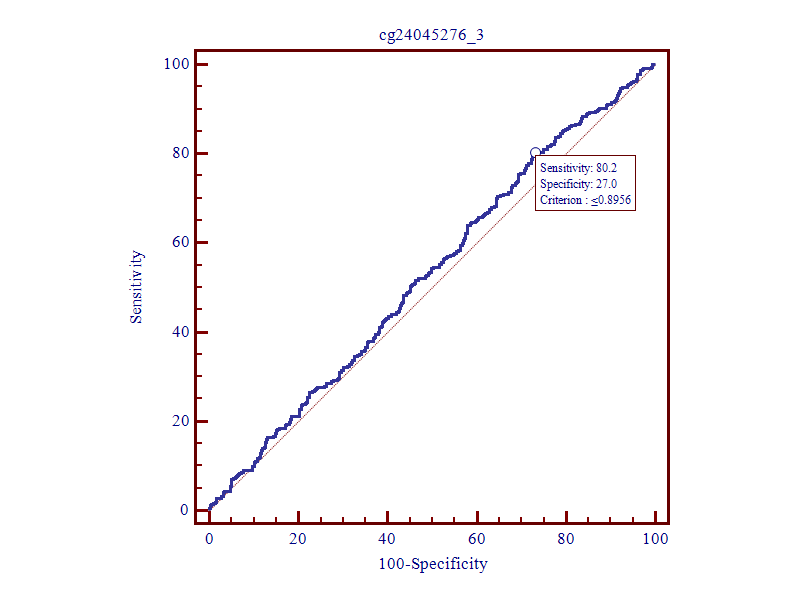
**

**Figure S51. ROC curves of NCF2_183551986.**

**
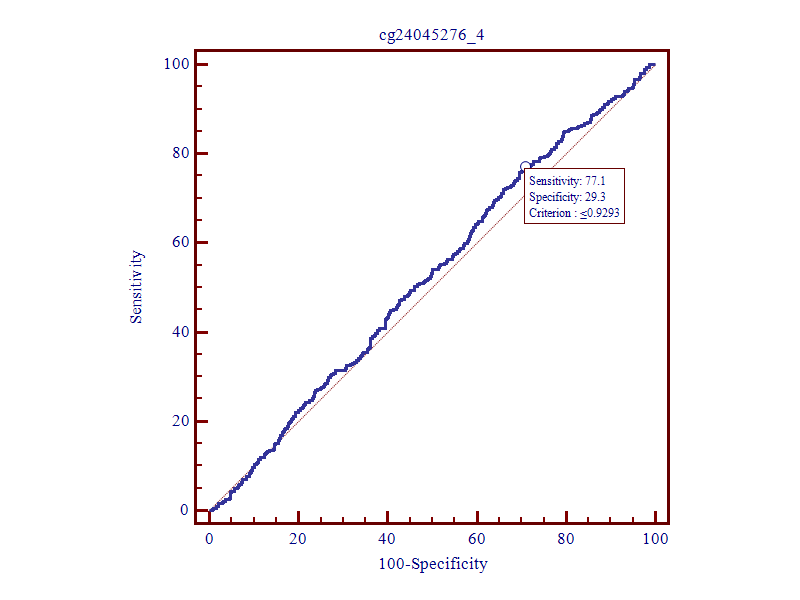
 Figure S52. ROC curves of NCF2_183552072
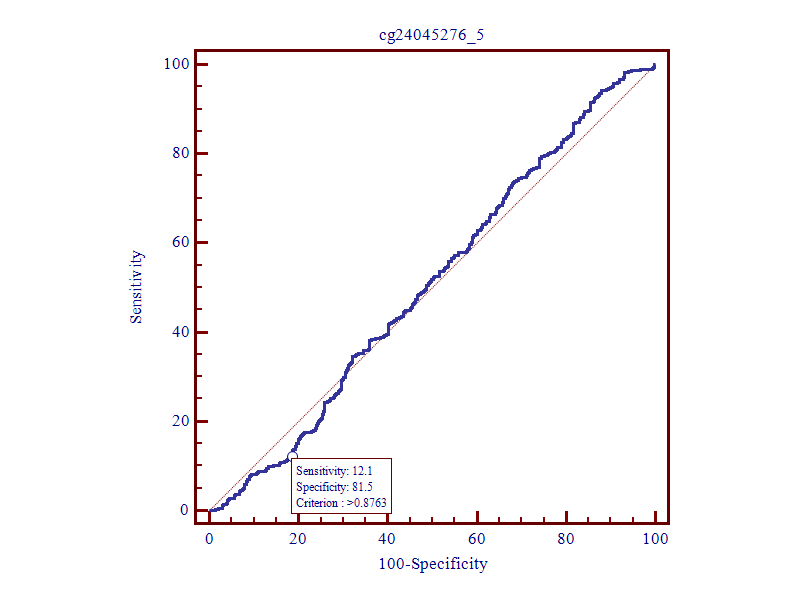
**

**Figure S53. ROC curves of NCF2_183552095.**

**
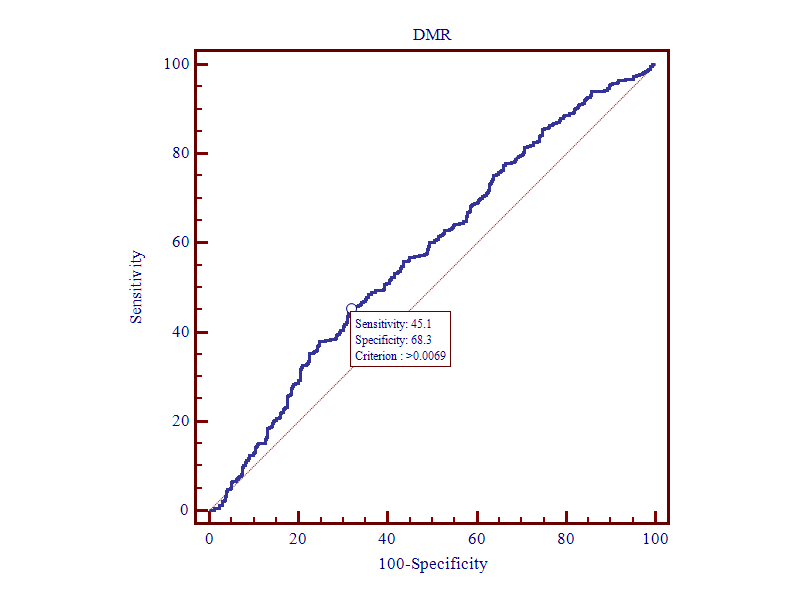
 Figure S54. ROC curves of PSMC1_DMR.
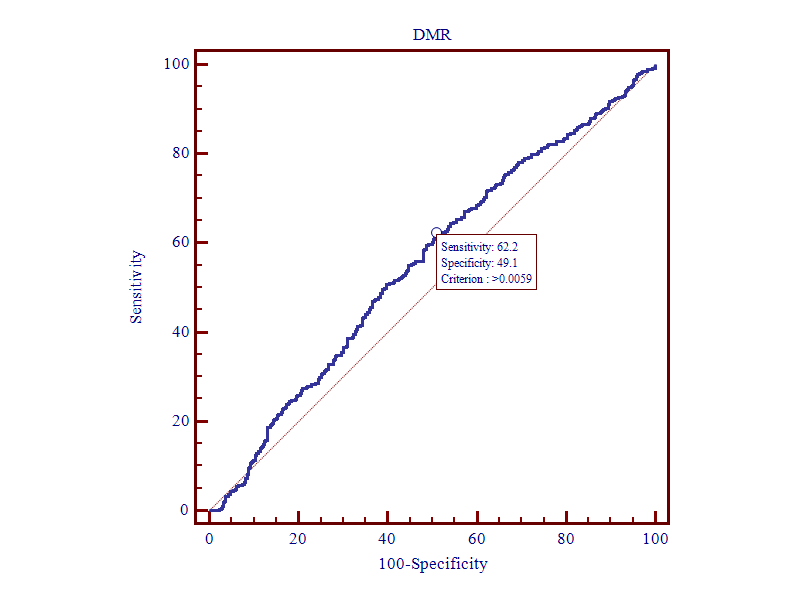
 Figure S55. ROC curves of PSMB8_DMR.**

**
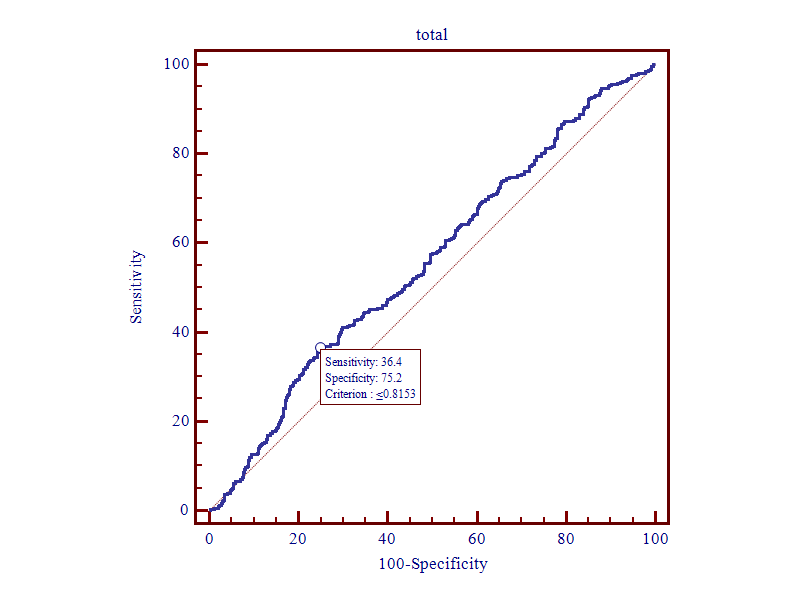
**

**Figure S56. ROC curves of PSMC1_Haplot.**

**
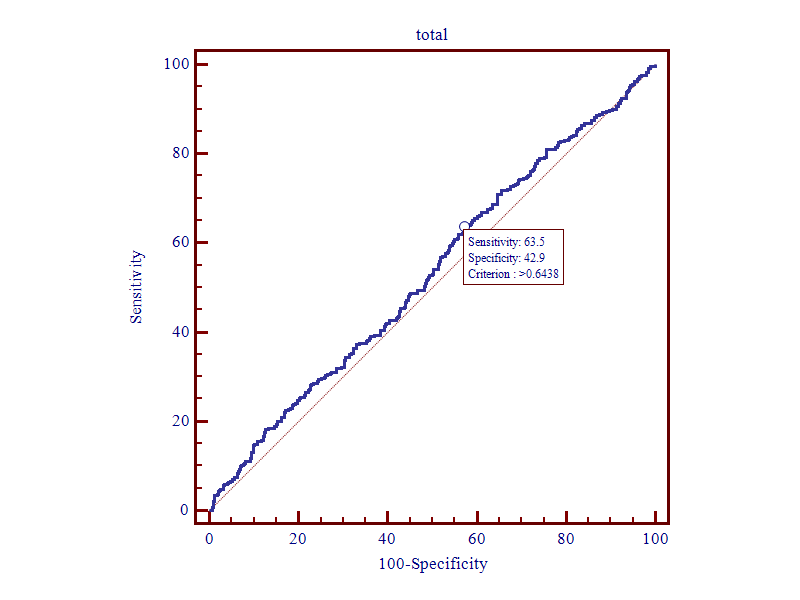
 Figure S57. ROC curves of SPPL 3_Haplot.**

**
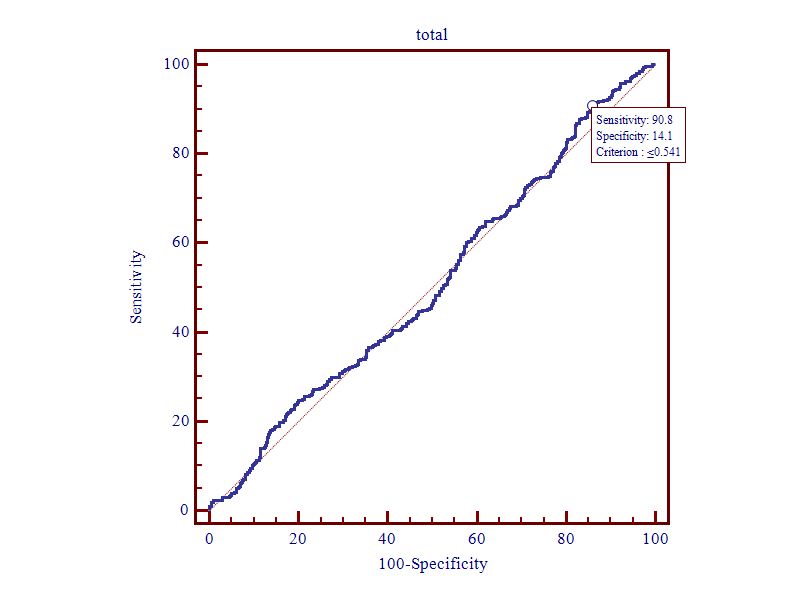
**

**Figure S58. ROC curves of CARD11_Haplot.**

**
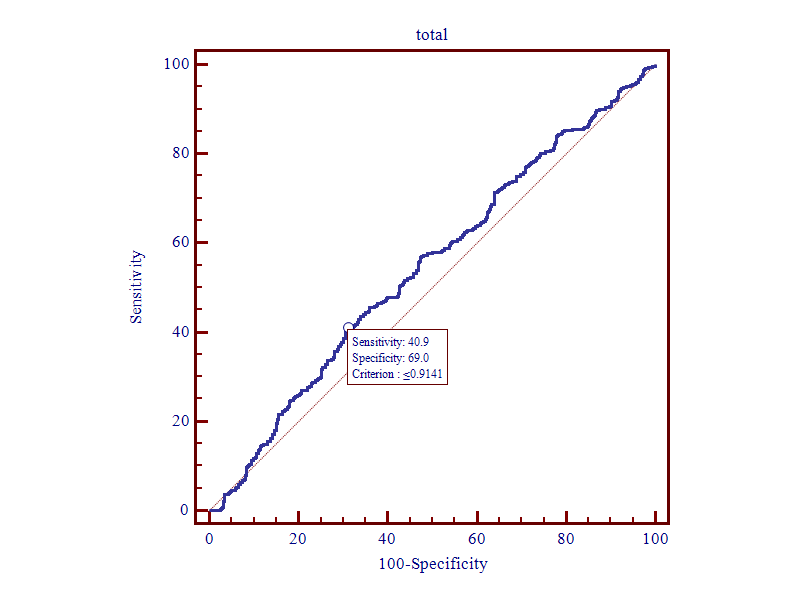
**

**Figure S59. ROC curves of PSMB8_Haplot.**

**
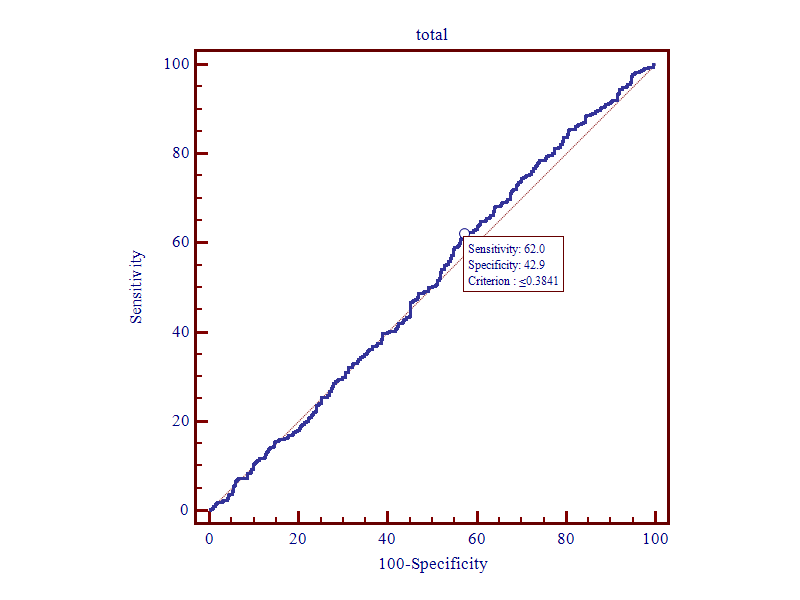
 Figure S60. ROC curves of NCF2_Haplot.**
